# Functional Traits From Imaging Spectroscopy Inform Patterns of Forest Mortality During Sierra Nevada Drought

**DOI:** 10.1111/gcb.70246

**Published:** 2025-05-15

**Authors:** Natalie Queally, Ting Zheng, Zhiwei Ye, Kyle R. Kovach, Ryan Pavlick, Ethan Shafron, Fabian D. Schneider, Philip A. Townsend

**Affiliations:** ^1^ Department of Forest and Wildlife Ecology University of Wisconsin‐Madison Madison Wisconsin USA; ^2^ Jet Propulsion Laboratory California Institute of Technology Pasadena California USA; ^3^ Department of Ecosystem and Conservation Sciences University of Montana Missoula Montana USA; ^4^ Department of Biology, Section for Ecoinformatics and Biodiversity Aarhus University Aarhus Denmark; ^5^ Pioneer Center for Landscape Research in Sustainable Agricultural Futures (Land‐CRAFT) Aarhus Denmark

**Keywords:** AVIRIS‐Classic, drought, forest mortality, functional traits, imaging spectroscopy

## Abstract

California's 2012–2016 megadrought led to the mortality of over 100 million trees. In the context of extreme drought and insect outbreaks, a holistic view of plant functional traits can provide further insight into underlying physiological and abiotic drivers of the patterns of mortality. We used new maps of early‐drought (pre‐mortality) foliar functional traits derived from the NASA AVIRIS‐Classic imaging spectrometer, along with open‐access climate, topography, canopy structure, and mortality data, to assess competing influences on drought mortality at the Soaproot Saddle and Lower Teakettle NEON sites in the southern Sierra Nevada Mountains. We aimed to (1) compare mortality trends across two independently derived mortality datasets, (2) assess trait‐mortality relationships across diverse sites and species, and (3) link these relationships to mechanisms of tree‐level drought response. We used random forests to assess the relative importance of mortality drivers and the trends of mortality across each predictor gradient. For the lower elevation, more water‐limited Soaproot Saddle site, conifer mortality was linked to taller, drier canopies while broadleaf mortality was linked to foliar traits (lower cellulose, higher sugars, and higher leaf mass per area). For the higher elevation, more energy‐limited Lower Teakettle site, mortality was more strongly linked to elevation and climate, with little influence from foliar traits.

## Graphical abstract

We used plant trait information to improve understanding of forest mortality patterns following the 2012–2016 California megadrought. We found that mortality related to traits associated with photosynthesis, leaf hardiness, and forest structure.

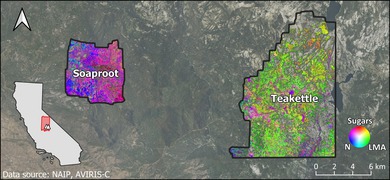

## 1 Introduction

California experienced a severe to exceptional drought from 2012–2016 (USDA and NOAA 2023; Williams et al. 2022), uniquely characterized by its concurrent extremes of low precipitation and high temperature (AghaKouchak et al. 2014; Crockett and Westerling 2018; Lund et al. 2018; Mann and Gleick 2015). The U.S. Forest Service estimates that 129 million trees died during this period, with mortality highly concentrated in the central and southern Sierra Nevada region and driven by both drought and insect outbreaks (Moore et al. 2018). Severe canopy water losses were also observed for 58 million large trees, indicating a high risk for mortality (Asner et al. 2016). Extensive loss of the largest trees in the forest has concerning environmental consequences, including loss of crucial habitat for wildlife (Jones et al. 2018; Meyer et al. 2005) and long‐term declines in aboveground carbon stocks and forest stand productivity (Pfeifer et al. 2011). Community composition may shift toward hardwood and shade‐tolerant conifer species that existed in the understory (Fettig et al. 2019; Young et al. 2020), and the increase in dry fuels can lead to more severe fire outbreaks (Stephens et al. 2018), causing further tree mortality (Hemming‐Schroeder et al. 2023). In the context of a changing climate, it is expected that drought will become more frequent and severe in many parts of the world (Dai 2013), including California (Diffenbaugh et al. 2015; Wahl et al. 2022). This increases the need to better understand the drivers of drought‐related tree mortality across broad landscapes.

Hydraulic failure and carbon starvation are widely considered the primary mechanisms of tree mortality during drought. Hydraulic failure occurs when xylem function is lost through embolism of the vascular tissue, impeding water transport and causing plant desiccation (Hartmann et al. 2013; McDowell et al. 2008; Sevanto et al. 2014). Carbon starvation occurs due to stomatal closure, when the limited amount of carbohydrates produced from photosynthesis is not sufficient to meet demands for plant maintenance and function (McDowell et al. 2008). However, the two are not mutually exclusive, as carbohydrate transport under drought may be impeded by hydraulic stress (Sala et al. 2010). Meta‐analyses have shown that hydraulic failure is a more consistent predictor across species and biomes, while evidence for carbon starvation is not universal (Adams et al. 2017; Anderegg et al. 2016). These two hypotheses are typically assessed via two functional traits: percent loss of xylem conductivity and changes in tissue nonstructural carbohydrates (sugars and starch; NSC), respectively (Adams et al. 2017). Broadening our view to include additional plant functional traits may provide further insight into underlying physiological and abiotic drivers of the patterns of mortality.

Plant functional traits, the measurable characteristics of organisms that can be linked to plant functions (Díaz et al. 2016; Maynard et al. 2022; Wright et al. 2004), have the potential to characterize ecosystem function and community response to global change (Lavorel and Garnier 2002; Shipley et al. 2016; Suding et al. 2008). Foliar traits in particular provide insight into plant resource investment and adaptive strategies to stress (Sack and Buckley 2020; Wright et al. 2004), and traits relating to leaf photosynthetic capacity, structure, storage, and defense could buffer drought effects (Table 1). Morphological traits like tree height provide insight into forest structure and, in the context of this drought, are strongly linked to insect host preferences (Stephenson et al. 2019). The integration of physiological and morphological traits in the compounding presence of both drought‐induced physiological stress *and* insect outbreaks can help characterize mortality patterns, where insects may either kill the most drought‐stressed trees or kill healthy trees that possess favorable traits (Stephenson et al. 2019).

**TABLE 1:** Description of mapped foliar traits used in this study.

Trait function	Traits from AVIRIS‐classic	Ecological importance
Nutrient concentration, photosynthetic capacity	Nitrogen, leaf mass per area (LMA)	Higher foliar nitrogen relates to increased primary production and nutrient cycling in the ecosystem (De Bello et al. 2010; Lavorel and Garnier 2002). Higher LMA increases leaf longevity and resistance to stress (Wright et al. 2004)
Structure, leaf longevity	Lignin, cellulose	Higher investment in structural components increases leaf strength and toughness, and improves resistance to environmental stress (Alonso‐Forn et al. 2023)
Storage, transport	Sugars, starch (nonstructural carbohydrates; NSC)	Higher foliar NSC reflects more carbon stores to be distributed across plant tissues. NSCs are sensitive to drought (Hartmann and Trumbore 2016; McDowell et al. 2008), and fluctuations may reflect altered regulatory functions (McDowell 2011)
Pigment, photosynthetic capacity	Chlorophyll A/B	Higher chlorophyll content increases the capacity to absorb light for photosynthesis. Stressful conditions such as drought may cause decline in pigments (Carter and Knapp 2001; Tucker 1980)
Defense	Phenolics	As a product of secondary metabolism, decreases in production of phenolics could reflect a shift toward prioritizing metabolic maintenance during stress (De Bello et al. 2010; Niinemets 2016)

Data availability has limited which plant traits are included in multi‐trait models for drought mortality predictions, and studies recognize the importance of expanding the scope of traits in future work (Anderegg et al. 2016; Fernández‐de‐Uña et al. 2023). Thus far, both field‐based studies and remote sensing‐based studies of drought mortality in the Sierra Nevada have measured a small selection of traits, with tree size/stand structure proving particularly important in relation to insect host preference (Hemming‐Schroeder et al. 2023; Restaino et al. 2019; Stephenson et al. 2019; Stovall et al. 2019). Canopy water content (CWC) has also been identified as an important predictor (Asner et al. 2016; Brodrick and Asner 2017), relating to suppressed growth and photosynthesis under water stress (Chaves et al. 2002). Otherwise, studies have mostly highlighted important abiotic predictors such as climate (Robbins et al. 2022; Stovall et al. 2019), topography (Das et al. 2022; Paz‐Kagan et al. 2017), and deep soil moisture (Goulden and Bales 2019). At present, we lack an understanding of how physiological traits of leaves contribute to the susceptibility of trees to mortality under drought conditions. Improving this understanding could help identify early warning signs of tree mortality risk in the event of severe drought.

This work expands on previous studies by including pre‐mortality foliar functional trait measurements derived from imaging spectroscopy. Imaging spectroscopy, or hyperspectral remote sensing, has become a central technology for understanding the dynamics of vegetation, including foliar functional traits (Asner et al. 2015; Singh et al. 2015; Wang et al. 2020). We leveraged trait maps derived from the Airborne Visible InfraRed Imaging Spectrometer (AVIRIS)‐Classic (Green et al. 1998) to characterize potential physiological factors affecting susceptibility to mortality at two US National Ecological Observatory Network (NEON) sites using published tree mortality data (Hemming‐Schroeder et al. 2023; Stovall et al. 2019). We aimed to (1) compare mortality trends across two independently derived mortality datasets, (2) assess trait‐mortality relationships across diverse sites and species, and (3) link these relationships to mechanisms of tree‐level drought response.

## 2 Methods

### 2.1 Study Area

**FIGURE 1:**
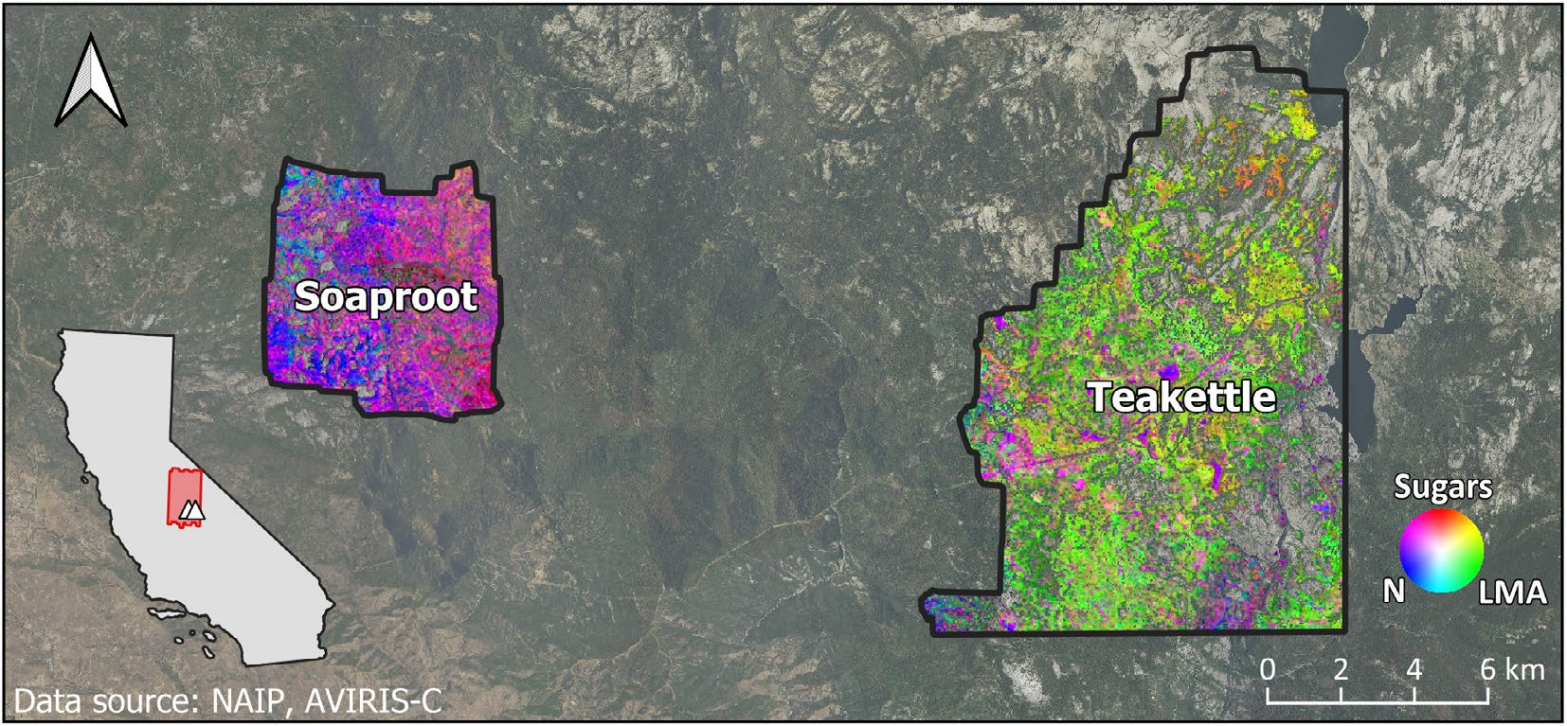
This three‐trait composite shows the relative values of leaf mass per area (LMA), foliar nitrogen (N), and foliar sugars across the two NEON sites. Differences in color across the three‐trait gradient highlight varying functional strategies across the landscape, with clear contrast between the two sites. For example, the prevalence of blue and magenta at Soaproot Saddle indicate relatively high foliar investment in nitrogen, or nitrogen and sugars, respectively. In contrast, the prevalence of bright green and yellow at Lower Teakettle indicate relatively high LMA, or LMA and foliar sugars, respectively. The site locations (triangles) and AVIRIS‐Classic flight box (red box) are shown in the California state inset.

Soaproot Saddle and Lower Teakettle are located in the southern Sierra Nevada Mountains of California (Figure 1), an area identified as a hotspot of tree mortality during the drought (Moore et al. 2018). Both sites are regularly measured as part of NEON Domain 17 (Pacific Southwest) and have published tree mortality data (Hemming‐Schroeder et al. 2023; Stovall et al. 2019) and co‐occurring NASA AVIRIS‐Classic imagery (Lee et al. 2015) from 2013–2016. Soaproot Saddle is a mixed conifer forest, spanning 1000–1400 m and dominated by ponderosa pine ( *Pinus ponderosa* ), incense cedar ( *Calocedrus decurrens* ), and canyon live oak ( *Quercus chrysolepis* ) (Kunch 2019). Due to its lower elevation, Soaproot Saddle is a water‐limited site, where growth responds strongly to water availability (Das et al. 2013). Lower Teakettle is a higher elevation mixed conifer forest, spanning 1900–2907 m and dominated by red fir ( *Abies magnifica* ), white fir ( *Abies concolor* ), and lodgepole pine ( *Pinus contorta* ) (Kunch 2022). Notably, Teakettle sits at the transition zone of water‐limited (growth responds strongly to water availability) to energy‐limited (growth responds strongly to temperature changes) forest, which occurs around 2100–2600 m in the Sierra Nevada (Das et al. 2013; Trujillo et al. 2012). As such, growth in the lower elevation range responds more strongly to water availability, while growth in the higher range responds more strongly to temperature changes. Teakettle experiences cooler temperatures, higher precipitation, and more snowpack than Soaproot (Kunch 2019, 2022).

### 2.2 Data Preparation

#### 2.2.1 Trait Maps

Foliar functional trait maps for each year from 2013–2016 were developed using partial least squares regression (PLSR) models, a common method for estimating traits from imaging spectroscopy data, and follow methodology from Singh et al. (2015) and Wang et al. (2020). The maps were derived from AVIRIS‐Classic imaging spectroscopy data acquired for a larger region (the “Yosemite box”) as part of the Western Diversity Time Series (WDTS) imaging effort (Lee et al. 2015) (Figure 1). A more detailed modeling workflow is shown in Figure S1. A subset of five flightlines from the Yosemite box fully covered the two NEON sites for most of the drought period, with imagery acquired in June of each year from 2013–2016. Because tree mortality was relatively low until 2015 (Fettig et al. 2019; Hemming‐Schroeder et al. 2023; Moore et al. 2018; Stovall et al. 2019), we tested 2013 and 2014 traits as the “pre‐mortality” state. Since the 2013 and 2014 acquisitions occurred during similar phenological periods (Figure S2), phenology was not expected to affect the “pre‐mortality” state across years.

Foliar traits were selected for this study based on ecological importance, availability from field measurements, and detectability through imaging spectroscopy. Further detail of the function and ecological importance of each trait is included in Table 1. Of the nine traits modeled, chlorophyll, LMA, nitrogen, and NSC shared comparable validation *R* ^2^ values with previous studies (Wang et al. 2020; Cherif et al. 2023; Singh et al. 2015; Zheng et al. 2024). Though models for phenolics, starch, sugars, lignin, and cellulose exhibited lower *R* ^2^ values, normalized RMSE values were comparable to the other studies. Compared to studies with higher spatial resolution (e.g., 1 m in Wang et al. (2020)), the lower *R* ^2^ values are likely attributed to mixed spectra and mixed species composition associated with the coarser spatial resolution of AVIRIS‐Classic pixels (15 m). PLSR model performance is shown in Figure S3 and Table S1.

Trait maps were mosaicked, resampled, and registered to the 30 × 30 m Landsat grid using AROSICS local co‐registration (Scheffler et al. 2017). Co‐registering the images ensured the consistent geographic location of pixels across years, allowing direct pixel‐to‐pixel comparison across images to link mortality data to the trait maps through time. The 30 m resolution is also compatible with the resolution of other data sources of interest used in this study, including canopy water content (CWC; Brodrick and Asner 2017), elevation and topography (van Zyl 2001), and soil moisture (Vergopolan et al. 2021). Likewise, the resolution is comparable to the proposed resolution for the forthcoming satellite mission Surface Biology and Geology (SBG), which will provide global coverage of hyperspectral data with a repeat time of 16–21 days (Cawse‐Nicholson et al. 2021; Stavros et al. 2023).

#### 2.2.2 Mortality Data

Tree mortality data from Stovall et al. (2019) were accessed from figshare (https://doi.org/10.6084/m9.figshare.7609193.v1). The authors assessed individual crown mortality percent (%_cm_) using Lidar and National Agriculture Imagery Program (NAIP) Orthoimagery. During the drought period, %_cm_ was assessed for all trees in 2012, 2014, and 2016 due to NAIP image availability. To match the trait map resolution, we took the average %_cm_ of all trees whose centroid falls within a 30 m pixel, yielding the pixelwise percent mortality.

We also accessed the mortality dataset from Hemming‐Schroeder et al. (2023), which provides an updated mortality assessment for Soaproot and Teakettle. The authors assessed individual %_cm_ using LiDAR and NEON Airborne Observation Platform (AOP) data. During the drought period, %_cm_ was assessed for trees in 2013 and 2017 due to NEON image availability. The aggregated 30 m resolution map of tree mortality was accessed on Zenodo (https://doi.org/10.5281/zenodo.7812035). Both Hemming‐Schroeder et al. (2023) and Stovall et al. (2019) focused on individual tree mortality, but we analyze per‐pixel percent mortality at the 30 m level to match the resolution of our trait maps. As such, our “percent mortality” reflects the extent of crown mortality (%_cm_), rather than a binary dead/alive classification.

Using mortality estimates from two studies enabled us to test whether trends in mortality were consistent across independently derived data products. Because tree mortality was relatively low in the area until 2015 (Fettig et al. 2019; Hemming‐Schroeder et al. 2023; Moore et al. 2018; Stovall et al. 2019), we only analyzed 2016 and 2017 mortality estimates from Stovall et al. and Hemming‐Schroeder et al., respectively.

#### 2.2.3 Other Data

Past studies analyzing drought‐related tree mortality found height (Stovall et al. 2019), topography (Das et al. 2022; Paz‐Kagan et al. 2017), climate (Robbins et al. 2022; Stovall et al. 2019), CWC (Asner et al. 2016; Brodrick and Asner 2017), soil moisture (Goulden and Bales 2019), and species composition (Restaino et al. 2019; Stephenson et al. 2019) to be important predictors of drought‐related mortality in the Sierra Nevada. We assembled data representing these covariates (Table S2) to compare their influence against the influence of foliar functional traits.

#### 2.2.4 Climate

Daymet data for mean, minimum, and maximum air temperature and cumulative precipitation were downloaded from the Oak Ridge National Laboratory Distributed Active Archive Center (ORNL DAAC) for 2012–2016 (Thornton et al. 2022). The corresponding 30‐year climate normals (1970–2000) were downloaded from WorldClim (Fick and Hijmans 2017). To encompass the climate conditions during drought, variables were first represented as some summary of that time: each temperature variable was represented as the mean across all years, while cumulative precipitation was represented as the total cumulative value across all years. Once these summaries were calculated, we calculated the percent change of these summaries relative to the 30‐year climate normals. We referred to these as precipitation and temperature anomalies, where a lower value indicates a lower than normal observation, and a higher value indicates a higher than normal observation. All climate variables were downloaded at 1 km resolution and resampled to 30 m using bilinear interpolation to match the trait maps and other covariates.

#### 2.2.5 Topography

Topography data were derived from a digital elevation model (DEM) from the Shuttle Radar Topography Mission (SRTM; van Zyl 2001), downloaded at 1 arc‐second (approximately 30 m) resolution on the USGS EarthExplorer. We used the DEM to calculate slope and aspect using the respective tools in ArcMap (v 10.5.1). From aspect, we calculated topographic “northness” (Equation 1) and “eastness” (Equation 2), indices that run from 1 to −1 for the east–west and north–south directions that relate more directly to morning/afternoon solar radiation and summer/winter solar radiation, respectively (Bader and Ruijten 2008). Since the dominant flow of air and moisture in the Sierra Nevada is from the Pacific Ocean (west), topographic eastness can also be seen as an indicator of dryness due to the rain‐shadow effect. We also used ArcMap to calculate flow accumulation (FA) from the DEM, which is later used to calculate the Topographic Convergence Index (TCI) (Equation 3), indicating the relative wetness of a site as a consequence of its topographic context.

(1) $$\text{Northness}=\cos\left(\text{aspect}\right)$$

(2) $$\text{Eastness}=\sin\left(\text{aspect}\right)$$

(3) $$\mathrm{TCI}=\ln\left(\frac{\mathrm{FA}}{\tan\left(\text{slope}\right)}\right)$$

#### 2.2.6 Soil Moisture

Soil moisture was obtained from the SMAP‐HydroBlocks (SMAP‐HB) dataset on Zenodo (https://doi.org/10.5281/zenodo.4441211; Vergopolan et al. 2021, 2022). SMAP‐HB is a 30 m resolution soil moisture map derived from land surface modeling, radiative transfer modeling, Soil Moisture Active Passive (SMAP) data, and in situ observations. SMAP‐HB data availability begins in 2015, so soil moisture is represented as the average soil moisture across 2015 and 2016 for this study. SMAP soil moisture sensitivity is limited to the top 5 cm of soil (Kumar et al. 2018), and thus does not account for deep soil moisture.

#### 2.2.7 Species Composition

Dominant tree species were derived from the USFS Existing Vegetation (EVeg) data for the “South Sierra” mapping zone (USDA—Forest Service 2016), using the classification system from the Society of American Foresters (SAF). SAF classifications include some species mixing within categories. For example, an area is considered “white fir” if white fir covers > 60% of the mapping unit (Fites et al. 1991). For simplicity, we will henceforth refer to these as “species”, while recognizing that some mixing may occur. Other groups contain a combination of species, such as “hard chaparral” and “mixed conifer”. According to SAF definitions and NEON site reports, the dominant species in the mixed chaparral class likely include manzanita (*Arctostaphylos* spp.) and mountain mahogany (*Cercocarpus* spp.) (Berg 1990; Kunch 2019, 2022), while the dominant species in mixed conifer can include white fir, ponderosa pine, sugar pine ( *Pinus lambertiana* ), incense cedar ( *Calocedrus decurrens* ), Jeffrey pine ( *Pinus jeffreyi* ), Douglas fir ( *Pseudotsuga menziesii* ), black oak (*Quercus kelloggii*), and red fir, depending on elevation (Fites et al. 1992). Data were accessed in vector form and rasterized to a high spatial resolution of 1 m, then aggregated to 30 m using a majority resampling technique. Therefore, each 30 m pixel represents the dominant species cover. Species were assessed separately for all classes with at least 400 pixels represented in the study area. At Soaproot, this included coast live oak ( *Quercus agrifolia* ), canyon live oak, mixed chaparral, ponderosa pine, and mixed conifer. At Teakettle, this included lodgepole pine, red fir, mixed conifer, and white fir.

#### 2.2.8 Other Traits

Other plant traits besides the AVIRIS‐C‐derived foliar traits include tree height, stand structure, and CWC. Tree height was calculated as the mean height of all trees within a 30 × 30 m pixel, with tree height data derived from the NEON canopy height model and distributed by Hemming‐Schroeder et al. (2023). These tree data were also used to derive proxies for stand structure: we calculated the standard deviation of height (henceforth called height SD) for each pixel, as well as the percent area of the pixel covered by tree crowns (henceforth called tree cover). These indicate whether trees in a pixel belong to a similar height class, and whether the pixel is densely forested. Finally, CWC (Brodrick et al. 2019), which represents the total amount of liquid water in a canopy, was retrieved from Pangaea (https://doi.pangaea.de/10.1594/PANGAEA.897276) at 30 m spatial resolution. Brodrick and Asner (2017) suggest that percent change in CWC from year to year is a better predictor of mortality than CWC alone. This change in CWC better reflects relative water dynamics in response to drought rather than reflecting water differences owed to the canopy characteristics like leaf area index (LAI) (Brodrick et al. 2019). To match the foliar trait years of interest, we tested percent change from pre‐drought (2011) to 2013, and from 2011 to 2014. For this metric, a negative value indicates a loss in CWC.

#### 2.2.9 Masking

All covariates were aligned to the Landsat grid using the Raster package in R (Hijmans 2023). We calculated NDVI for the 2013 and 2014 AVIRIS‐Classic images using the red and NIR bands at 665 and 850 nm, respectively, and masked out any pixels with NDVI < 0.5 in either year. This ensured that our pre‐mortality trait values were only being analyzed for highly vegetated pixels and excluded pixels with apparent mortality from before the target period. We also masked out pixels with high trait uncertainties (> 30%) based on pixel‐wise uncertainties (following estimation of uncertainty from Singh et al. (2015)). To avoid confusing fire‐related with drought‐related mortality, we masked out areas that had burned during the drought period (2012–2016) using fire boundaries from the California Department of Forestry and Fire Protection (CAL FIRE 2023). Finally, because both mortality datasets were developed specifically for trees, we applied a filter to include only pixels with at least 25% tree cover.

An initial comparison by Hemming‐Schroeder et al. (2023) of the two mortality datasets showed significant differences between the 2016 (Stovall et al. 2019) and 2017 (Hemming‐Schroeder et al. 2023) mortality estimates, especially in the Lower Teakettle site. We found that the areas of highest divergence between datasets can be explained by the use of National Agriculture Imagery Program imagery (NAIP; USDA 2003) in the Stovall et al. (2019) product. The mosaicked NAIP image from that year includes flightlines from one particular date that exhibit a high sensor zenith angle and, as a result, an oblique view of the target trees with extreme shading (Figure S5). Expecting the oblique view to distort reflectance and therefore mortality estimates (Myneni and Williams 1994), these areas were masked from the analysis for both mortality datasets.

### 2.3 Random Forest Modeling

We used random forest regressions to investigate the influence of the abiotic and biotic predictors on mortality outcome. Random forest is a machine learning algorithm that uses an ensemble of decision trees to predict an outcome (Breiman 2001a). In the case of random forest regressions, the output is the average prediction of the terminal node of individual trees. Random forests were chosen for their ability to incorporate large sample sizes, many predictors, nonlinear relationships between predictors and response, robustness to multicollinearity, and lack of assumptions regarding data distributions (Breiman 2001a, 2001b). Because of these strengths, random forests are increasingly used in ecological applications to investigate complex interactions in the natural world (Cutler et al. 2007; Prasad et al. 2006). In particular, they have been used to investigate the relationship between many predictors and tree mortality (Anderegg et al. 2016; Howe et al. 2022; Paz‐Kagan et al. 2017), and to assess high‐dimensionality relationships across functional traits (Russell et al. 2014).

After assembling the mortality covariates (Table S2), we assessed multicollinearity among predictors. Although random forests are robust to multicollinearity, the presence of highly correlated variables can confound interpretations of the model output (Molnar 2019). As such, in cases of two variables with a high correlation coefficient (> 0.7), only one variable was retained for analysis. Ultimately, most climate variables were highly correlated with other climate variables and with elevation in at least one site (Figure S4b). Because the climate models used to generate the climate inputs already include elevation (Thornton et al. 2021), we excluded these from analysis and kept elevation as representative of the climatic gradient. In addition, we expected correlation among some of the functional traits, since many are observed to be tightly linked across lifeforms (Díaz et al. 2016; Maynard et al. 2022). We found that the pair of structural compounds, lignin and cellulose, were highly correlated (Figure S4a). In consulting the correlations between these two traits and the other traits, cellulose generally was less correlated with the others than lignin was. As such, cellulose was retained. We also found that NSC, which is the sum of sugars and starch, was highly correlated with sugars (Figure S4a). Sugars and starch were retained separately while NSC was removed, as foliar sugars and starch (a) represent different components of NSC, (b) may show diverging responses to drought stress (Pflug et al. 2018), and (c) did not exceed the correlation coefficient threshold with other traits. Finally, the correlation between LMA and starch (0.7) was on the cusp of the threshold. Both traits hold ecological importance during drought: LMA links to leaf structure and ability to resist dry conditions (Wright et al. 2004), while leaf starch relates to storage and transport and shows sensitivity to drought (Hartmann and Trumbore 2016). Due to their distinct and important ecological roles, both were retained.

After selecting the final variables, we utilized 5‐fold cross validation to tune for hyperparameters using a grid search method with the Caret package in R (Kuhn 2008) and the Ranger implementation of random forests (Wright and Ziegler 2017). First, we modeled the two sites separately due to vast differences in species composition, topography, and extent of mortality. We then modeled each vegetation species class separately for all classes with at least 400 pixels (see section on “Species Composition” for description of classes). Because the assemblage of mixed species differs greatly between the two sites due to elevation differences, mixed conifer was modeled separately between sites. The final model for each site as a whole, then each species group, was selected using the lowest root mean square error (RMSE) across folds. The model runs were repeated once for the 2016 mortality dataset and once for the 2017 mortality dataset. *R* ^2^ and RMSE of the final models were recorded to assess performance. We generated variable importance for each model to assess how the foliar traits rank amongst the other drought predictors. Then we used the pdp package in R (Greenwell 2017) to generate partial dependence plots (PDPs), which show the variation in mortality across a given predictor gradient when other predictors are held constant (Friedman 2001).

Using 2013 vs. 2014 traits had minimal effect on model performance (Table S3). Because 2014 was the most severe year of the drought (USDA and NOAA 2023), analysis of the 2014 traits provided a unique opportunity to characterize plant functioning under extreme drought stress. As such, all results are shown using the 2014 traits.

## 3 Results

### 3.1 Site‐Level

Performance of the optimal models at the site level is shown in Table 2. Site and mortality data sources both caused variation in model results. A similar *R* ^2^ was reported across sites when predicting for 2016 mortality (3% difference), while *R* ^2^ varied widely across sites when predicting for 2017 mortality (24% difference). The highest overall *R* ^2^ was observed for the 2017 Soaproot model, while the lowest overall was observed for the 2017 Teakettle model. For both years, RMSE was lower for Teakettle.

**TABLE 2:** Performance metrics for site‐level models.

	Soaproot		Teakettle	
	*R* ^2^	RMSE	*R* ^2^	RMSE
2016 (Stovall et al.)	0.43	14.09	**0.46**	9.29
2017 (Hemming‐Schroeder et al.)	**0.55**	16.24	0.31	12.53

*Note:* Bold indicates highest *R* ^2^ for each site.

**FIGURE 2:**
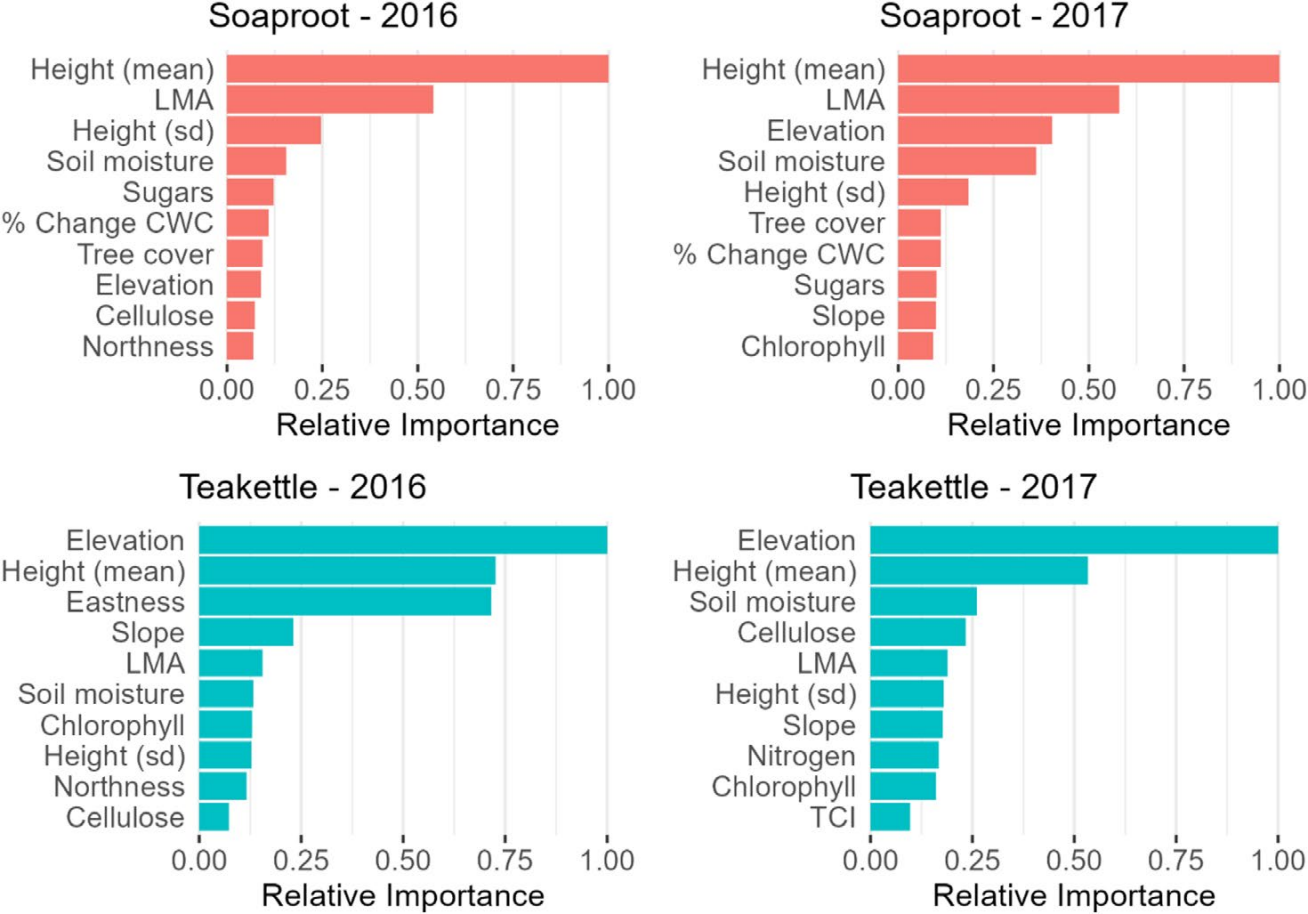
Variable importance is shown across sites using different mortality datasets. Only the top ten predictors are shown.

Variation across site and mortality year also affected variable importance. Though mean height emerged in the top three predictors across all models, remaining predictors varied. At Soaproot, the top three predictors for 2016 were mean height, LMA, and height SD (Figure 2). The top two predictors for 2017 were also mean height and LMA, along with elevation. At Teakettle, the top three predictors for 2016 were elevation, mean height, and topographic eastness (Figure 2). The top two predictors for 2017 were also elevation and mean height, while the third was soil moisture.

**FIGURE 3:**
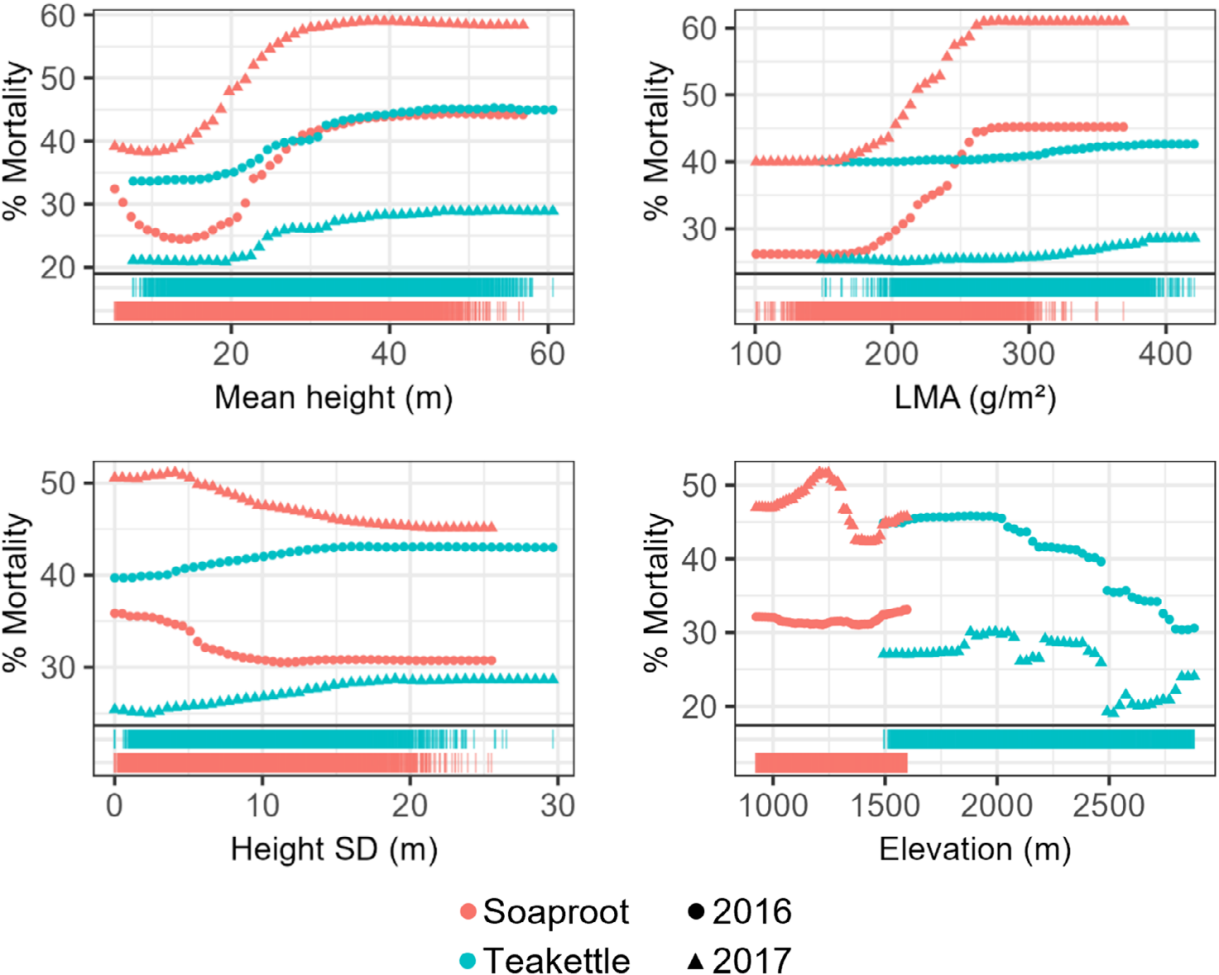
Select PDPs compare mortality trends for several of the top predictors of mortality across sites (symbol color). Rug plots show the distribution of mortality at each site across predictor gradients. Symbol shape denotes mortality dataset source (year).

PDPs showed similarities in the variation of mortality across abiotic and trait gradients for 2016 and 2017 predictions within each site (Figures 3 and S6). At Soaproot, percent mortality increased with increasing mean height, LMA, foliar sugars, and greater CWC loss; and decreased with increasing height SD, tree cover, soil moisture, and slope (Figures 3 and S6). The relationship with elevation varied between the models, with minimal variation in mortality in 2016, and higher mortality in 2017 at mid‐elevations (Figure 3). At Teakettle, percent mortality increased with increasing mean height, height SD, LMA, and topographic eastness; and decreased with increasing elevation and foliar nitrogen (Figures 3 and S6). Although results for Soaproot and Teakettle showed different directional trends for mortality across the elevation gradient, the actual elevation range of higher mortality for Soaproot and Teakettle was similar in 2016, with peaks around 1500–1600 m (Figure 3). These peaks diverged in 2017, with a Soaproot mortality peak at a lower range of 1200–1300 m, and a Teakettle mortality peak at a higher range of 1800–2100 m (Figure S6). Remaining covariates were ranked relatively low in variable importance and showed minimal influence on mortality across years and/or sites. These results are not discussed here but are included in the Supporting Information (Figure S6).

### 3.2 Species‐Level

Species‐specific models at Soaproot generally performed more consistently than Teakettle models, with a narrower range of *R* ^2^ values (Table 3). Models using 2017 mortality explained more variance than 2016 models for all species at Soaproot, with *R* ^2^ values 6%–11% higher. Conversely, models using 2016 mortality explained more variance than 2017 models for all species at Teakettle except mixed conifer, with *R* ^2^ values 10%–12% higher. Variable importance rankings revealed a wide array of mortality drivers for the different groups (Figure S7), and PDPs showed differing relationships across predictors (Figure S8). The highest model performance was observed for lodgepole pine, while the lowest performance was observed for white fir.

**TABLE 3:** Performance metrics for species‐level models.

Species	Site	Mortality year	*R* ^2^	RMSE
Canyon live oak	Soaproot	2016	0.42	12.32
		2017	**0.50**	13.50
Chaparral	Soaproot	2016	0.34	13.96
		2017	**0.45**	14.24
Coast live oak	Soaproot	2016	0.44	10.38
		2017	**0.50**	10.63
Ponderosa pine	Soaproot	2016	0.42	14.23
		2017	**0.49**	15.96
Soaproot mixed conifer	Soaproot	2016	0.37	13.95
		2017	**0.47**	16.83
Teakettle mixed conifer	Teakettle	2016	0.38	10.87
		2017	**0.49**	11.99
Lodgepole pine	Teakettle	2016	**0.57**	7.57
		2017	0.45	9.26
Red fir	Teakettle	2016	**0.41**	8.86
		2017	0.31	11.78
White fir	Teakettle	2016	**0.31**	9.42
		2017	0.21	13.17

*Note:* Bold indicates highest *R* ^2^ for each species.

#### 3.2.1 Broadleaf Trees and Shrubs

**FIGURE 4:**
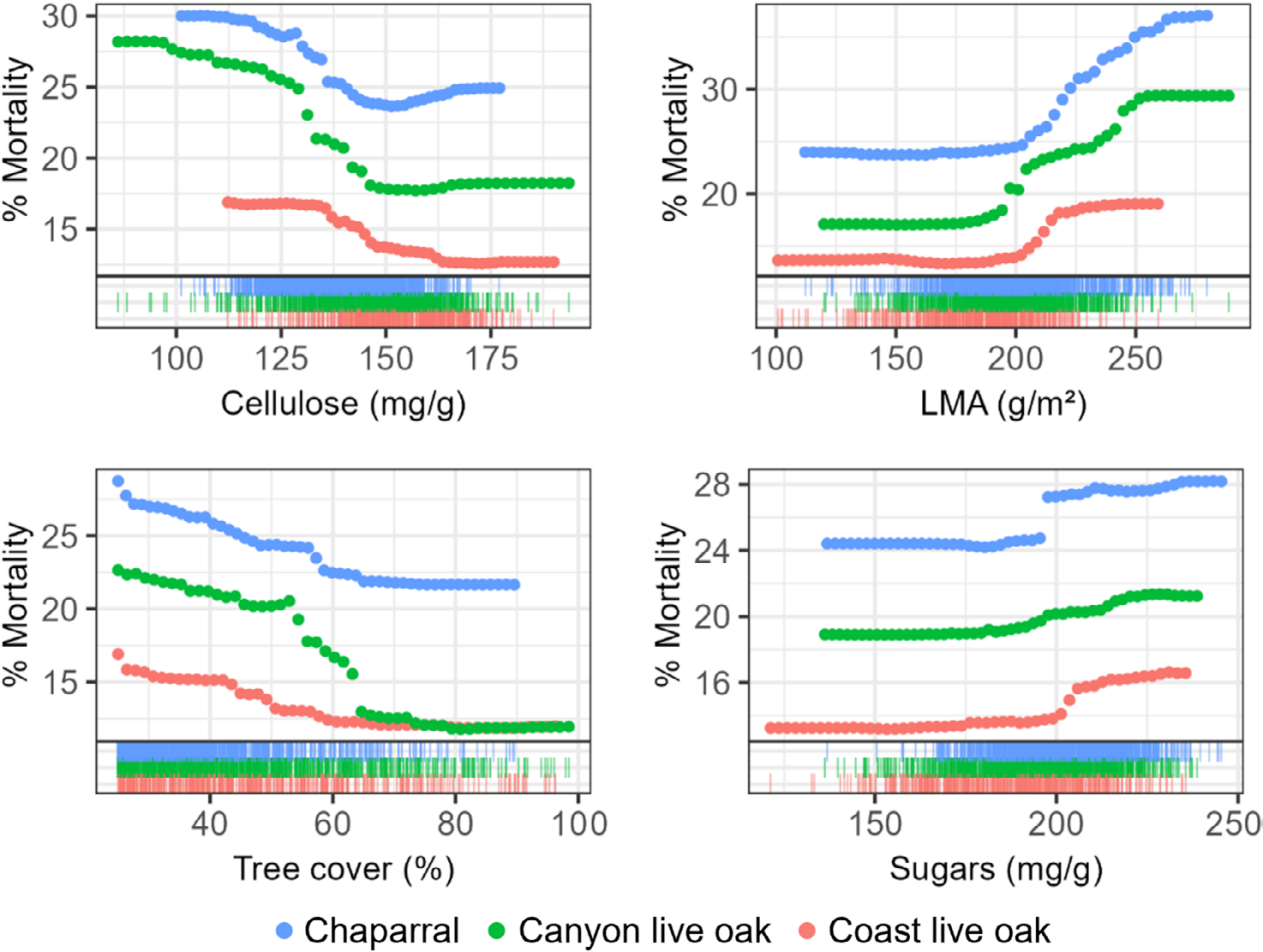
Select PDPs compare 2016 mortality trends for several of the top predictors of mortality across broadleaf and shrub species (symbol color). Rug plots show the distribution of each species across predictor gradients.

Model performance was higher for the two oaks than for chaparral, though percent mortality was generally low across all groups (Table 3, Figure 4). The three species groups had considerable overlap between top predictors (Figure S7), but the year of mortality had a clear effect. For 2016 mortality, LMA, mean height, and cellulose were among the main drivers across all species. In contrast, the top predictors for 2017 mortality had few variables in common across the species, with sugars, LMA, and slope as the most important for canyon live oak, chaparral, and coast live oak, respectively.

Despite differences in importance rankings, PDPs showed similarities in the variation of mortality across abiotic and trait gradients for 2016 and 2017 predictions (Figures 4 and S8). Across all three species groups, mortality increased with increasing LMA, leaf sugars, and height SD, and decreased with increasing leaf cellulose and tree cover (Figures 4 and S8). The influence of mean height was mixed; mortality generally increased with height for chaparral and canyon live oak, and decreased with height for coast live oak (Figure S8). In addition, coast live oak displayed higher mortality on less steep slopes, though slope had minimal effect on the other species groups (Figure S8). Finally, elevation had a variable mortality response across years, with a stronger effect on all species groups in 2017 models. In this case, mortality increased with increasing elevation for chaparral and canyon live oak, and decreased with increasing elevation for coast live oak (Figure S8). Remaining covariates were ranked relatively low in variable importance and showed minimal influence on mortality across years and/or broadleaf tree and shrub species groups. These results are not discussed here but are included in the Supporting Information (Figure S8).

#### 3.2.2 Soaproot Conifers

Model performance for ponderosa pine and Soaproot mixed conifer was similar across mortality years, although 2017 predictions yielded consistently higher *R* ^2^ values (as well as higher error) than 2016 predictions (Table 3). The two groups had a high degree of overlap in top predictors, with mean height emerging as especially important to both (Figure S7). LMA and elevation were also important for both groups, but their relative importance differed: LMA was a more important predictor for ponderosa pine, while elevation was a more important predictor for mixed conifer. In addition, height SD was of higher importance for ponderosa pine than for mixed conifer.

**FIGURE 5:**
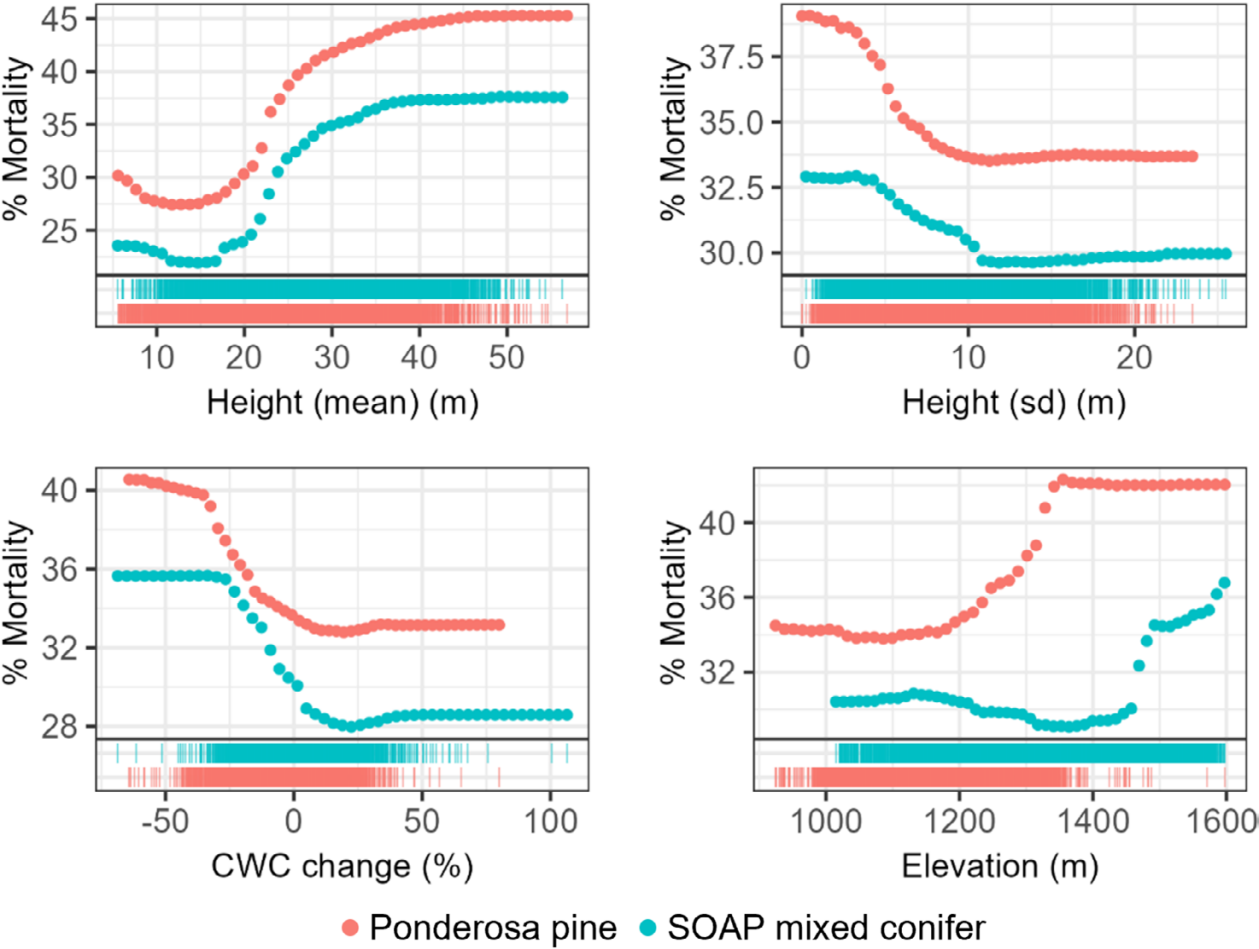
Select PDPs compare 2016 mortality trends for several of the top predictors of mortality across Soaproot conifer species (symbol color). Rug plots show the distribution of each species across predictor gradients.

Though relative rankings differed, PDPs for both species groups showed increasing mortality with increasing mean height, LMA, leaf sugars, and CWC loss; and decreasing mortality with increasing height SD, tree cover, foliar nitrogen, soil moisture, slope, topographic northness, and TCI (Figures 5 and S8). Finally, both groups generally displayed higher mortality at the higher end of their elevation range (> 1200 m for ponderosa pine, > 1500 m for mixed conifer), although the 2017 model for mixed conifer showed an additional peak at the lower end of its range (~1100 m) (Figures 5 and S8). Remaining covariates were ranked relatively low in variable importance and showed minimal influence on mortality across years and/or Soaproot conifer species groups. These results are not discussed here but are included in the Supporting Information (Figure S8).

#### 3.2.3 Teakettle Conifers

Model performance varied widely across species and mortality years for conifers at Teakettle. The 2016 predictions yielded higher *R* ^2^ values with lower error for all species groups except Teakettle mixed conifer (Table 3). Model performance for the fir‐dominated forests was generally poor, with the three poorest performing models belonging to the white and red fir classes. The species groups had widely variable top predictors, though elevation emerged as the most important across all groups (Figure S7).

**FIGURE 6:**
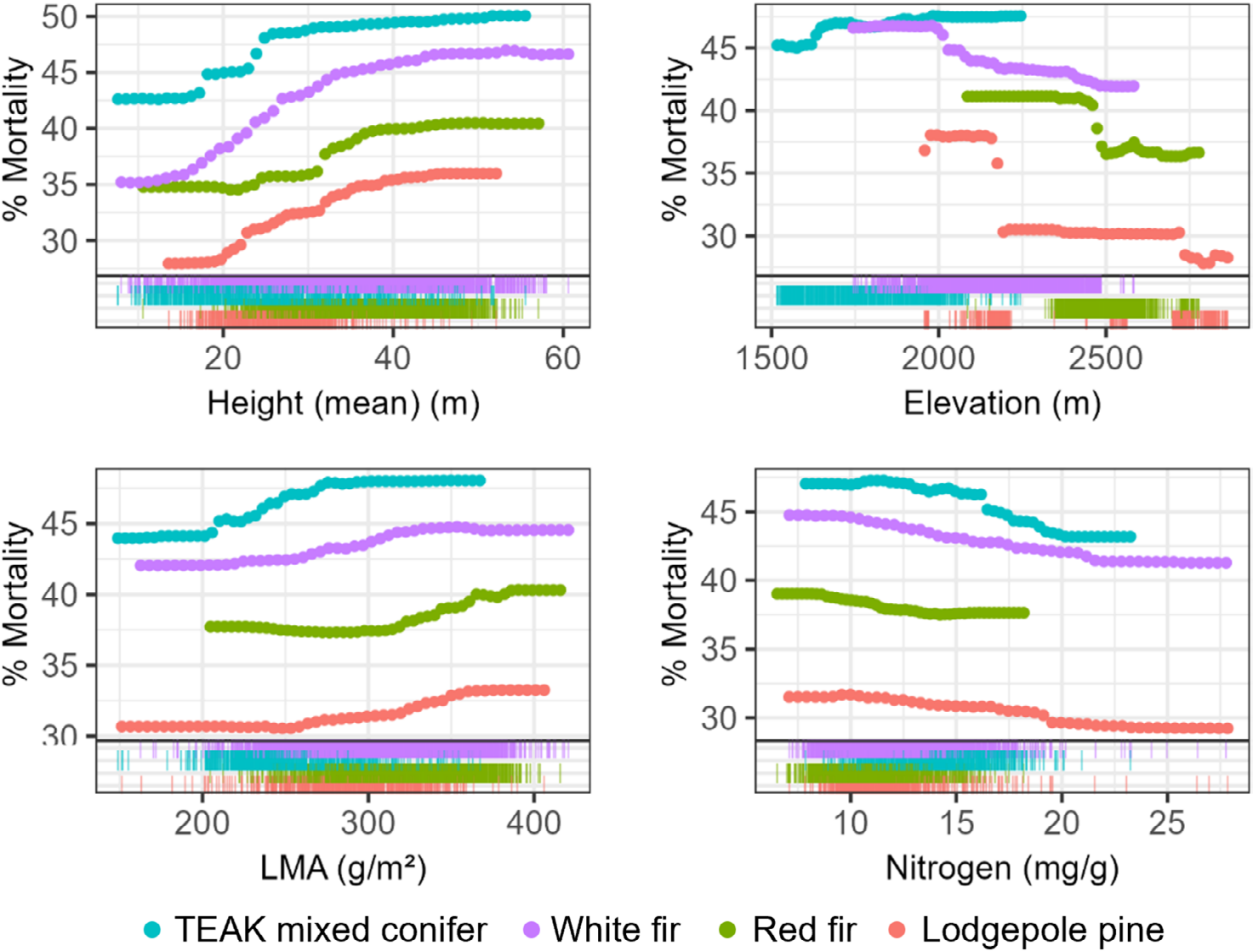
Select PDPs compare 2016 mortality trends for several of the top predictors of mortality across Teakettle conifer species (symbol color). Rug plots show the distribution of each species across predictor gradients.

While importance rankings varied widely, PDPs for all species groups showed higher mortality for taller trees and increasing height SD, LMA, and topographic eastness; and decreasing mortality with increasing leaf nitrogen (Figures 6 and S8). Mortality was generally higher on drier soil, though red fir showed a mortality peak at slightly higher soil moisture (Figure S8). Though elevation was important for all groups, mortality variation across the elevation gradient differed across species (Figure 6). Mortality was higher at lower elevations for red fir (< 2400 m) and higher elevations for mixed conifer (> 1900 m), but had a variable response across mortality years for lodgepole pine and white fir. In both cases, mortality was higher at lower elevations in 2016 (< 2200 and < 2000 m, respectively), and patchier (with multiple peaks) in 2017 (Figures 6 and S8). Finally, across groups, leaf chlorophyll showed a stronger influence on mortality for red fir, with higher mortality associated with lower chlorophyll (Figure S8). Remaining covariates were ranked relatively low in variable importance and showed minimal influence on mortality across years and/or Teakettle conifer species groups. These results are not discussed here but are included in the Supporting Information (Figure S8).

## 4 Discussion

### 4.1 Site‐Level Mortality

Top drivers of mortality differed by site, with a stronger foliar trait effect at the lower elevation Soaproot site. Mortality at Soaproot was best predicted by mean height and LMA, suggesting contributions from the two major axes of tree trait variation, plant size and leaf economics (Díaz et al. 2016; Maynard et al. 2022). Other top predictors for Soaproot, soil moisture and height SD, indicate the importance of water availability and a possible influence of size‐related beetle host preference. In contrast, Teakettle mortality was explained primarily by elevation and mean height. PDPs showed higher mortality at the lower elevation range in Teakettle, which would fall more in the water‐limited area of this site. Lower than normal precipitation was also linked to lower elevation at this site (Figure S4b), illustrating the influence of climatic conditions at higher elevations, where higher precipitation and deeper snowpack can buffer tree susceptibility to embolism. Despite changes in importance rankings across mortality years, the consistency of the top two predictors for both sites indicates general stability in mortality patterns from 2016–2017 at the site level.

The difference between main drivers at the two sites likely stems from broad variation in climatic and topographic characteristics, as well as species composition. Teakettle, at higher elevation, rests in the water‐ to energy‐limited forest transition zone (Das et al. 2013). This means the forests there experience less water stress in normal years and are expected to display lower adaptation to severe drought (Bréda et al. 2006). The two sites are also dominated by different species, which have specific adaptations to water limitations, exhibit unique insect species associations, and have already been observed to have variable mortality rates (Restaino et al. 2019; Stephenson et al. 2019).

### 4.2 Species‐Level Mortality

#### 4.2.1 Broadleaf Trees and Shrubs

In the case of the broadleaf trees and shrubs, survival appears to be linked to drought‐adaptive foliar traits. For all species, higher leaf cellulose was related to lower mortality. Sierra oak species and chaparral are known for sclerophyllous traits, which include higher cellulose concentration, contributing to a leaf that is stronger and more resistant to stressful conditions (Alonso‐Forn et al. 2023). Stronger leaves are more resistant to collapse (caused by negative turgor pressure experienced under extreme drought) and contribute to the drought tolerance of the tree (Nardini 2022). In turn, foliar traits contributing to drought tolerance have been shown to buffer crown dieback (Marchin et al. 2022).

The oaks and chaparral also exhibited higher mortality with higher LMA. Although higher cellulose increases leaf density (a component of LMA), cellulose and LMA showed opposite patterns with mortality. This could indicate a stronger contribution of leaf thickness (rather than density) to LMA (Sancho‐Knapik et al. 2021), and suggests that a denser leaf contributed more to survival than a thicker leaf (this is further supported by the low correlation between LMA and cellulose, Figure S4a). Lower LMA may be an adaptive trait in this case, as seen in another drought‐resistant oak species (Ogaya and Penuelas 2006). Because LMA is tightly linked to photosynthetic capacity (Poorter et al. 2009), it is possible that leaves with lower leaf thickness and lower LMA were better suited to maximize photosynthesis early in the drought to provide more carbon stores for times of stress.

Higher leaf sugars were also associated with higher mortality, although leaf starch showed little effect. During drought stress, it is possible for the plant to accumulate NSCs in the leaf due to the decoupling of growth and photosynthesis, utilization for osmoregulation, or disruption in phloem transport (McDowell 2011). Such accumulation of foliar sugars in response to drought has been observed in both broadleaf and conifer species (Adams et al. 2013; Mitchell et al. 2014), and theoretically would occur before carbon starvation (McDowell et al. 2008). If the higher NSCs are a symptom of severe drought stress, it follows that the most stressed trees (with higher NSC accumulation) experienced higher mortality rates.

Finally, broadleaf and shrub mortality was consistently related to stand structure. Lower tree cover was linked to higher mortality across all broadleaf and shrub species and showed the highest relative importance for the broadleaf and shrub species compared to the other groups. Higher tree cover likely contributed to a milder microclimate, easing drought stress on the trees by reducing evaporative water loss (Davis et al. 2019; Rambo and North 2009). This may also relate to the increasing mortality observed for increasing height SD. Since the microclimate effect of an isolated oak canopy is highly localized (Parker and Muller 1982), a stand with some individuals of lower stature may suffer higher climatic stress through the existence of more sunlit gaps.

#### 4.2.2 Soaproot Conifers

For conifers at Soaproot Saddle, mortality appears to be linked to severe water stress and insect preferences. We observed that higher mortality for both ponderosa pine and Soaproot mixed conifer was linked to higher CWC losses. Decreased CWC indicates that the canopy was already severely water stressed (Brodrick and Asner 2017; McDowell et al. 2008; Paz‐Kagan et al. 2017), which was further supported by the lower soil moisture observed for higher mortality areas (Goulden and Bales 2019). As such, these trees likely experienced some degree of hydraulic failure—which likely drove further mortality via beetle attack. Under high physiological stress, bark beetles target larger trees of ponderosa pine (as well as sugar pine, which occurs in the mixed conifer stands) (Koontz et al. 2021; Stephenson et al. 2019), due to thicker phloem gains and lowered defenses (Kolb et al. 2007). The higher mortality observed for taller, same‐sized stands (indicated by mean height and height SD) further indicates the role of species‐specific, size‐driven preferences for ponderosa pine. Ultimately, the influences of hydraulic stress and insect outbreaks are tightly linked and cannot be completely disentangled. Though trees undergo structural and physiological adaptations that help overcome the effects of increased hydraulic resistance as they grow taller, it is poorly understood whether these adjustments can outlast the effects of longer, more severe droughts (Fernández‐de‐Uña et al. 2023).

As observed for the broadleaf species, higher mortality in the Soaproot conifers was also associated with higher LMA. In this case, it could be that higher LMA reflects leaf characteristics in taller or older trees (Poorter et al. 2009). Alternatively, lower LMA in surviving trees could indicate a survival advantage from higher photosynthetic capacity early in the drought, as described for the broadleaf classes. This is further supported by the observed lower mortality associated with higher leaf nitrogen and chlorophyll, both of which are linked to higher photosynthetic capacity (Croft et al. 2017; Kattge et al. 2009).

#### 4.2.3 Teakettle Conifers

The Teakettle conifers occur at the transition zone from water‐ to energy‐limited forest (Das et al. 2013), which means the drought tolerance of the higher elevation species may be lower than that of Soaproot due to the cooler and wetter conditions that occur in their range (Bréda et al. 2006). While mortality increased with mean tree height for all groups, it had a relatively weak effect in conifers at Teakettle compared to those at Soaproot. This matches previous findings that height‐related mortality occurred for all species groups in this region (Stovall et al. 2020), but may have been stronger in Soaproot's dominant species (e.g., *Pinus ponderosa, P. lambertiana*) than Teakettle's dominant species (e.g., *Abies concolor, A* *. magnifica* ) (Stephenson and Das 2020). More so than height, mortality in the Teakettle species was linked to elevation. Due to the high correlation between elevation, precipitation anomaly, and mean temperature anomaly (Figure S4b), this suggests that mortality was driven by the climatic elements of the drought. Though a link to climate appears strong, evidence of a mortality mechanism supported by functional traits is lacking. Due to lower drought adaptation, the signs of early drought stress may not manifest in the same way we observed with the Soaproot trees, including decreased CWC and foliar accumulation of NSCs. Alternatively, it is possible that signs of severe drought stress were not apparent as early as 2014.

Ultimately, the lower *R* ^2^ values for red and white fir suggest that an unexplained driver/mechanism for mortality remains, which was also observed in a previous study of mortality drivers in energy‐limited forests (Das et al. 2013). Field observations showed that white fir of all sizes died during the drought (Stephenson et al. 2019), which was attributed to variable size preferences of three different beetle species that attack white fir—in our study, that would make it a particularly difficult tree species to predict mortality for without more detailed beetle data. Of note, leaf chlorophyll appeared relatively more important as a predictor for red fir mortality than any other species, with higher mortality observed where leaf chlorophyll was initially lower. The importance of leaf chlorophyll for this particular species matches a previous result that showed higher chlorophyll stress in red fir in a period of moderate to severe drought (Swatantran et al. 2011; USDA and NOAA 2023), suggesting that chlorophyll may be a consistent predictor of stress for this species. Finally, low performance for these species may relate to the spatial resolution of our study (some fine‐scale processes driving mortality may be impossible to detect at the 30 m level), or the failure to capture other important drivers, such as bedrock composition (Callahan et al. 2022).

### 4.3 Mortality Data

Site‐level results showed similar trends in mortality across predictor gradients for both mortality datasets, indicating that both can be used to consistently capture broad‐scale mortality trends. However, we observed a noticeable difference in 2016 and 2017 mortality across the elevation gradient. The stronger influence of elevation in 2017 (and climate anomalies by proxy) could result from the lagged mortality of trees ultimately dying in 2017 (rather than 2016). Lag effects of drought on tree mortality have been observed in other studies and are species‐dependent (Bigler et al. 2007; Férriz et al. 2021), which may explain why differences in variable importance and PDPs across mortality years were more apparent for some species. We also observed a disparity in model performance from 2016 to 2017, particularly at Teakettle. The underestimation of 2017 mortality at Teakettle from Hemming‐Schroeder et al. relative to the 2016 estimation from Stovall et al. is discussed at length by the former (Hemming‐Schroeder et al. 2023), likely owing to differences in preprocessing steps for reflectance data and errors in co‐registration of data products across the complex terrain of the Sierra Nevada. These differences may affect our interpretation of results for Teakettle mortality.

### 4.4 Species Data

Functional trait gradients can be interpreted on their own (Díaz et al. 2016), but the addition of species information is especially useful in a case where mortality has been shown to vary greatly by species, resulting from drought adaptive strategies or unique insect/pathogen relationships (Restaino et al. 2019; Stephenson et al. 2019). The species map used for this study allowed stratification by dominant species groups, and results align with other results for these species in the literature (Stephenson and Das 2020; Stovall et al. 2019, 2020). However, we recognize that the forest type map may be outdated and provides a coarse view of species distribution. Although species mapping techniques are becoming more advanced and availability is increasing (Marconi et al. 2022), highly detailed species information still remains limited by temporal availability and spatial extent. A more accurate species map would help disentangle mortality–predictor relationships in mixed pixels.

### 4.5 Drought Monitoring for the Future

Here we analyze a suite of foliar traits that are measurable across broad landscapes from imaging spectroscopy that can provide insights into multi‐year forest response to drought. Across different species assemblages, we identified clear links between certain foliar traits during a time of drought stress and subsequent mortality outcomes. This work is enabled by the availability of hyperspectral remote sensing imagery, which until recently was limited in spatial and temporal extent. Although our study did not encompass the full scope of pre‐ and post‐drought trait variation, such insights would have been challenging to acquire with an exclusively in situ approach, as that would have required knowledge of oncoming drought and extensive resources to sample the spatial and temporal extent of this study. However, current and forthcoming spaceborne hyperspectral sensors like the Earth Surface Mineral Dust Source Investigation (EMIT; Green et al. 2020), the Environmental Mapping and Analysis Program (EnMAP; Stuffler et al. 2007), the Hyperspectral Precursor of the Application Mission (PRISMA; Loizzo et al. 2018), the Hyperspectral Imager Suite (HISUI; Iwasaki et al. 2011), the Copernicus Hyperspectral Imaging Mission for the Environment (CHIME; Nieke and Rast 2018), and Surface Biology and Geology (SBG; Cawse‐Nicholson et al. 2021) will greatly expand the extent of available hyperspectral data, ultimately resulting in a global hyperspectral time series that will enable foliar trait characterization on unprecedented scales and across a wide range of environmental perturbations. As droughts become more frequent and severe (Dai 2013; Diffenbaugh et al. 2015; Wahl et al. 2022), plant trait variation will be measurable leading up to, during, and after future droughts. This opportunistic study provides a starting point to work towards identifying early indicators of drought‐related mortality risk, while future data may shed new insights on how these traits vary across ecosystems and biomes in response to drought. These insights can be used as a basis for hypothesis testing in future studies and for the design of in situ sampling to support the image‐derived interpretations.

## 5 Conclusion

Here, we showed that two independently derived mortality datasets can be used to investigate mortality trends across gradients of foliar trait variation, derived from regional‐scale imaging spectroscopy. Incorporating traits along with known important predictors of mortality in the area illustrates a highly variable response at two diverse sites. For the lower elevation, more water‐limited site, mortality is strongly linked to tree height, decreased CWC, LMA, leaf cellulose, and leaf sugars, with variation across conifer and broadleaf forest types. The importance of tree height and decreased CWC in conifers shows evidence of some level of hydraulic failure as well as insect‐host preference, while the importance of foliar traits for broadleaf and shrub shows evidence of leaf‐level drought adaptation. For the higher elevation, more energy‐limited site, mortality is strongly linked to climate and topography, with less influence from functional traits. This suggests that severe drought conditions are related to mortality, though the actual mortality mechanism (e.g., hydraulic failure, carbon starvation, or insect host preference) is less clear. Hyperspectral imagery is crucial to this study, and its availability is rapidly expanding across broader spatial and temporal scales with current and upcoming spaceborne missions as well as increased availability of airborne imaging spectroscopy. Foliar trait maps derived from imaging spectroscopy will enable further characterization of physiological drought response across species, ecosystems, and biomes, as we continue to unravel the complex responses of vegetation to global change.

## Author Contributions

**Natalie Queally:** conceptualization, data curation, formal analysis, funding acquisition, investigation, methodology, visualization, writing – original draft, writing – review and editing. **Ting Zheng:** data curation, investigation, methodology, writing – review and editing. **Zhiwei Ye:** data curation, methodology, writing – review and editing. **Kyle R. Kovach:** investigation, methodology, writing – review and editing. **Ryan Pavlick:** conceptualization, data curation, writing – review and editing. **Ethan Shafron:** data curation, writing – review and editing. **Fabian D. Schneider:** conceptualization, data curation, writing – review and editing. **Philip A. Townsend:** conceptualization, data curation, funding acquisition, methodology, resources, supervision, writing – original draft, writing – review and editing.

## Conflicts of Interest

The authors declare no conflicts of interest.

## Supporting information


**Figure S1.** Trait mapping workflow used for this study. Blue boxes indicate data inputs, white boxes indicate processing steps, orange boxes indicate intermediate data products, and the green box indicates the final trait map product. The gray box describes the PLSR model training/testing workflow in greater detail. Major image processing steps include: topographic and BRDF corrections (applied using FlexBRDF; Queally et al., 2022) to reduce the influence of unwanted brightness gradients from terrain and solar/sensor geometries on trait estimates; and alignment to the Landsat grid (using AROSICS; Scheffler et al. 2017) to improve year‐to‐year comparisons across pixels.
Figure S1 References
**Figure S2.** The seasonal trend in MODIS NDVI shows that 2013 and 2014 image acquisitions (2013‐06‐12 and 2014‐06‐03, respectively) occurred during similar phenological periods. At Soaproot, the acquisitions occur just after a greenness peak (though note the corresponding peak is relatively lower in 2014 as compared to 2013). At Teakettle, the acquisitions occur leading up to peak greenness.
**Figure S3.** Mean model predictions across 500 model permutations shown in blue, with standard deviation indicated by horizontal bars. 1:1 line shown in black.
**Figure S4.** We observed high correlation amongst some traits (a), and amongst some climate variables and elevation (b), with variation across the two sites.
**Figure S5.** Influence of high sensor zenith angle on NAIP imagery in Stovall et al. (2019) study at Teakettle. Large differences between the 2017 (Hemming‐Schroeder et al.) and 2016 (Stovall) mortality data show patchiness (a). These same patches are evident in the 2016 mortality data alone, and align precisely with flight line boundaries across dates (b). Imagery from the June 30 acquisition show high sensor zenith angle, and resulting oblique view of forest canopy (c). Areas outlined in pink (6/30/16 acquisition) were masked at the Teakettle site, with 52.4% of pixels retained.
**Figure S6.** Site level partial dependence plots show mortality trend across all predictor gradients. Accompanying rug plots show site‐specific data distributions. Results are shown separately for models using 2016 and 2017 mortality. General trends are similar for top predictors (e.g., increasing mortality with increasing mean tree height), though magnitude of mortality varies across mortality data source.
**Figure S7.** Variable importance of species‐level models using 2016 mortality data (left) and 2017 mortality data (right).
**Figure S8.** Species level partial dependence plots show mortality trend across all predictor gradients. Accompanying rug plots show species‐specific data distributions. Results are shown separately for models using 2016 and 2017 mortality.
**Table S1.** Trait model performance for trait maps used in this study, with mean *R* ^2^ and normalized RMSE across 500 PLSR model permutations.
**Table S2.** Covariates assembled for drought mortality analysis. Italics denote variable was not used for final analysis.
**Table S3.** Comparison of model performance using trait maps derived from 2013 and 2014 AVIRIS‐C imagery. Trait year had minimal effect on model performance.

## Data Availability

The data and code that support the findings of this study are openly available in Zenodo at https://doi.org/10.5281/zenodo.13436293. Tree mortality data were obtained from figshare at https://doi.org/10.6084/M9.FIGSHARE.7609193.V1 and Zenodo at https://doi.org/10.5281/ZENODO.7812035. Canopy water content data were obtained from PANGAEA at https://doi.org/10.1594/PANGAEA.897276. Climate data from Daymet were accessed at https://doi.org/10.3334/ORNLDAAC/2131 and from WorldClim2 at https://doi.org/10.1002/joc.5086. The Shuttle Radar Topography Mission (SRTM) digital elevation model was accessed from USGS EarthExplorer. Soil moisture data from SMAP‐HydroBlocks were accessed from Zenodo at https://doi.org/10.5281/zenodo.5206725.

## References

[gcb70246-bib-0001] Adams, H. D. , M. J. Germino , D. D. Breshears , et al. 2013. “Nonstructural Leaf Carbohydrate Dynamics of *Pinus edulis* During Drought‐Induced Tree Mortality Reveal Role for Carbon Metabolism in Mortality Mechanism.” New Phytologist 197, no. 4: 1142–1151. 10.1111/nph.12102. 23311898

[gcb70246-bib-0002] Adams, H. D. , M. J. B. Zeppel , W. R. L. Anderegg , et al. 2017. “A Multi‐Species Synthesis of Physiological Mechanisms in Drought‐Induced Tree Mortality.” Nature Ecology & Evolution 1, no. 9: 1285–1291. 10.1038/s41559-017-0248-x. 29046541

[gcb70246-bib-0003] AghaKouchak, A. , L. Cheng , O. Mazdiyasni , and A. Farahmand . 2014. “Global Warming and Changes in Risk of Concurrent Climate Extremes: Insights From the 2014 California Drought: Global Warming and Concurrent Extremes.” Geophysical Research Letters 41, no. 24: 8847–8852. 10.1002/2014GL062308.

[gcb70246-bib-0004] Alonso‐Forn, D. , D. Sancho‐Knapik , M. D. Fariñas , et al. 2023. “Disentangling Leaf Structural and Material Properties in Relationship to Their Anatomical and Chemical Compositional Traits in Oaks (*Quercus* L.).” Annals of Botany 131, no. 5: 789–800. 10.1093/aob/mcad030. 36794926 PMC10184456

[gcb70246-bib-0005] Anderegg, W. R. L. , T. Klein , M. Bartlett , et al. 2016. “Meta‐Analysis Reveals That Hydraulic Traits Explain Cross‐Species Patterns of Drought‐Induced Tree Mortality Across the Globe.” Proceedings of the National Academy of Sciences 113, no. 18: 5024–5029. 10.1073/pnas.1525678113. PMC4983847 27091965

[gcb70246-bib-0006] Asner, G. P. , P. G. Brodrick , C. B. Anderson , N. Vaughn , D. E. Knapp , and R. E. Martin . 2016. “Progressive Forest Canopy Water Loss During the 2012–2015 California Drought.” Proceedings of the National Academy of Sciences 113, no. 2: 7E249–E255. 10.1073/pnas.1523397113 PMC4720336 26712020

[gcb70246-bib-0007] Asner, G. P. , R. E. Martin , C. B. Anderson , and D. E. Knapp . 2015. “Quantifying Forest Canopy Traits: Imaging Spectroscopy Versus Field Survey.” Remote Sensing of Environment 158: 15–27. 10.1016/j.rse.2014.11.011.

[gcb70246-bib-0008] Bader, M. Y. , and J. J. A. Ruijten . 2008. “A Topography‐Based Model of Forest Cover at the Alpine Tree Line in the Tropical Andes.” Journal of Biogeography 35, no. 4: 711–723. 10.1111/j.1365-2699.2007.01818.x.

[gcb70246-bib-0009] Berg, N. H. 1990. Experimental Forests and Ranges: Field Research Facilities of the Pacific Southwest Research Station (Vol. 119). Station.

[gcb70246-bib-0010] Bigler, C. , D. G. Gavin , C. Gunning , and T. T. Veblen . 2007. “Drought Induces Lagged Tree Mortality in a Subalpine Forest in the Rocky Mountains.” Oikos 116, no. 12: 1983–1994. 10.1111/j.2007.0030-1299.16034.x.

[gcb70246-bib-0011] Bréda, N. , R. Huc , A. Granier , and E. Dreyer . 2006. “Temperate Forest Trees and Stands Under Severe Drought: A Review of Ecophysiological Responses, Adaptation Processes and Long‐Term Consequences.” Annals of Forest Science 63, no. 6: 625–644. 10.1051/forest:2006042.

[gcb70246-bib-0012] Breiman, L. 2001a. “Random Forests.” Machine Learning 45, no. 1: 5–32. 10.1023/A:1010933404324.

[gcb70246-bib-0013] Breiman, L. 2001b. “Statistical Modeling: The Two Cultures (With Comments and a Rejoinder by the Author).” Statistical Science 16, no. 3: 199–231. 10.1214/ss/1009213726.

[gcb70246-bib-0014] Brodrick, P. G. , L. D. L. Anderegg , and G. P. Asner . 2019. “Forest Drought Resistance at Large Geographic Scales.” Geophysical Research Letters 46, no. 5: 2752–2760. 10.1029/2018GL081108.

[gcb70246-bib-0015] Brodrick, P. G. , and G. P. Asner . 2017. “Remotely Sensed Predictors of Conifer Tree Mortality During Severe Drought.” Environmental Research Letters 12, no. 11: 115013. 10.1088/1748-9326/aa8f55.

[gcb70246-bib-0016] CAL FIRE , US Forest Service , Bureau of Land Management , and National Park Service . 2023. Fire Perimeters. CAL FIRE FRAP (Fire and Resource Assessment Program). https://frap.fire.ca.gov/frap‐projects/fire‐perimeters/.

[gcb70246-bib-0017] Callahan, R. P. , C. S. Riebe , L. S. Sklar , et al. 2022. “Forest Vulnerability to Drought Controlled by Bedrock Composition.” Nature Geoscience 15, no. 9: 714–719. 10.1038/s41561-022-01012-2.

[gcb70246-bib-0018] Carter, G. A. , and A. K. Knapp . 2001. “Leaf Optical Properties in Higher Plants: Linking Spectral Characteristics to Stress and Chlorophyll Concentration.” American Journal of Botany 88, no. 4: 677–684. 11302854

[gcb70246-bib-0019] Cawse‐Nicholson, K. , P. A. Townsend , D. Schimel , et al. 2021. “NASA'S Surface Biology and Geology Designated Observable: A Perspective on Surface Imaging Algorithms.” Remote Sensing of Environment 257: 112349. 10.1016/j.rse.2021.112349.

[gcb70246-bib-0020] Chaves, M. M. , J. S. Pereira , J. Maroco , et al. 2002. “How Plants Cope With Water Stress in the Field? Photosynthesis and Growth.” Annals of Botany 89, no. 7: 907–916. 10.1093/aob/mcf105. 12102516 PMC4233809

[gcb70246-bib-0021] Cherif, E. , H. Feilhauer , K. Berger , et al. 2023. “From Spectra to Plant Functional Traits: Transferable Multi‐Trait Models From Heterogeneous and Sparse Data.” Remote Sensing of Environment 292: 113580.

[gcb70246-bib-0022] Crockett, J. L. , and A. L. Westerling . 2018. “Greater Temperature and Precipitation Extremes Intensify Western U.S. Droughts, Wildfire Severity, and Sierra Nevada Tree Mortality.” Journal of Climate 31, no. 1: 341–354. 10.1175/JCLI-D-17-0254.1.

[gcb70246-bib-0023] Croft, H. , J. M. Chen , X. Luo , P. Bartlett , B. Chen , and R. M. Staebler . 2017. “Leaf Chlorophyll Content as a Proxy for Leaf Photosynthetic Capacity.” Global Change Biology 23, no. 9: 3513–3524. 10.1111/gcb.13599. 27976452

[gcb70246-bib-0024] Cutler, D. R. , T. C. Edwards , K. H. Beard , et al. 2007. “Random Forests for Classification in Ecology.” Ecology 88, no. 11: 2783–2792. 10.1890/07-0539.1. 18051647

[gcb70246-bib-0025] Dai, A. 2013. “Increasing Drought Under Global Warming in Observations and Models.” Nature Climate Change 3, no. 1: 52–58. 10.1038/nclimate1633.

[gcb70246-bib-0026] Das, A. J. , M. R. Slaton , J. Mallory , G. P. Asner , R. E. Martin , and P. Hardwick . 2022. “Empirically Validated Drought Vulnerability Mapping in the Mixed Conifer Forests of the Sierra Nevada.” Ecological Applications 32, no. 2: e2514. 10.1002/eap.2514. 35094444

[gcb70246-bib-0027] Das, A. J. , N. L. Stephenson , A. Flint , T. Das , and P. J. Van Mantgem . 2013. “Climatic Correlates of Tree Mortality in Water‐ and Energy‐Limited Forests.” PLoS One 8, no. 7: e69917. 10.1371/journal.pone.0069917. 23936118 PMC3723662

[gcb70246-bib-0028] Davis, F. W. , N. W. Synes , G. A. Fricker , et al. 2019. “LiDAR‐Derived Topography and Forest Structure Predict Fine‐Scale Variation in Daily Surface Temperatures in Oak Savanna and Conifer Forest Landscapes.” Agricultural and Forest Meteorology 269: 192–202. 10.1016/j.agrformet.2019.02.015.

[gcb70246-bib-0116] De Bello, F. , S. Lavorel , S. Díaz , et al. 2010. “Towards an Assessment of Multiple Ecosystem Processes and Services via Functional Traits.” Biodiversity and Conservation 19: 2873–2893. 10.1007/s10531-010-9850-9.

[gcb70246-bib-0029] Díaz, S. , J. Kattge , J. H. C. Cornelissen , et al. 2016. “The Global Spectrum of Plant Form and Function.” Nature 529, no. 7585: 167–171. 10.1038/nature16489. 26700811

[gcb70246-bib-0030] Diffenbaugh, N. S. , D. L. Swain , and D. Touma . 2015. “Anthropogenic Warming Has Increased Drought Risk in California.” Proceedings of the National Academy of Sciences 112, no. 13: 3931–3936. 10.1073/pnas.1422385112. PMC4386330 25733875

[gcb70246-bib-0031] Fernández‐de‐Uña, L. , J. Martínez‐Vilalta , R. Poyatos , M. Mencuccini , and N. G. McDowell . 2023. “The Role of Height‐Driven Constraints and Compensations on Tree Vulnerability to Drought.” New Phytologist 239, no. 6: 2083–2098. 10.1111/nph.19130. 37485545

[gcb70246-bib-0032] Férriz, M. , D. Martin‐Benito , I. Cañellas , and G. Gea‐Izquierdo . 2021. “Sensitivity to Water Stress Drives Differential Decline and Mortality Dynamics of Three Co‐Occurring Conifers With Different Drought Tolerance.” Forest Ecology and Management 486: 118964. 10.1016/j.foreco.2021.118964.

[gcb70246-bib-0033] Fettig, C. J. , L. A. Mortenson , B. M. Bulaon , and P. B. Foulk . 2019. “Tree Mortality Following Drought in the Central and Southern Sierra Nevada, California, U.S.” Forest Ecology and Management 432: 164–178. 10.1016/j.foreco.2018.09.006.

[gcb70246-bib-0034] Fick, S. E. , and R. J. Hijmans . 2017. “WorldClim 2: New 1‐Km Spatial Resolution Climate Surfaces for Global Land Areas.” International Journal of Climatology 37, no. 12: 4302–4315. 10.1002/joc.5086.

[gcb70246-bib-0035] Fites, J. A. , M. Chappel , B. Corbin , M. Newman , T. Ratcliff , and D. Thomas . 1991. Preliminary Ecological Old‐Growth Definitions for White Fir (SAF Type 211) in California. US Forest Service.

[gcb70246-bib-0036] Fites, J. A. , M. Chappel , B. Corbin , M. Newman , T. Ratcliff , and D. Thomas . 1992. Preliminary Ecological Old‐Growth Definitions for Mixed Conifer (SAF Type 243) in California. US Forest Service.

[gcb70246-bib-0037] Friedman, J. H. 2001. “Greedy Function Approximation: A Gradient Boosting Machine.” Annals of Statistics 29, no. 5: 1189–1232. 10.1214/aos/1013203451.

[gcb70246-bib-0038] Goulden, M. L. , and R. C. Bales . 2019. “California Forest Die‐Off Linked to Multi‐Year Deep Soil Drying in 2012–2015 Drought.” Nature Geoscience 12, no. 8: 632–637. 10.1038/s41561-019-0388-5.

[gcb70246-bib-0039] Green, R. O. , M. L. Eastwood , C. M. Sarture , et al. 1998. “Imaging Spectroscopy and the Airborne Visible/Infrared Imaging Spectrometer (AVIRIS).” Remote Sensing of Environment 65, no. 3: 227–248. 10.1016/S0034-4257(98)00064-9.

[gcb70246-bib-0040] Green, R. O. , N. Mahowald , C. Ung , et al. 2020. “The Earth Surface Mineral Dust Source Investigation: An Earth Science Imaging Spectroscopy Mission.” In 2020 IEEE Aerospace Conference, 1–15. IEEE. 10.1109/AERO47225.2020.9172731.

[gcb70246-bib-0041] Greenwell, B. M. 2017. “Pdp: An R Package for Constructing Partial Dependence Plots.” R Journal 9, no. 1: 421.

[gcb70246-bib-0117] Hartmann, H. , and S. Trumbore . 2016. “Understanding the Roles of Nonstructural Carbohydrates in Forest Trees–from What We Can Measure to What We Want to Know.” New Phytologist 211, no. 2: 386–403. 10.1111/nph.13955. 27061438

[gcb70246-bib-0042] Hartmann, H. , W. Ziegler , O. Kolle , and S. Trumbore . 2013. “Thirst Beats Hunger—Declining Hydration During Drought Prevents Carbon Starvation in Norway Spruce Saplings.” New Phytologist 200, no. 2: 340–349. 10.1111/nph.12331. 23692181

[gcb70246-bib-0043] Hemming‐Schroeder, N. M. , A. A. Gutierrez , S. D. Allison , and J. T. Randerson . 2023. “Estimating Individual Tree Mortality in the Sierra Nevada Using Lidar and Multispectral Reflectance Data.” Journal of Geophysical Research: Biogeosciences 128, no. 5: e2022JG007234. 10.1029/2022JG007234.

[gcb70246-bib-0044] Hijmans, R. J. 2023. *raster: Geographic Data Analysis and Modeling* (R Package Version 3.6–21) [Computer Software]. https://rspatial.org/raster.

[gcb70246-bib-0045] Howe, M. , L. Peng , and A. Carroll . 2022. “Landscape Predictions of Western Balsam Bark Beetle Activity Implicate Warm Temperatures, a Longer Growing Season, and Drought in Widespread Irruptions Across British Columbia.” Forest Ecology and Management 508: 120047. 10.1016/j.foreco.2022.120047.

[gcb70246-bib-0046] Iwasaki, A. , N. Ohgi , J. Tanii , T. Kawashima , and H. Inada . 2011. “Hyperspectral Imager Suite (HISUI)—Japanese Hyper‐Multi Spectral Radiometer.” In 2011 IEEE International Geoscience and Remote Sensing Symposium, 1025–1028. IEEE. 10.1109/IGARSS.2011.6049308.

[gcb70246-bib-0047] Jones, G. M. , J. J. Keane , R. J. Gutiérrez , and M. Z. Peery . 2018. “Declining Old‐Forest Species as a Legacy of Large Trees Lost.” Diversity and Distributions 24, no. 3: 341–351. 10.1111/ddi.12682.

[gcb70246-bib-0048] Kattge, J. , W. Knorr , T. Raddatz , and C. Wirth . 2009. “Quantifying Photosynthetic Capacity and Its Relationship to Leaf Nitrogen Content for Global‐Scale Terrestrial Biosphere Models.” Global Change Biology 15, no. 4: 976–991. 10.1111/j.1365-2486.2008.01744.x.

[gcb70246-bib-0049] Kolb, T. E. , J. K. Agee , P. Z. Fulé , et al. 2007. “Perpetuating Old Ponderosa Pine.” Forest Ecology and Management 249, no. 3: 141–157. 10.1016/j.foreco.2007.06.002.

[gcb70246-bib-0050] Koontz, M. J. , A. M. Latimer , L. A. Mortenson , C. J. Fettig , and M. P. North . 2021. “Cross‐Scale Interaction of Host Tree Size and Climatic Water Deficit Governs Bark Beetle‐Induced Tree Mortality.” Nature Communications 12, no. 1: 129. 10.1038/s41467-020-20455-y. PMC7794511 33420082

[gcb70246-bib-0051] Kuhn, M. 2008. “Building Predictive Models in R Using the Caret Package.” Journal of Statistical Software 28, no. 5: 1–26. 10.18637/jss.v028.i05.27774042

[gcb70246-bib-0052] Kumar, S. V. , P. A. Dirmeyer , C. D. Peters‐Lidard , R. Bindlish , and J. Bolten . 2018. “Information Theoretic Evaluation of Satellite Soil Moisture Retrievals.” Remote Sensing of Environment 204: 392–400. 10.1016/j.rse.2017.10.016. 32636571 PMC7340154

[gcb70246-bib-0053] Kunch, T. 2019. NEON Site‐Level Plot Summary Soaproot Saddle (SOAP). USDA.

[gcb70246-bib-0054] Kunch, T. 2022. NEON Site‐Level Plot Summary Teakettle (TEAK). USDA.

[gcb70246-bib-0055] Lavorel, S. , and E. Garnier . 2002. “Predicting Changes in Community Composition and Ecosystem Functioning From Plant Traits: Revisiting the Holy Grail.” Functional Ecology 16, no. 5: 545–556.

[gcb70246-bib-0056] Lee, C. M. , M. L. Cable , S. J. Hook , et al. 2015. “An Introduction to the NASA Hyperspectral InfraRed Imager (HyspIRI) Mission and Preparatory Activities.” Remote Sensing of Environment 167: 6–19. 10.1016/j.rse.2015.06.012.

[gcb70246-bib-0057] Loizzo, R. , R. Guarini , F. Longo , et al. 2018. “Prisma: The Italian Hyperspectral Mission.” In IGARSS 2018–2018 IEEE International Geoscience and Remote Sensing Symposium, 175–178. IEEE. 10.1109/IGARSS.2018.8518512.

[gcb70246-bib-0058] Lund, J. , J. Medellin‐Azuara , J. Durand , and K. Stone . 2018. “Lessons From California's 2012–2016 Drought.” Journal of Water Resources Planning and Management 144, no. 10: 04018067. 10.1061/(ASCE)WR.1943-5452.0000984.

[gcb70246-bib-0059] Mann, M. E. , and P. H. Gleick . 2015. “Climate Change and California Drought in the 21st Century.” Proceedings of the National Academy of Sciences 112, no. 13: 3858–3859. 10.1073/pnas.1503667112. PMC4386383 25829537

[gcb70246-bib-0060] Marchin, R. M. , M. Esperon‐Rodriguez , M. G. Tjoelker , and D. S. Ellsworth . 2022. “Crown Dieback and Mortality of Urban Trees Linked to Heatwaves During Extreme Drought.” Science of the Total Environment 850: 157915. 10.1016/j.scitotenv.2022.157915. 35944640

[gcb70246-bib-0061] Marconi, S. , B. G. Weinstein , S. Zou , et al. 2022. “Continental‐Scale Hyperspectral Tree Species Classification in the United States National Ecological Observatory Network.” Remote Sensing of Environment 282: 113264. 10.1016/j.rse.2022.113264.

[gcb70246-bib-0062] Maynard, D. S. , L. Bialic‐Murphy , C. M. Zohner , et al. 2022. “Global Relationships in Tree Functional Traits.” Nature Communications 13, no. 1: 3185. 10.1038/s41467-022-30888-2. PMC9177664 35676261

[gcb70246-bib-0063] McDowell, N. G. 2011. “Mechanisms Linking Drought, Hydraulics, Carbon Metabolism, and Vegetation Mortality.” Plant Physiology 155, no. 3: 1051–1059. 10.1104/pp.110.170704. 21239620 PMC3046567

[gcb70246-bib-0064] McDowell, N. G. , W. T. Pockman , C. D. Allen , et al. 2008. “Mechanisms of Plant Survival and Mortality During Drought: Why Do Some Plants Survive While Others Succumb to Drought?” New Phytologist 178, no. 4: 719–739. 10.1111/j.1469-8137.2008.02436.x. 18422905

[gcb70246-bib-0065] Meyer, M. D. , D. A. Kelt , and M. P. North . 2005. “Nest Trees of Northern Flying Squirrels in the Sierra Nevada.” Journal of Mammalogy 86, no. 2: 275–280. 10.1644/BEH-110.1.

[gcb70246-bib-0066] Mitchell, P. J. , A. P. O'Grady , D. T. Tissue , D. Worledge , and E. A. Pinkard . 2014. “Co‐Ordination of Growth, Gas Exchange and Hydraulics Define the Carbon Safety Margin in Tree Species With Contrasting Drought Strategies.” Tree Physiology 34, no. 5: 443–458. 10.1093/treephys/tpu014. 24664613

[gcb70246-bib-0067] Molnar, C. 2019. Interpretable Machine Learning: A Guide for Making Black Box Models Interpretable. Lulu.

[gcb70246-bib-0068] Moore, J. , J. Pope , M. Woods , and A. Ellis . 2018. 2017 Aerial Survey Results: California (Forest Health Monitoring Program). USDA Forest Service.

[gcb70246-bib-0069] Myneni, R. B. , and D. L. Williams . 1994. “On the Relationship Between FAPAR and NDVI.” Remote Sensing of Environment 49, no. 3: 200–211. 10.1016/0034-4257(94)90016-7.

[gcb70246-bib-0070] Nardini, A. 2022. “Hard and Tough: The Coordination Between Leaf Mechanical Resistance and Drought Tolerance.” Flora 288: 152023. 10.1016/j.flora.2022.152023.

[gcb70246-bib-0071] Nieke, J. , and M. Rast . 2018. “Towards the Copernicus Hyperspectral Imaging Mission for the Environment (CHIME).” In IGARSS 2018–2018 IEEE International Geoscience and Remote Sensing Symposium, 157–159. IEEE. 10.1109/IGARSS.2018.8518384.

[gcb70246-bib-0072] Niinemets, Ü. 2016. “Uncovering the Hidden Facets of Drought Stress: Secondary Metabolites Make the Difference.” Tree Physiology 36, no. 2: 129–132. 26687175 10.1093/treephys/tpv128

[gcb70246-bib-0073] Ogaya, R. , and J. Penuelas . 2006. “Contrasting Foliar Responses to Drought in Quercus ilex and Phillyrea Latifolia.” Biologia Plantarum 50, no. 3: 373–382. 10.1007/s10535-006-0052-y.

[gcb70246-bib-0074] Parker, V. T. , and C. H. Muller . 1982. “Vegetational and Environmental Changes Beneath Isolated Live Oak Trees ( *Quercus agrifolia* ) in a California Annual Grassland.” American Midland Naturalist 107, no. 1: 69. 10.2307/2425189.

[gcb70246-bib-0075] Paz‐Kagan, T. , P. G. Brodrick , N. R. Vaughn , et al. 2017. “What Mediates Tree Mortality During Drought in the Southern Sierra Nevada?” Ecological Applications 27, no. 8: 2443–2457. 10.1002/eap.1620. 28871610

[gcb70246-bib-0076] Pfeifer, E. M. , J. A. Hicke , and A. J. H. Meddens . 2011. “Observations and Modeling of Aboveground Tree Carbon Stocks and Fluxes Following a Bark Beetle Outbreak in the Western United States: Carbon Cycling Following Bark Beetle Outbreak.” Global Change Biology 17, no. 1: 339–350. 10.1111/j.1365-2486.2010.02226.x.

[gcb70246-bib-0077] Pflug, E. E. , N. Buchmann , R. T. Siegwolf , M. Schaub , A. Rigling , and M. Arend . 2018. “Resilient Leaf Physiological Response of European Beech (*Fagus sylvatica* L.) to Summer Drought and Drought Release.” Frontiers in Plant Science 9: 187. 29515605 10.3389/fpls.2018.00187 PMC5825912

[gcb70246-bib-0078] Poorter, H. , Ü. Niinemets , L. Poorter , I. J. Wright , and R. Villar . 2009. “Causes and Consequences of Variation in Leaf Mass per Area (LMA): A meta‐Analysis.” New Phytologist 182, no. 3: 565–588. 10.1111/j.1469-8137.2009.02830.x. 19434804

[gcb70246-bib-0079] Prasad, A. M. , L. R. Iverson , and A. Liaw . 2006. “Newer Classification and Regression Tree Techniques: Bagging and Random Forests for Ecological Prediction.” Ecosystems 9, no. 2: 181–199. 10.1007/s10021-005-0054-1.

[gcb70246-bib-0080] Rambo, T. R. , and M. P. North . 2009. “Canopy Microclimate Response to Pattern and Density of Thinning in a Sierra Nevada Forest.” Forest Ecology and Management 257, no. 2: 435–442. 10.1016/j.foreco.2008.09.029.

[gcb70246-bib-0081] Restaino, C. , D. J. N. Young , B. Estes , et al. 2019. “Forest Structure and Climate Mediate Drought‐Induced Tree Mortality in Forests of the Sierra Nevada, USA.” Ecological Applications 29, no. 4: e01902. 10.1002/eap.1902. 31020735

[gcb70246-bib-0082] Robbins, Z. J. , C. Xu , B. H. Aukema , et al. 2022. “Warming Increased Bark Beetle‐Induced Tree Mortality by 30% During an Extreme Drought in California.” Global Change Biology 28, no. 2: 509–523. 10.1111/gcb.15927. 34713535

[gcb70246-bib-0083] Russell, M. B. , C. W. Woodall , A. W. D'Amato , G. M. Domke , and S. S. Saatchi . 2014. “Beyond Mean Functional Traits: Influence of Functional Trait Profiles on Forest Structure, Production, and Mortality Across the Eastern US.” Forest Ecology and Management 328: 1–9. 10.1016/j.foreco.2014.05.014.

[gcb70246-bib-0084] Sack, L. , and T. N. Buckley . 2020. “Trait Multi‐Functionality in Plant Stress Response.” Integrative and Comparative Biology 60, no. 1: 98–112. 10.1093/icb/icz152. 31825509

[gcb70246-bib-0085] Sala, A. , F. Piper , and G. Hoch . 2010. “Physiological Mechanisms of Drought‐Induced Tree Mortality Are Far From Being Resolved.” New Phytologist 186, no. 2: 274–281. 10.1111/j.1469-8137.2009.03167.x. 20409184

[gcb70246-bib-0086] Sancho‐Knapik, D. , A. Escudero , S. Mediavilla , et al. 2021. “Deciduous and Evergreen Oaks Show Contrasting Adaptive Responses in Leaf Mass per Area Across Environments.” New Phytologist 230, no. 2: 521–534. 10.1111/nph.17151. 33340114

[gcb70246-bib-0087] Scheffler, D. , A. Hollstein , H. Diedrich , K. Segl , and P. Hostert . 2017. “AROSICS: An Automated and Robust Open‐Source Image Co‐Registration Software for Multi‐Sensor Satellite Data.” Remote Sensing 9, no. 7: 676. 10.3390/rs9070676.

[gcb70246-bib-0088] Sevanto, S. , N. G. McDowell , L. T. Dickman , R. Pangle , and W. T. Pockman . 2014. “How Do Trees Die? A Test of the Hydraulic Failure and Carbon Starvation Hypotheses.” Plant, Cell & Environment 37, no. 1: 153–161. 10.1111/pce.12141. PMC4280888 23730972

[gcb70246-bib-0089] Shipley, B. , F. De Bello , J. H. C. Cornelissen , E. Laliberté , D. C. Laughlin , and P. B. Reich . 2016. “Reinforcing Loose Foundation Stones in Trait‐Based Plant Ecology.” Oecologia 180, no. 4: 923–931. 10.1007/s00442-016-3549-x. 26796410

[gcb70246-bib-0090] Singh, A. , S. P. Serbin , B. E. McNeil , C. C. Kingdon , and P. A. Townsend . 2015. “Imaging Spectroscopy Algorithms for Mapping Canopy Foliar Chemical and Morphological Traits and Their Uncertainties.” Ecological Applications 25, no. 8: 2180–2197. 10.1890/14-2098.1. 26910948

[gcb70246-bib-0091] Stavros, E. N. , J. Chrone , K. Cawse‐Nicholson , et al. 2023. “Designing an Observing System to Study the Surface Biology and Geology (SBG) of the Earth in the 2020s.” Journal of Geophysical Research: Biogeosciences 128, no. 1: e2021JG006471. 10.1029/2021JG006471. PMC10286770 37362830

[gcb70246-bib-0092] Stephens, S. L. , B. M. Collins , C. J. Fettig , et al. 2018. “Drought, Tree Mortality, and Wildfire in Forests Adapted to Frequent Fire.” Bioscience 68, no. 2: 77–88. 10.1093/biosci/bix146.

[gcb70246-bib-0093] Stephenson, N. L. , and A. J. Das . 2020. “Height‐Related Changes in Forest Composition Explain Increasing Tree Mortality With Height During an Extreme Drought.” Nature Communications 11, no. 1: 3402. 10.1038/s41467-020-17213-5. PMC7341764 32636488

[gcb70246-bib-0094] Stephenson, N. L. , A. J. Das , N. J. Ampersee , B. M. Bulaon , and J. L. Yee . 2019. “Which Trees Die During Drought? The Key Role of Insect Host‐Tree Selection.” Journal of Ecology 107, no. 5: 2383–2401. 10.1111/1365-2745.13176.

[gcb70246-bib-0095] Stovall, A. E. L. , H. Shugart , and X. Yang . 2019. “Tree Height Explains Mortality Risk During an Intense Drought.” Nature Communications 10, no. 1: 4385. 10.1038/s41467-019-12380-6. PMC6763443 31558795

[gcb70246-bib-0096] Stovall, A. E. L. , H. H. Shugart , and X. Yang . 2020. “Reply to ‘Height‐Related Changes in Forest Composition Explain Increasing Tree Mortality With Height During an Extreme Drought.’.” Nature Communications 11, no. 1: 3401. 10.1038/s41467-020-17214-4. PMC7340790 32636374

[gcb70246-bib-0097] Stuffler, T. , C. Kaufmann , S. Hofer , et al. 2007. “The EnMAP Hyperspectral Imager—An Advanced Optical Payload for Future Applications in Earth Observation Programmes.” Acta Astronautica 61, no. 1–6: 115–120. 10.1016/j.actaastro.2007.01.033.

[gcb70246-bib-0098] Suding, K. N. , S. Lavorel , F. S. Chapin , et al. 2008. “Scaling Environmental Change Through the Community‐Level: A Trait‐Based Response‐And‐Effect Framework for Plants: Scaling Community‐Level Processes.” Global Change Biology 14, no. 5: 1125–1140. 10.1111/j.1365-2486.2008.01557.x.

[gcb70246-bib-0099] Swatantran, A. , R. Dubayah , D. Roberts , M. Hofton , and J. B. Blair . 2011. “Mapping Biomass and Stress in the Sierra Nevada Using Lidar and Hyperspectral Data Fusion.” Remote Sensing of Environment 115, no. 11: 2917–2930. 10.1016/j.rse.2010.08.027.

[gcb70246-bib-0100] Thornton, P. E. , R. Shrestha , M. Thornton , S.‐C. Kao , Y. Wei , and B. E. Wilson . 2021. “Gridded Daily Weather Data for North America With Comprehensive Uncertainty Quantification.” Scientific Data 8, no. 1: 190. 10.1038/s41597-021-00973-0. 34301954 PMC8302764

[gcb70246-bib-0101] Thornton, M. M. , R. Shrestha , Y. Wei , P. E. Thornton , S.‐C. Kao , and B. E. Wilson . 2022. Daymet: Monthly Climate Summaries on a 1‐km Grid for North America, Version 4 R1 [netCDF, GTiff]. 0 MB. 10.3334/ORNLDAAC/2131.

[gcb70246-bib-0102] Trujillo, E. , N. P. Molotch , M. L. Goulden , A. E. Kelly , and R. C. Bales . 2012. “Elevation‐Dependent Influence of Snow Accumulation on Forest Greening.” Nature Geoscience 5, no. 10: 705–709. 10.1038/ngeo1571.

[gcb70246-bib-0118] Tucker, C. J. 1980. “Remote Sensing of Leaf Water Content in the Near Infrared.” Remote Sensing of Environment 10, no. 1: 23–32. 10.1016/0034-4257(80)90096-6.

[gcb70246-bib-0103] USDA . 2003. National Agriculture Imagery Program (NAIP) Orthoimagery [Dataset]. 10.5066/F7QN651G.

[gcb70246-bib-0104] USDA—Forest Service . 2016. Existing Vegetation—CALVEG. Southern Sierra Region. https://data.fs.usda.gov/geodata/edw/datasets.php.

[gcb70246-bib-0105] USDA and NOAA . 2023. Time Series. U.S. Drought Monitor. https://droughtmonitor.unl.edu/DmData/TimeSeries.aspx.

[gcb70246-bib-0106] Vergopolan, N. , N. W. Chaney , M. Pan , et al. 2021. “SMAP‐HydroBlocks, a 30‐m Satellite‐Based Soil Moisture Dataset for the Conterminous US.” Scientific Data 8, no. 1: 264. 10.1038/s41597-021-01050-2. 34635675 PMC8505542

[gcb70246-bib-0107] Vergopolan, N. , J. Sheffield , N. W. Chaney , et al. 2022. “High‐Resolution Soil Moisture Data Reveal Complex Multi‐Scale Spatial Variability Across the United States.” Geophysical Research Letters 49, no. 15: 1–13. 10.1029/2022GL098586.35928231

[gcb70246-bib-0108] Wahl, E. R. , E. Zorita , H. F. Diaz , and A. Hoell . 2022. “Southwestern United States Drought of the 21st Century Presages Drier Conditions Into the Future.” Communications Earth & Environment 3, no. 1: 202. 10.1038/s43247-022-00532-4.

[gcb70246-bib-0109] Wang, Z. , A. Chlus , R. Geygan , et al. 2020. “Foliar Functional Traits From Imaging Spectroscopy Across Biomes in Eastern North America.” New Phytologist 228, no. 2: 494–511. 10.1111/nph.16711. 32463927

[gcb70246-bib-0110] Williams, A. P. , B. I. Cook , and J. E. Smerdon . 2022. “Rapid Intensification of the Emerging Southwestern North American Megadrought in 2020–2021.” Nature Climate Change 12, no. 3: 232–234. 10.1038/s41558-022-01290-z.

[gcb70246-bib-0111] Wright, I. J. , P. B. Reich , M. Westoby , et al. 2004. “The Worldwide Leaf Economics Spectrum.” Nature 428, no. 6985: 821–827. 10.1038/nature02403. 15103368

[gcb70246-bib-0112] Wright, M. N. , and A. Ziegler . 2017. “Ranger: A Fast Implementation of Random Forests for High Dimensional Data in C++ and R.” Journal of Statistical Software 77, no. 1: 1–17. 10.18637/jss.v077.i01.

[gcb70246-bib-0113] Young, D. J. , M. Meyer , B. Estes , et al. 2020. “Forest Recovery Following Extreme Drought in California, USA: Natural Patterns and Effects of Pre‐Drought Management.” Ecological Applications 30, no. 1: e02002. 31519065 10.1002/eap.2002

[gcb70246-bib-0114] Zheng, T. , Z. Ye , A. Singh , et al. 2024. “Variability in Forest Plant Traits Along the Western Ghats of India and Their Environmental Drivers at Different Resolutions.” Journal of Geophysical Research: Biogeosciences 129, no. 3: e2023JG007753.

[gcb70246-bib-0115] van Zyl, J. J. 2001. “The Shuttle Radar Topography Mission (SRTM): A Breakthrough in Remote Sensing of Topography.” Acta Astronautica 48, no. 5–12: 559–565. 10.1016/S0094-5765(01)00020-0.

