# Supplementary material for: Functional Traits From Imaging Spectroscopy Inform Patterns of Forest Mortality During Sierra Nevada Drought
Source: Glob Chang Biol. 2025 May 15;31(5):e70246. doi: 10.1111/gcb.70246 (PMC12079731; doi:10.1111/gcb.70246)
Supplement: Supplementary file 1 — Figure S1. Trait mapping workflow used for this study. Blue boxes indicate data inputs, white boxes indicate processing steps, orange boxes indicate intermediate data products, and the green box indicates the final trait map product. The gray box describes the PLSR model training/testing workflow in greater detail. Major image processing steps include: topographic and BRDF corrections (applied using FlexBRDF; Queally et al., 2022) to reduce the influence of unwanted brightness gradients from terrain and solar/sensor geometries on trait estimates; and alignment to the Landsat grid (using AROSICS; Scheffler et al. 2017) to improve year‐to‐year comparisons across pixels. Figure S1 References Figure S2. The seasonal trend in MODIS NDVI shows that 2013 and 2014 image acquisitions (2013‐06‐12 and 2014‐06‐03, respectively) occurred during similar phenological periods. At Soaproot, the acquisitions occur just after a greenness peak (though note the corresponding peak is relatively lower in 2014 as compared to 2013). At Teakettle, the acquisitions occur leading up to peak greenness. Figure S3. Mean model predictions across 500 model permutations shown in blue, with standard deviation indicated by horizontal bars. 1:1 line shown in black. Figure S4. We observed high correlation amongst some traits (a), and amongst some climate variables and elevation (b), with variation across the two sites. Figure S5. Influence of high sensor zenith angle on NAIP imagery in Stovall et al. (2019) study at Teakettle. Large differences between the 2017 (Hemming‐Schroeder et al.) and 2016 (Stovall) mortality data show patchiness (a). These same patches are evident in the 2016 mortality data alone, and align precisely with flight line boundaries across dates (b). Imagery from the June 30 acquisition show high sensor zenith angle, and resulting oblique view of forest canopy (c). Areas outlined in pink (6/30/16 acquisition) were masked at the Teakettle site, with 52.4% of pixels retained. Figure S6 [file GCB-31-e70246-s001.docx]

# Supporting Information for

**Functional traits from imaging spectroscopy inform patterns of forest mortality during Sierra Nevada drought**

Natalie Queally^1^, Ting Zheng^1^, Zhiwei Ye^1^, Kyle R. Kovach^1^, Ryan Pavlick^2^, Ethan Shafron^2,3^, Fabian D. Schneider^2,4,5^, Philip A. Townsend^1^

^1^Department of Forest and Wildlife Ecology, University of Wisconsin-Madison, 1630 Linden Drive, Madison, WI 53706, USA. ^2^Jet Propulsion Laboratory, California Institute of Technology, 4800 Oak Grove Drive, Pasadena, CA 91109, USA. ^3^Department of Ecosystem and Conservation Sciences, University of Montana, Missoula, MT 59812, USA. ^4^Aarhus University, Department of Biology, Section for Ecoinformatics and Biodiversity, Ny Munkegade 116, 8000 Aarhus, Denmark. ^5^Pioneer Center for Landscape Research in Sustainable Agricultural Futures (Land-CRAFT), 8000 Aarhus, Denmark.

**Contents of this file**

Figures S1 to S8

Tables S1 to S3

**Introduction**

The supporting tables and figures show additional methods, results, and data exploration that are not crucial to the conclusion of the paper but provide context for decisions regarding methodology and discussion of results. These figures and tables include trait mapping workflow, variable importance and partial dependence plots for all species and variables, and further information regarding trait model performance.


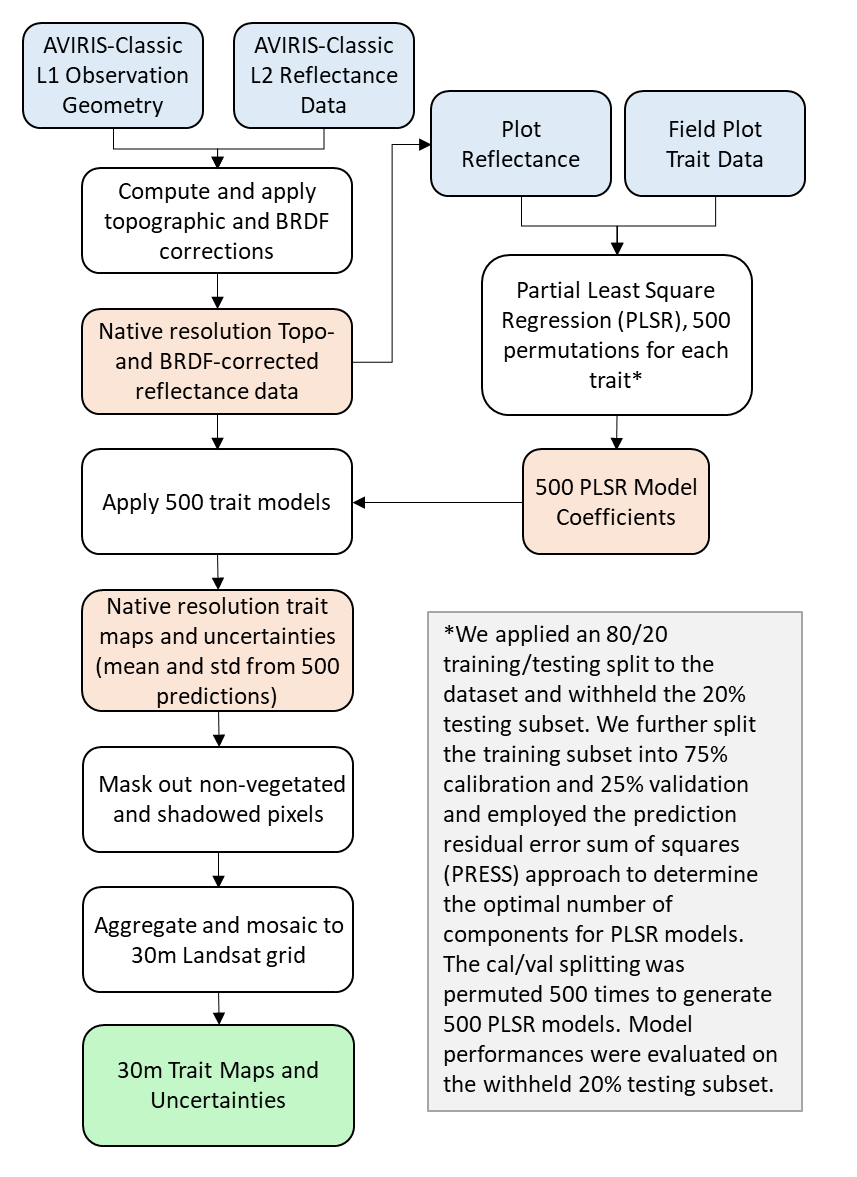


## **Figure S1.** Trait mapping workflow used for this study. Blue boxes indicate data inputs, white boxes indicate processing steps, orange boxes indicate intermediate data products, and the green box indicates the final trait map product. The gray box describes the PLSR model training/testing workflow in greater detail. Major image processing steps include: topographic and BRDF corrections (applied using FlexBRDF; Queally et al., 2022) to reduce the influence of unwanted brightness gradients from terrain and solar/sensor geometries on trait estimates; and alignment to the Landsat grid (using AROSICS; Scheffler et al., 2017) to improve year-to-year comparisons across pixels.

Figure S1 References

Queally, N., Ye, Z., Zheng, T., Chlus, A., Schneider, F., Pavlick, R. P., & Townsend, P. A.

(2022). FlexBRDF: A flexible BRDF correction for grouped processing of airborne imaging spectroscopy flightlines. *Journal of Geophysical Research: Biogeosciences*, *127*(1), e2021JG006622.

Scheffler, D., Hollstein, A., Diedrich, H., Segl, K., & Hostert, P. (2017). AROSICS: An

automated and robust open-source image co-registration software for multi-sensor

satellite data. *Remote sensing*, *9*(7), 676.


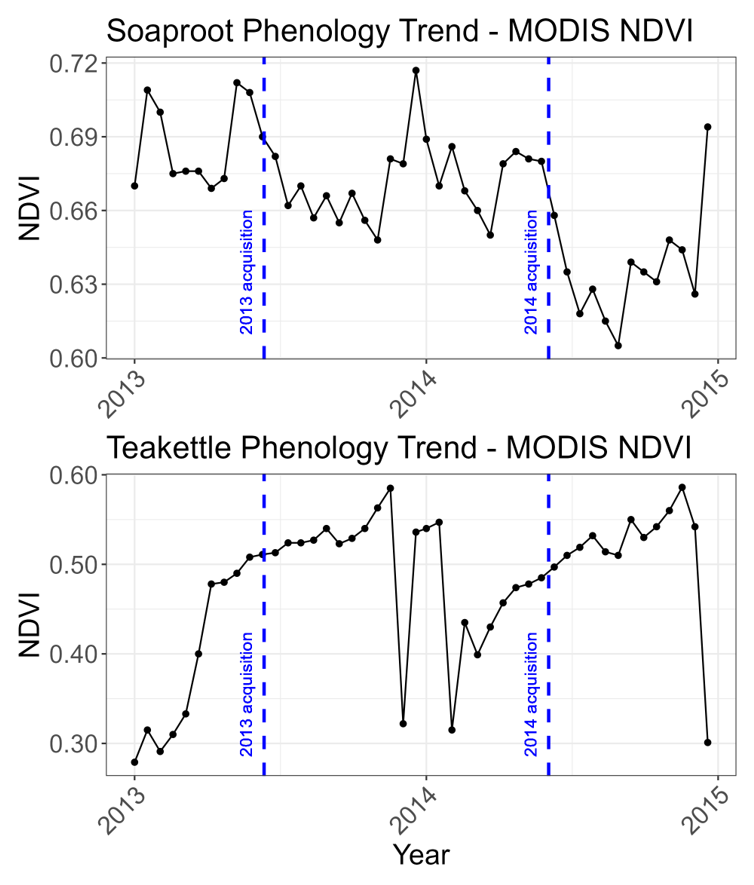


## **Figure S2.** The seasonal trend in MODIS NDVI shows that 2013 and 2014 image acquisitions (2013-06-12 and 2014-06-03, respectively) occurred during similar phenological periods. At Soaproot, the acquisitions occur just after a greenness peak (though note the corresponding peak is relatively lower in 2014 as compared to 2013). At Teakettle, the acquisitions occur leading up to peak greenness.


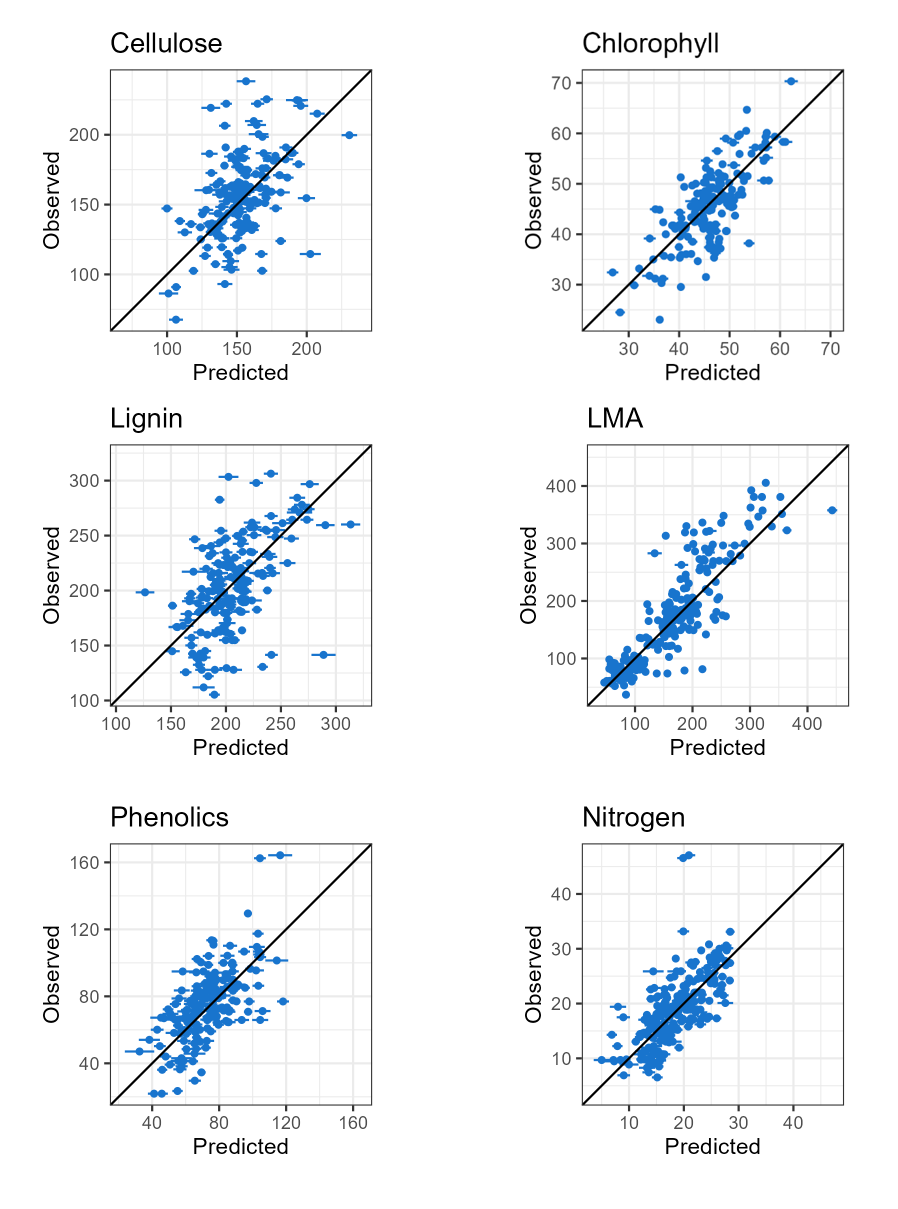

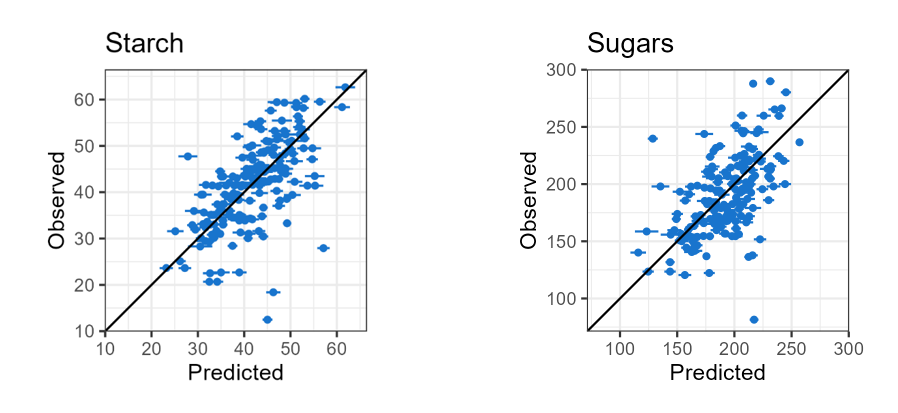


## **Figure S3.** Mean model predictions across 500 model permutations shown in blue, with standard deviation indicated by horizontal bars. 1:1 line shown in black.


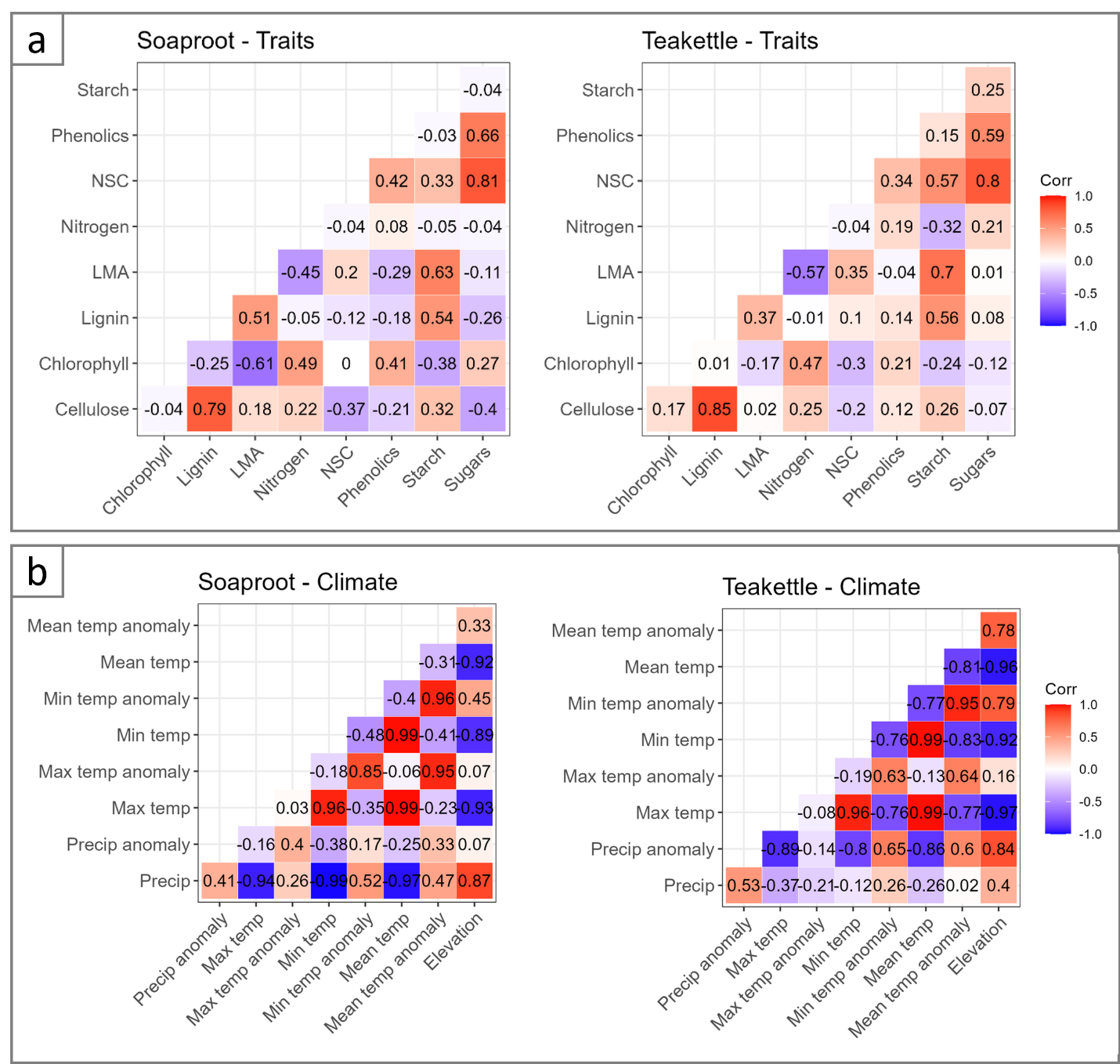


## **Figure S4.** We observed high correlation amongst some traits (a), and amongst some climate variables and elevation (b), with variation across the two sites.


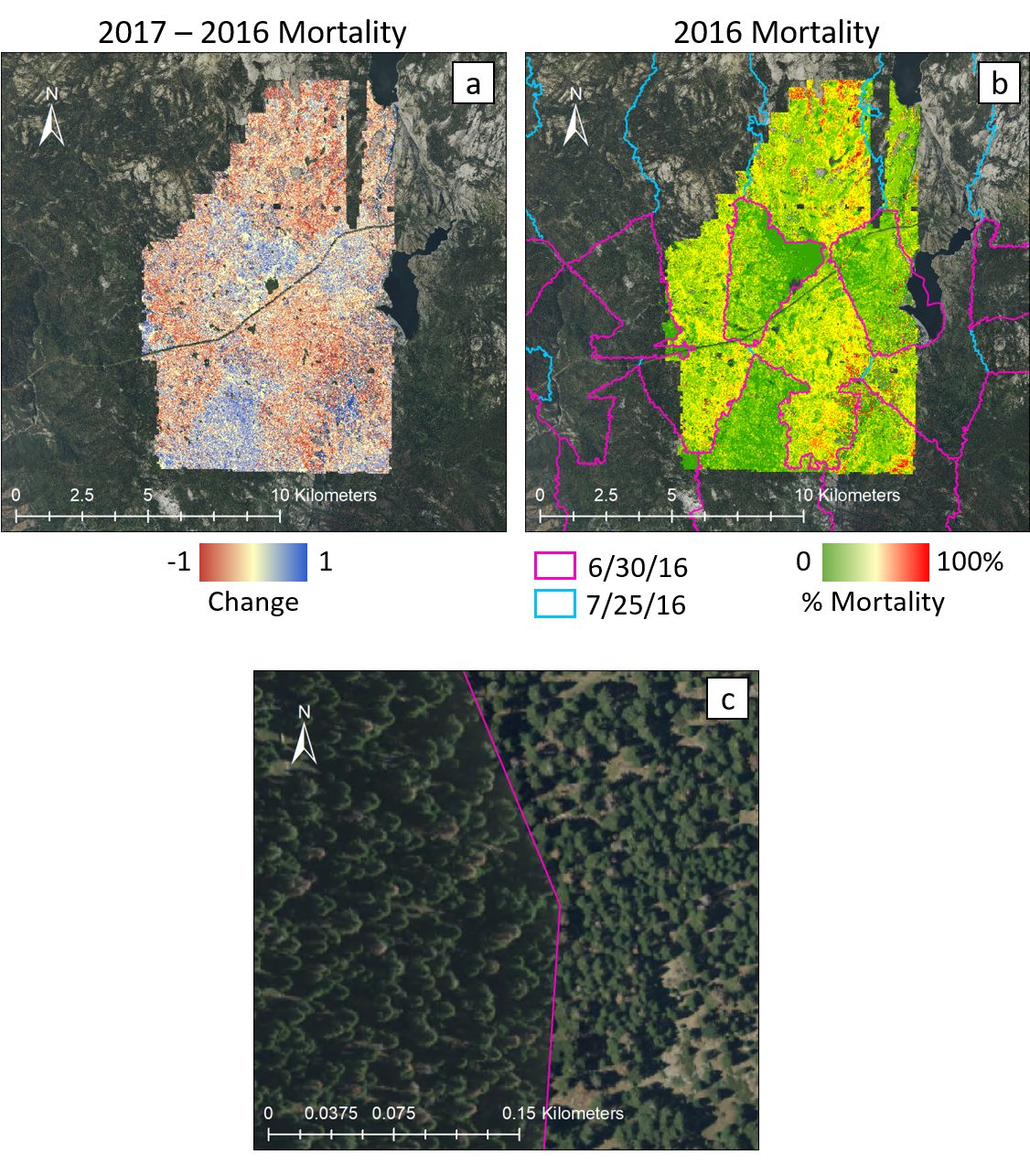


## **Figure S5.** Influence of high sensor zenith angle on NAIP imagery in Stovall et al. (2019) study at Teakettle. Large differences between the 2017 (Hemming-Schroeder et al.) and 2016 (Stovall) mortality data show patchiness (a). These same patches are evident in the 2016 mortality data alone, and align precisely with flight line boundaries across dates (b). Imagery from the June 30 acquisition show high sensor zenith angle, and resulting oblique view of forest canopy (c). Areas outlined in pink (6/30/16 acquisition) were masked at the Teakettle site, with 52.4% of pixels retained.


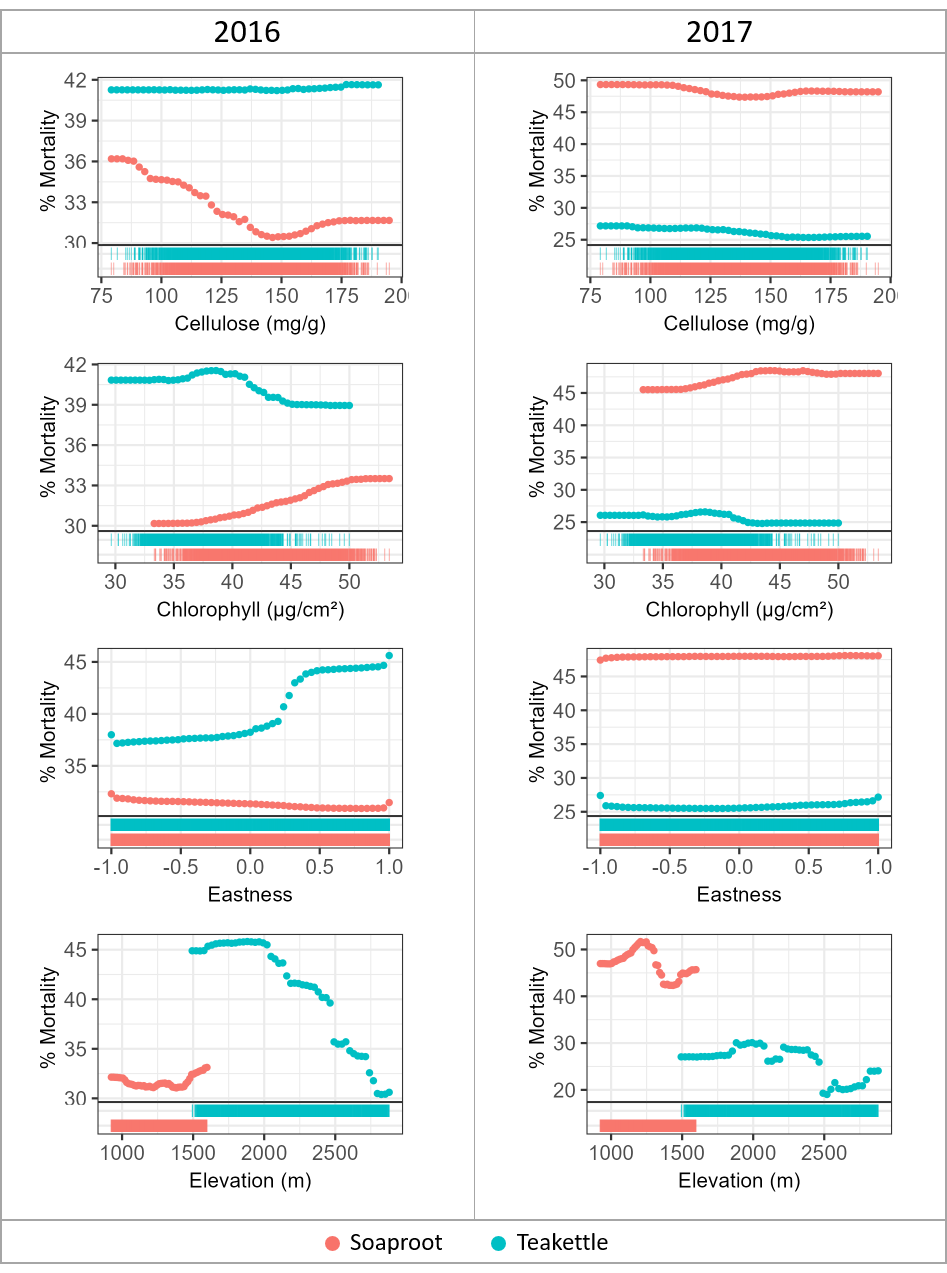

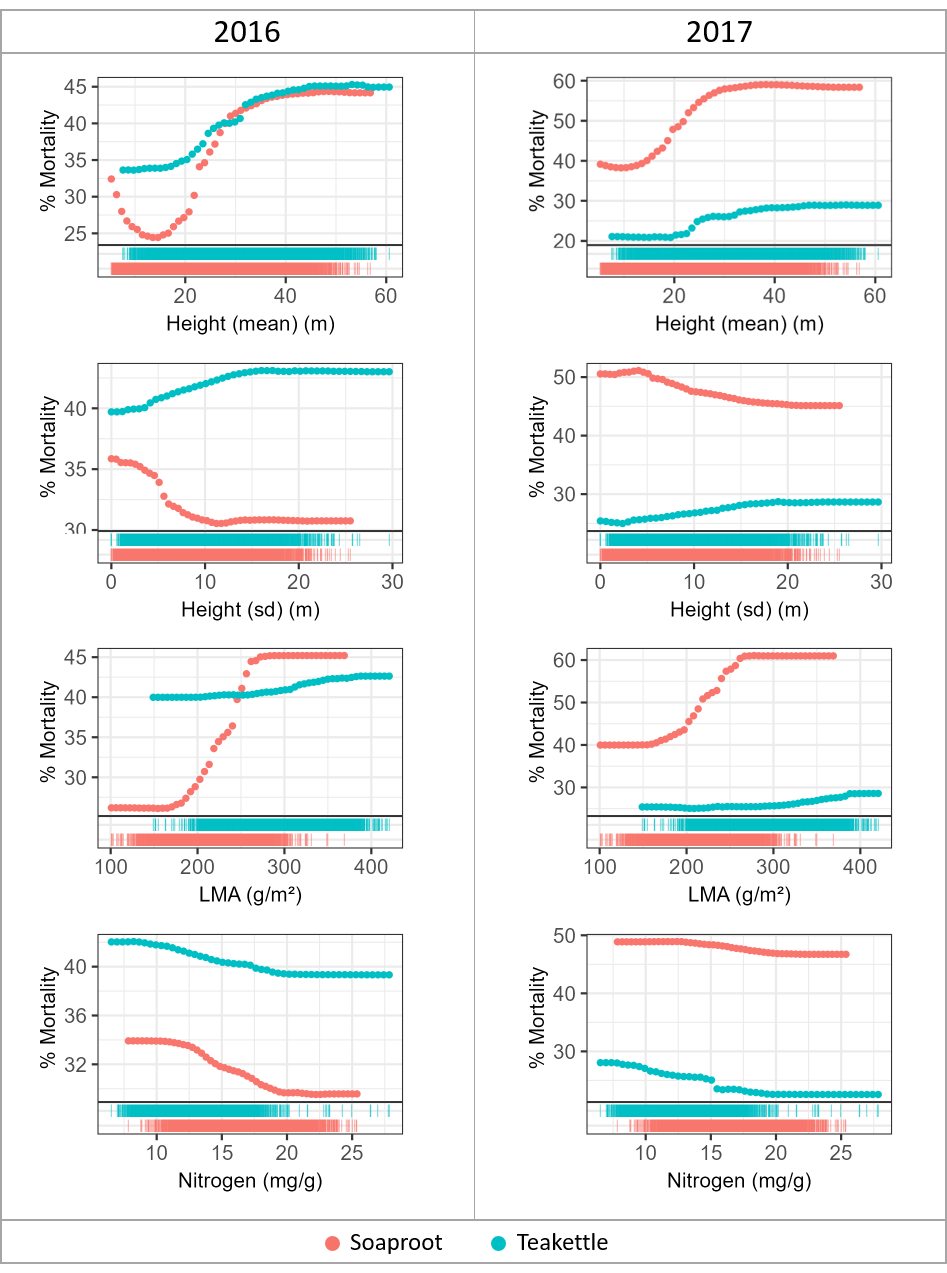

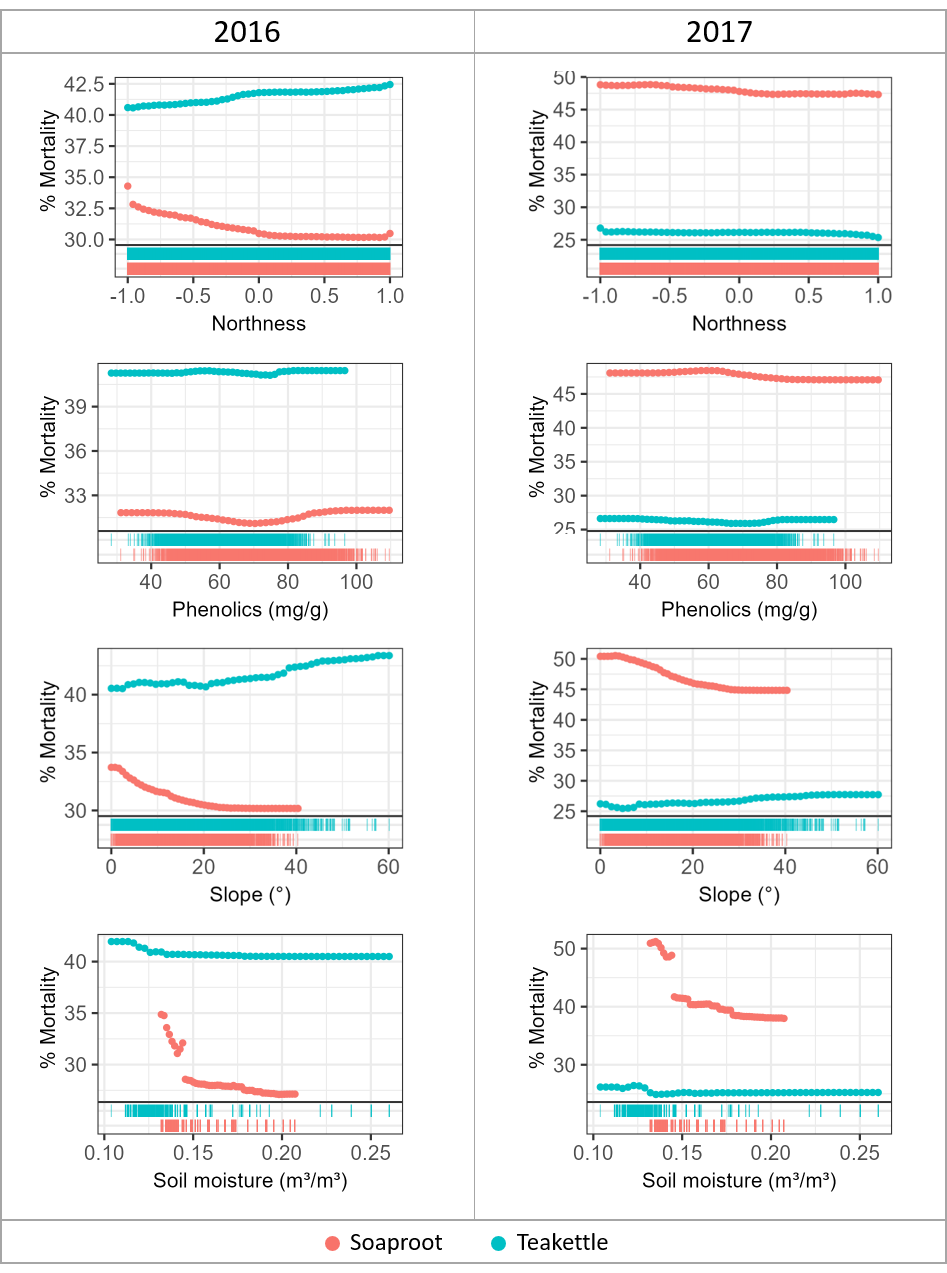

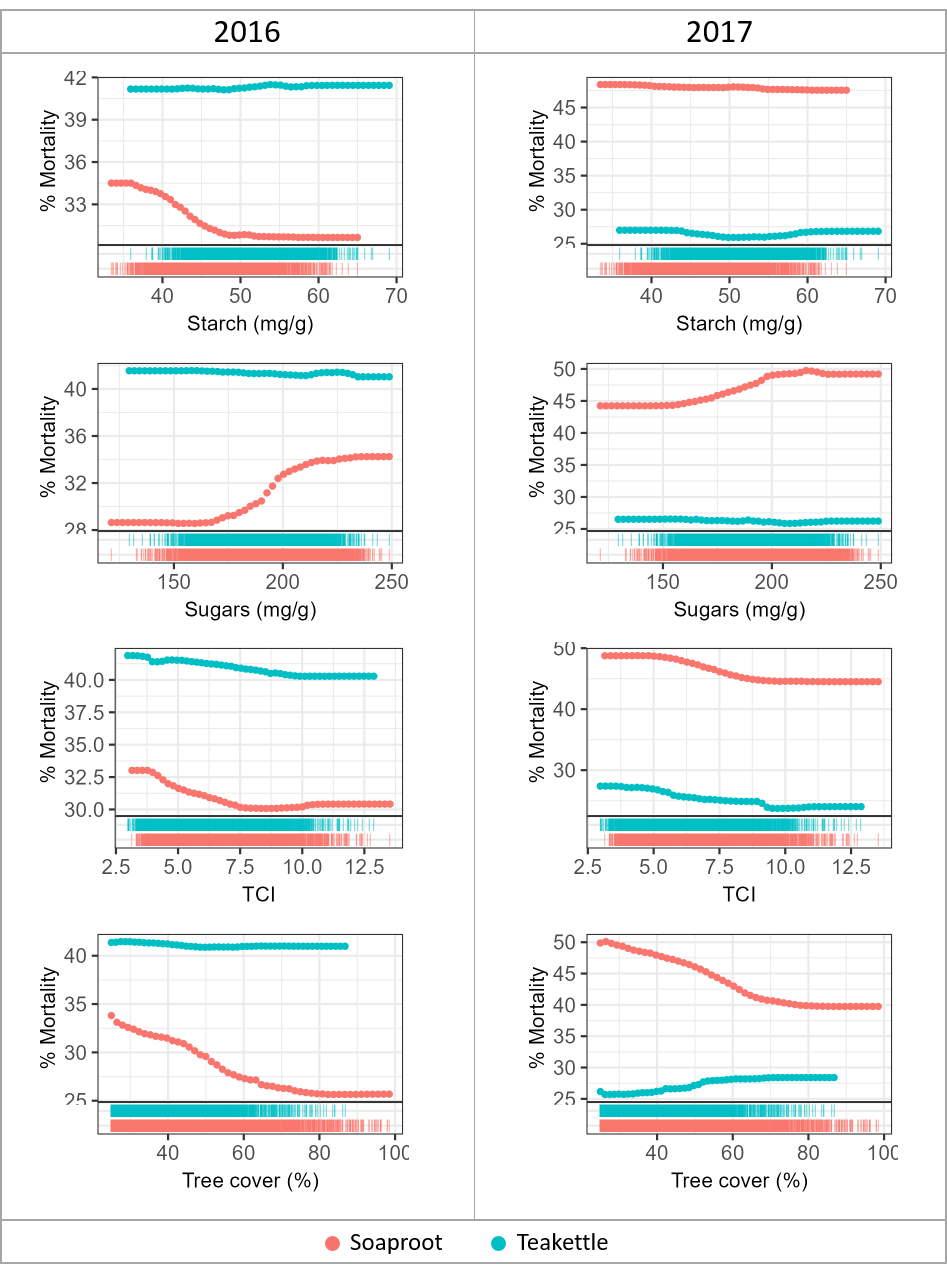

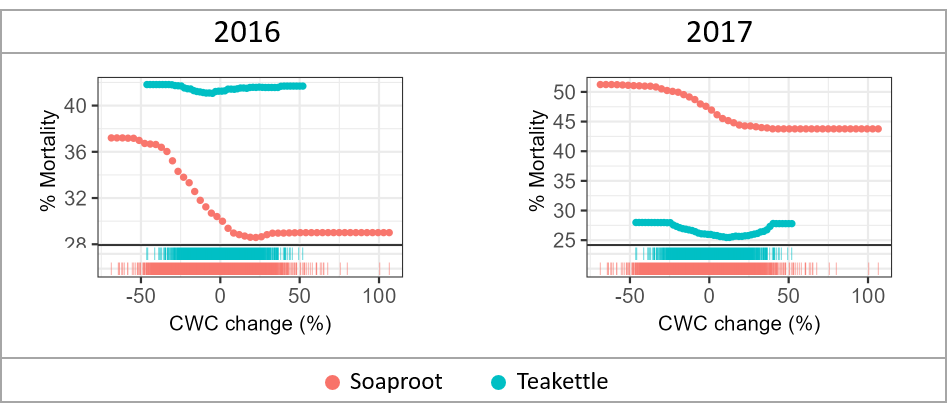


## **Figure S6.** Site level partial dependence plots show mortality trend across all predictor gradients. Accompanying rug plots show site-specific data distributions. Results are shown separately for models using 2016 and 2017 mortality. General trends are similar for top predictors (e.g., increasing mortality with increasing mean tree height), though magnitude of mortality varies across mortality data source.


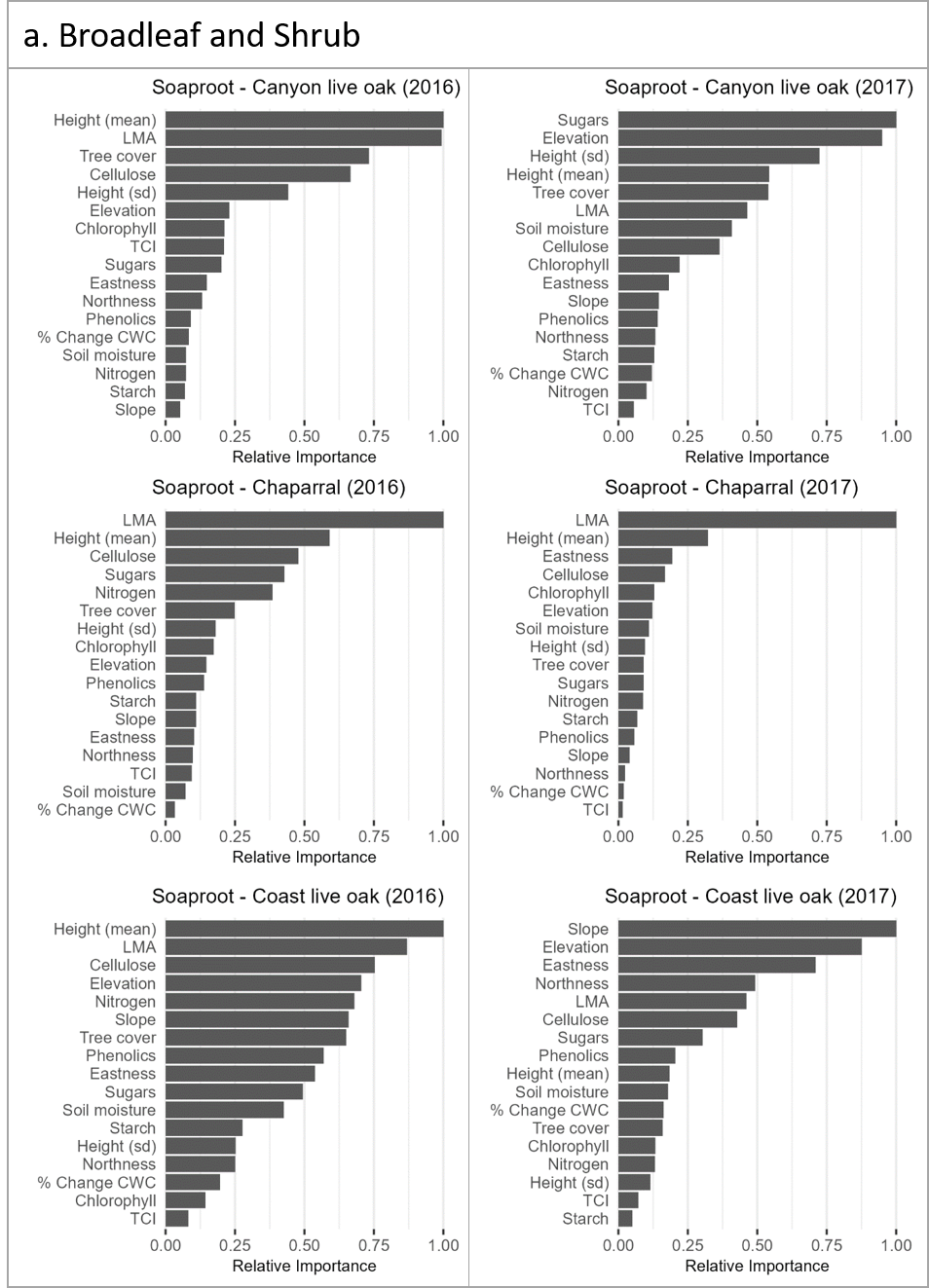

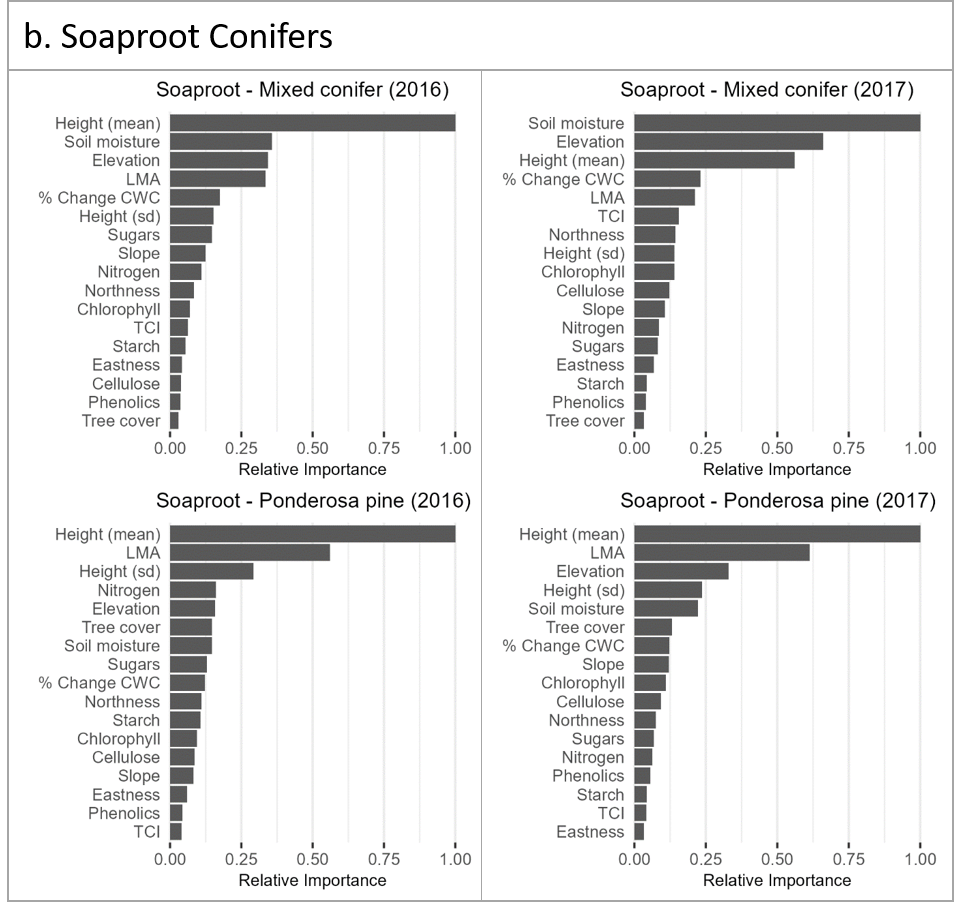

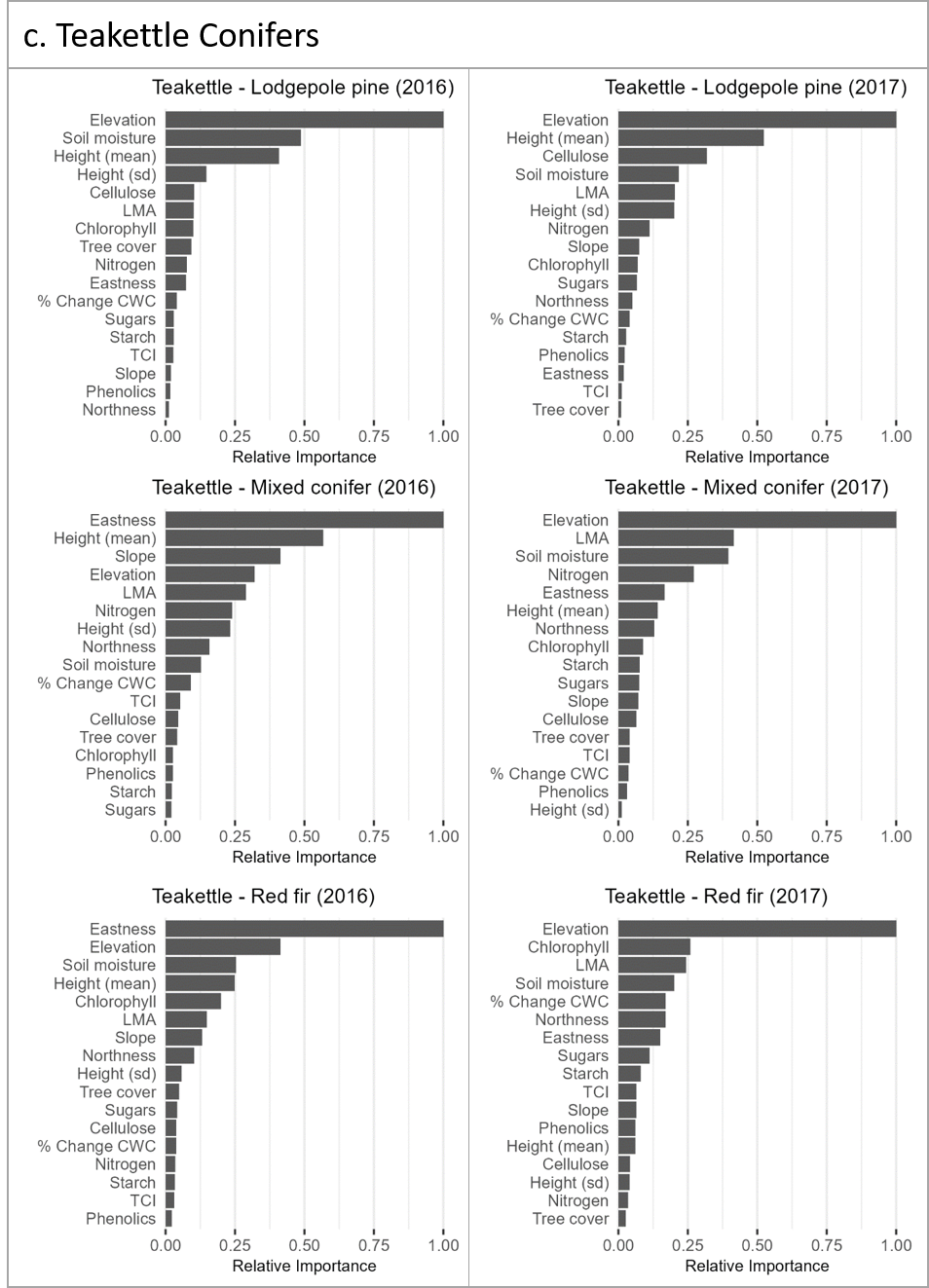

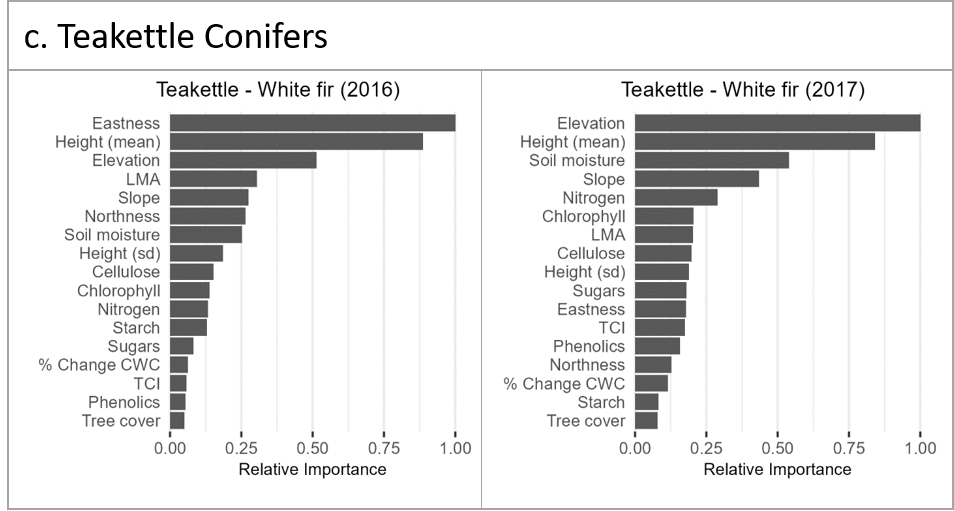


## **Figure S7.** Variable importance of species-level models using 2016 mortality data (left) and 2017 mortality data (right).


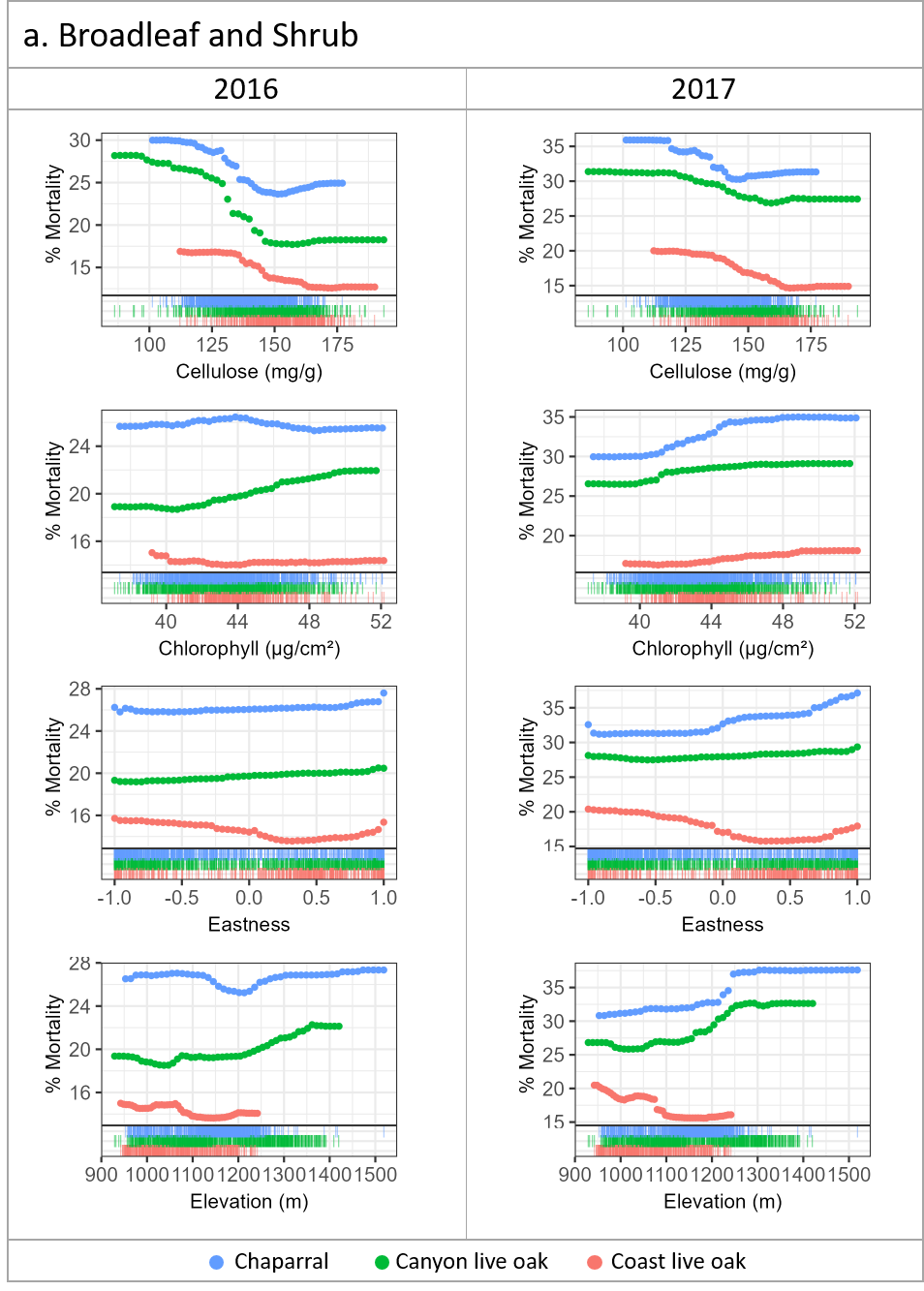

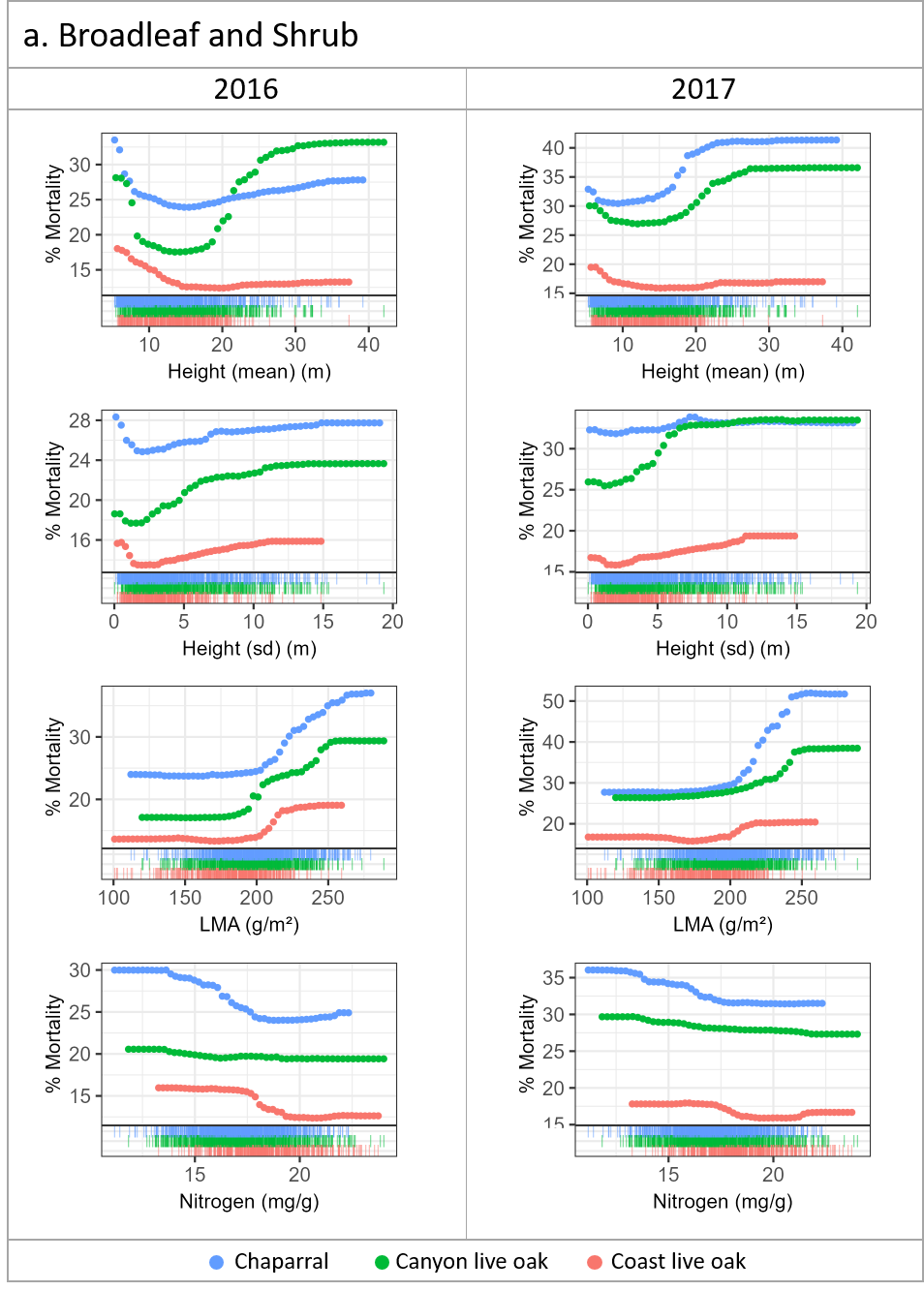

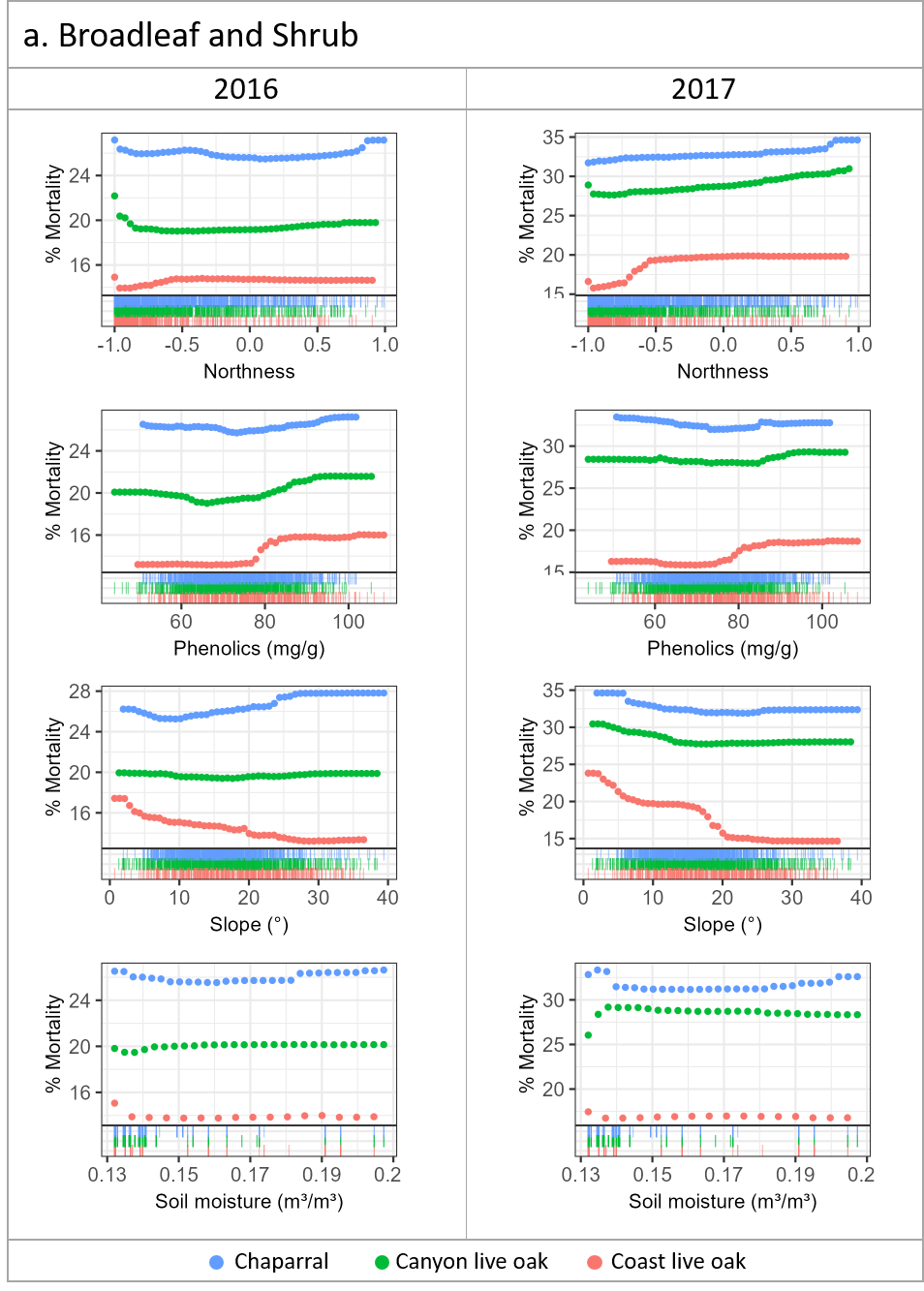

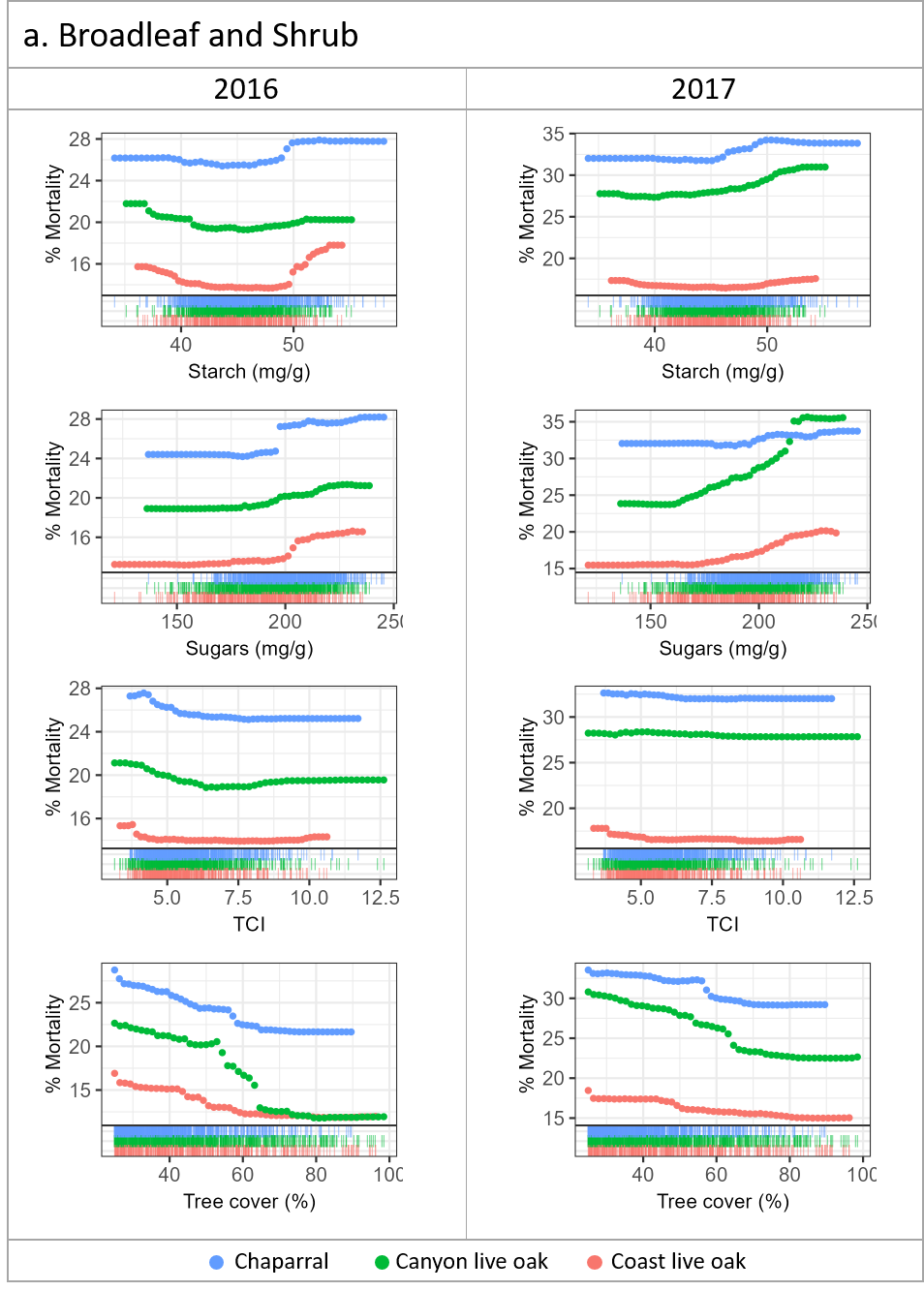

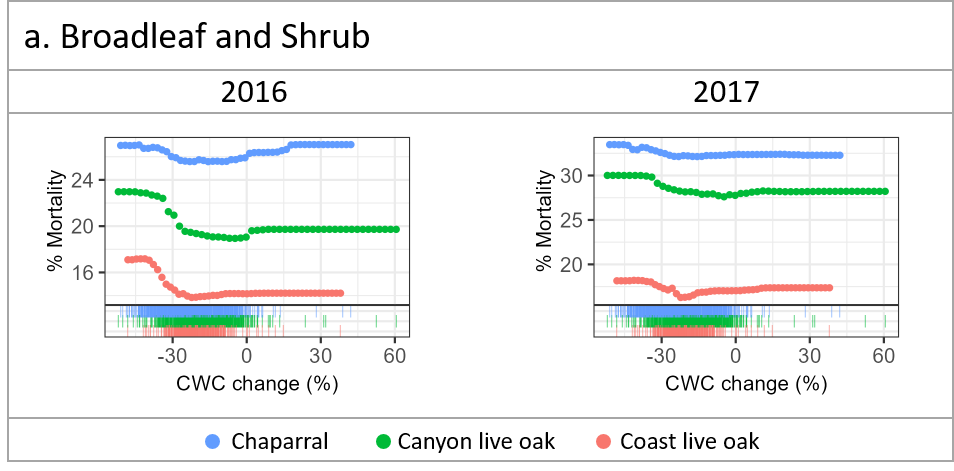


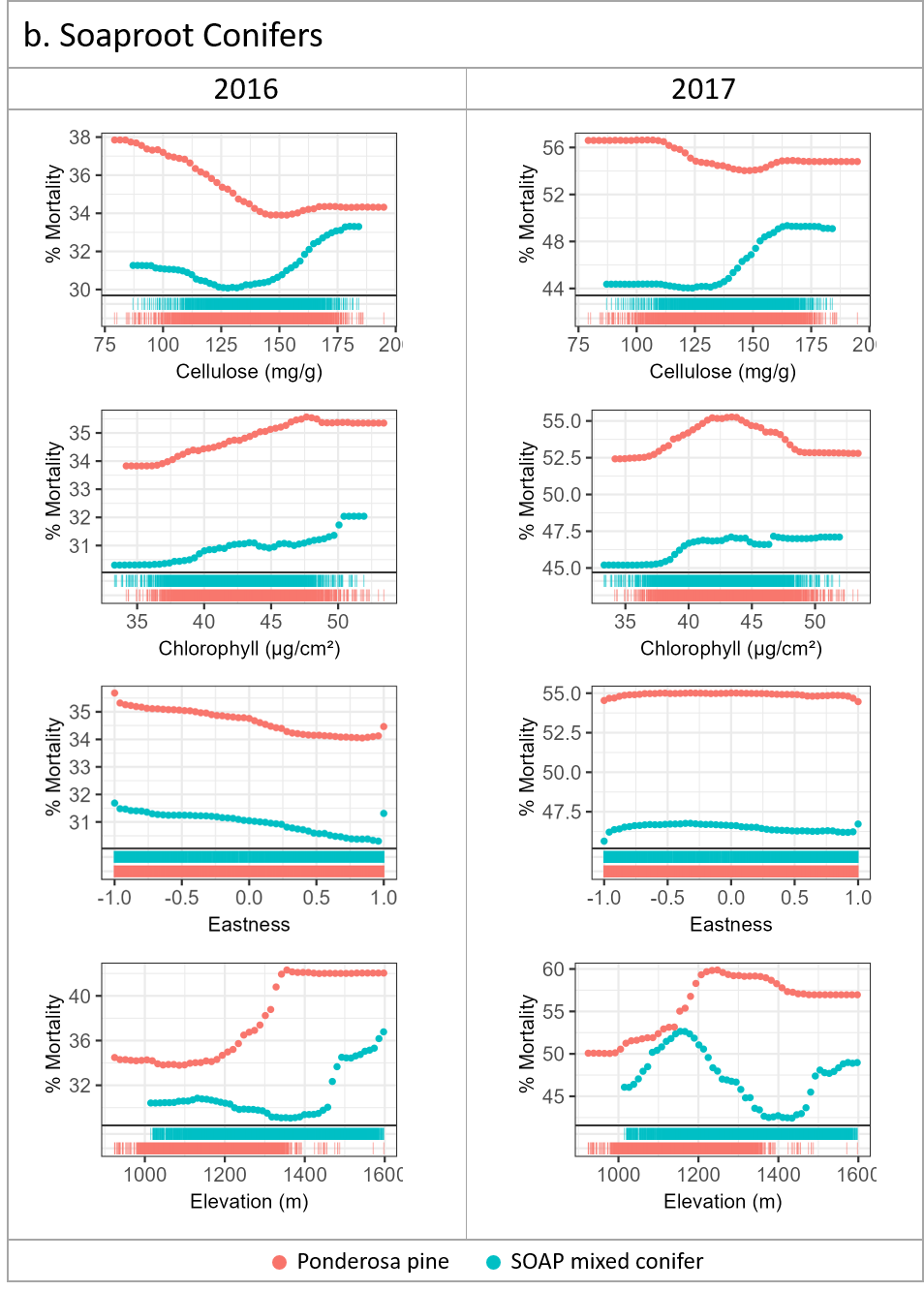

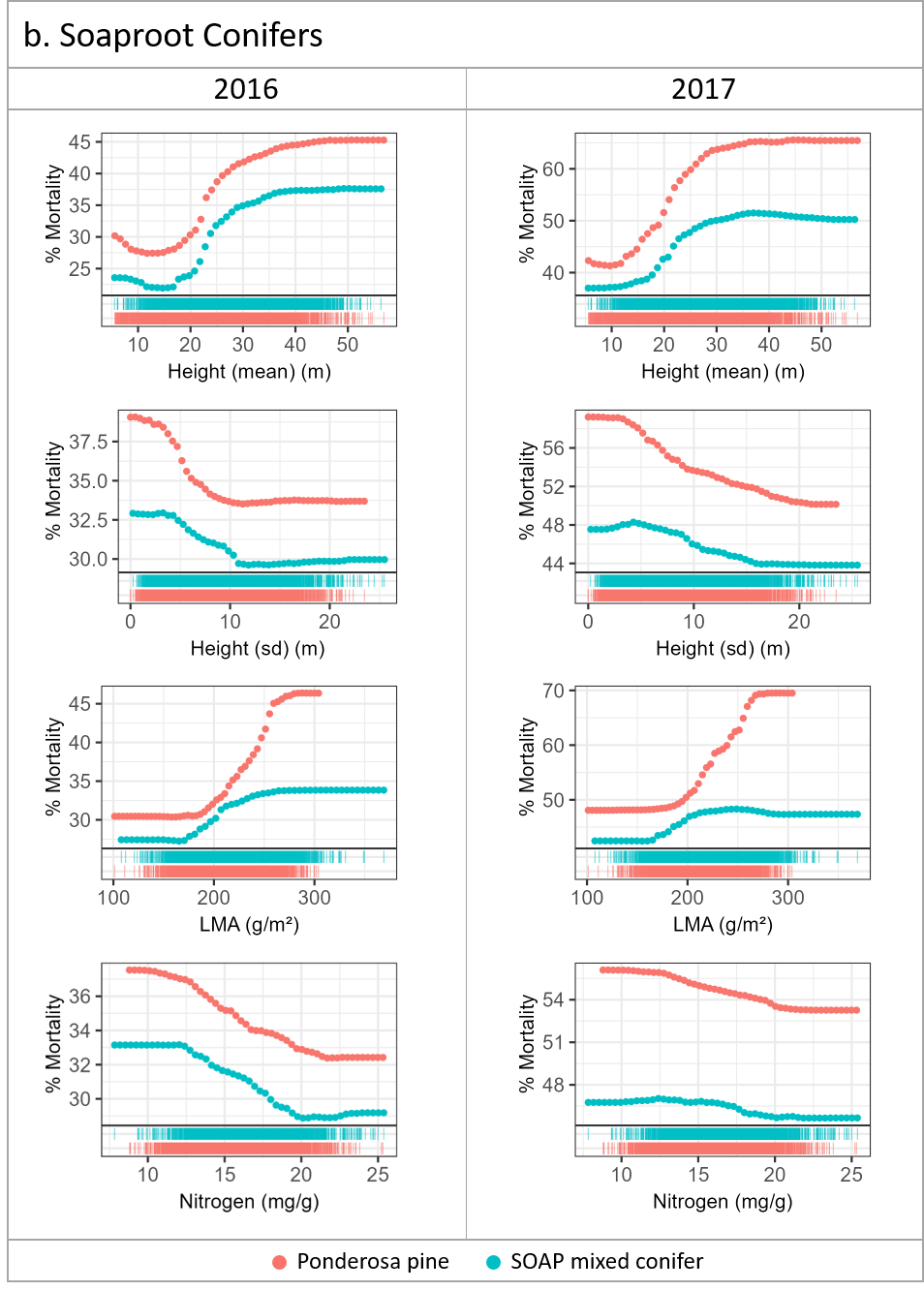

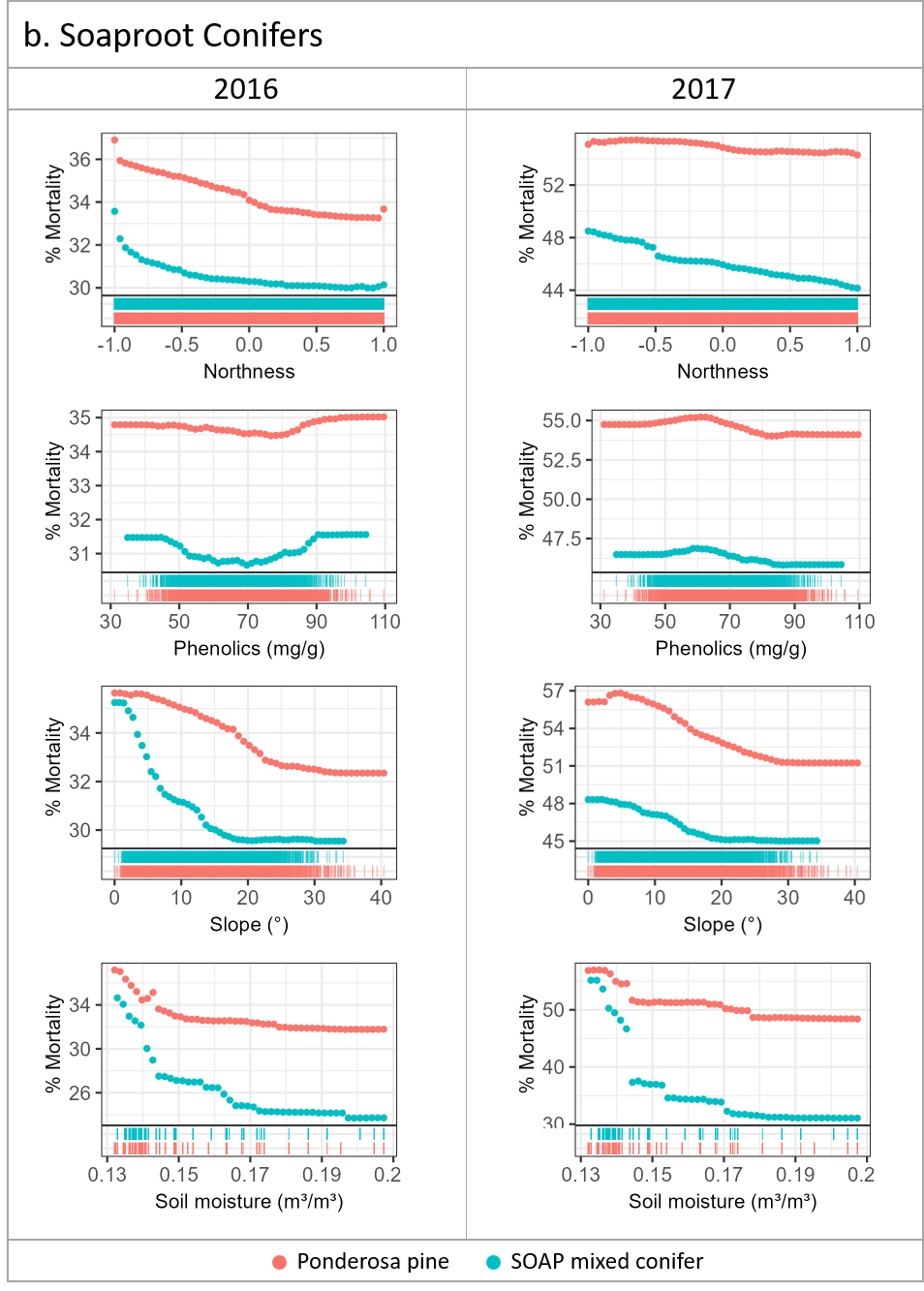

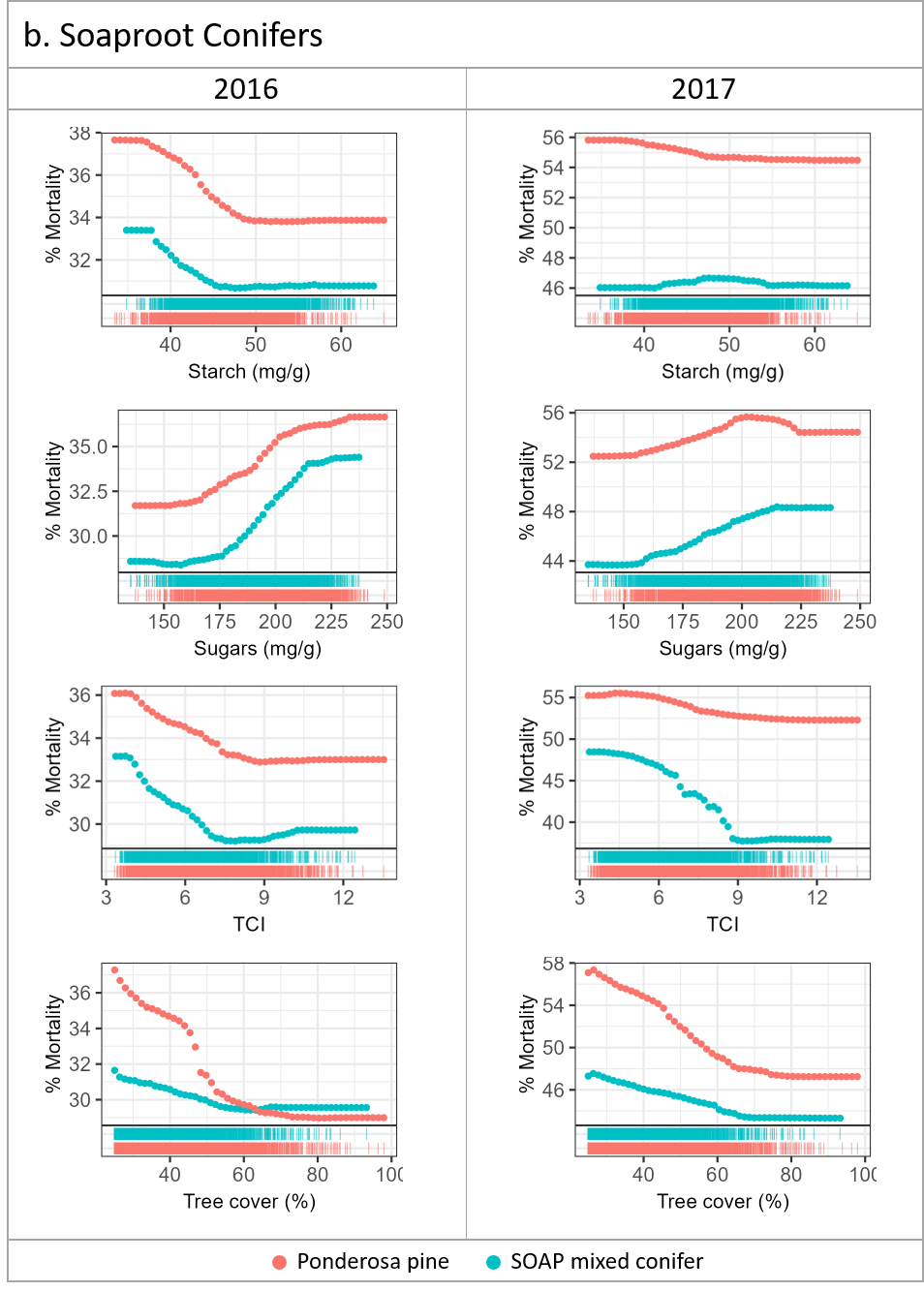

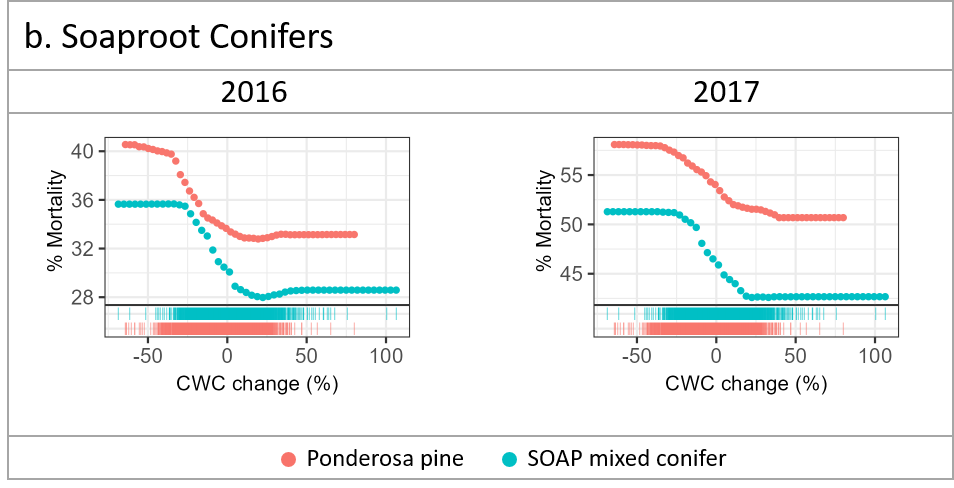


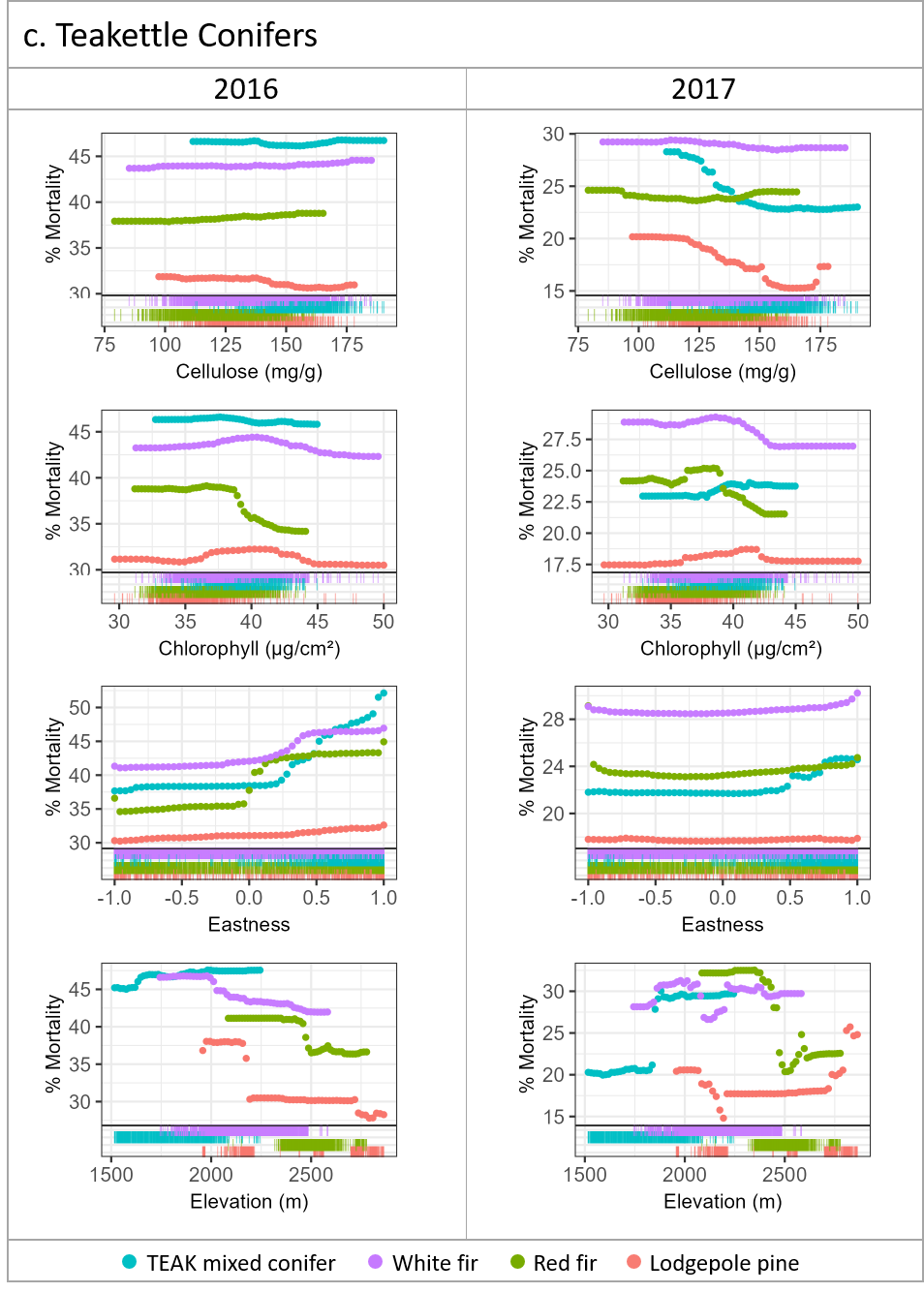

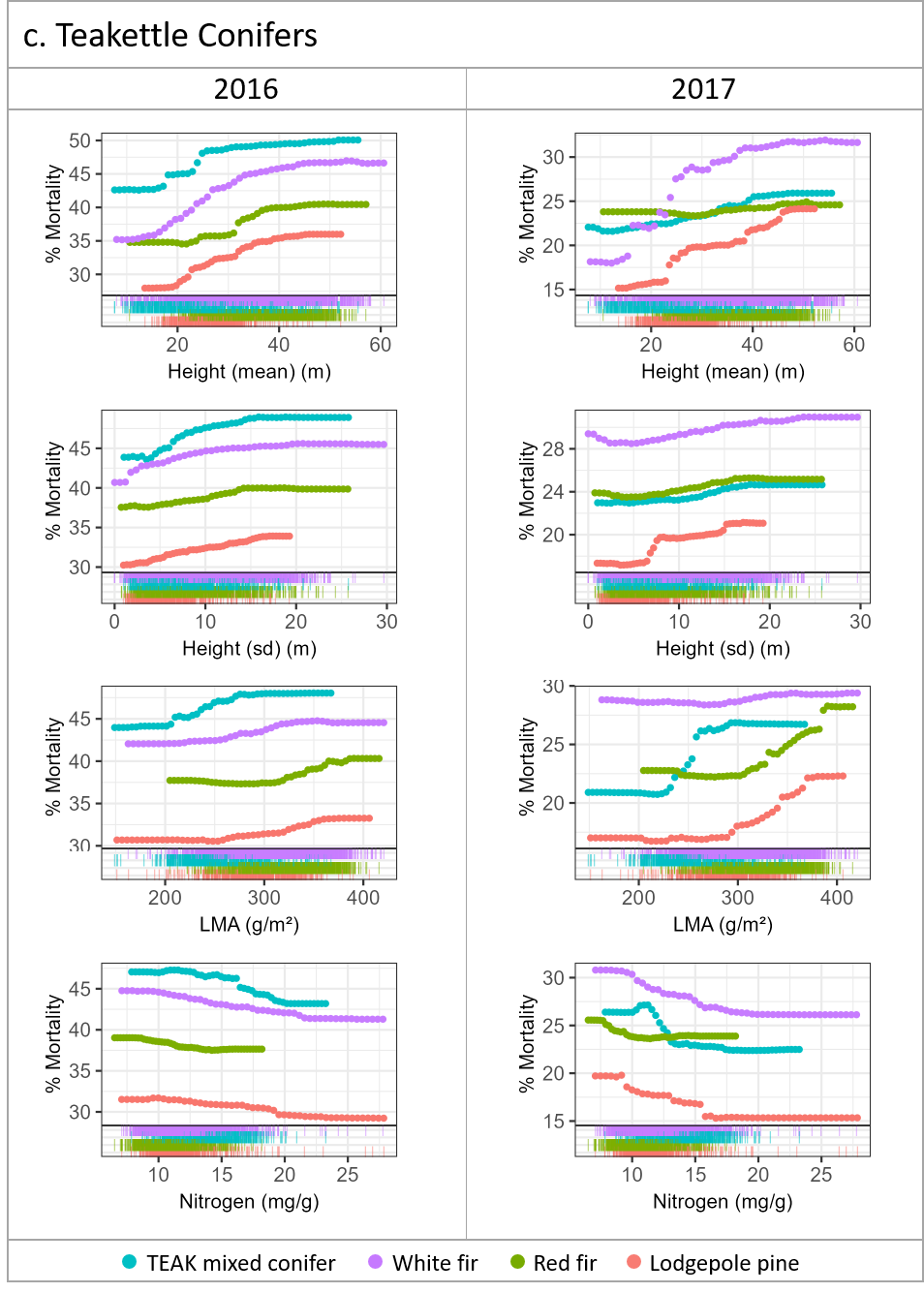

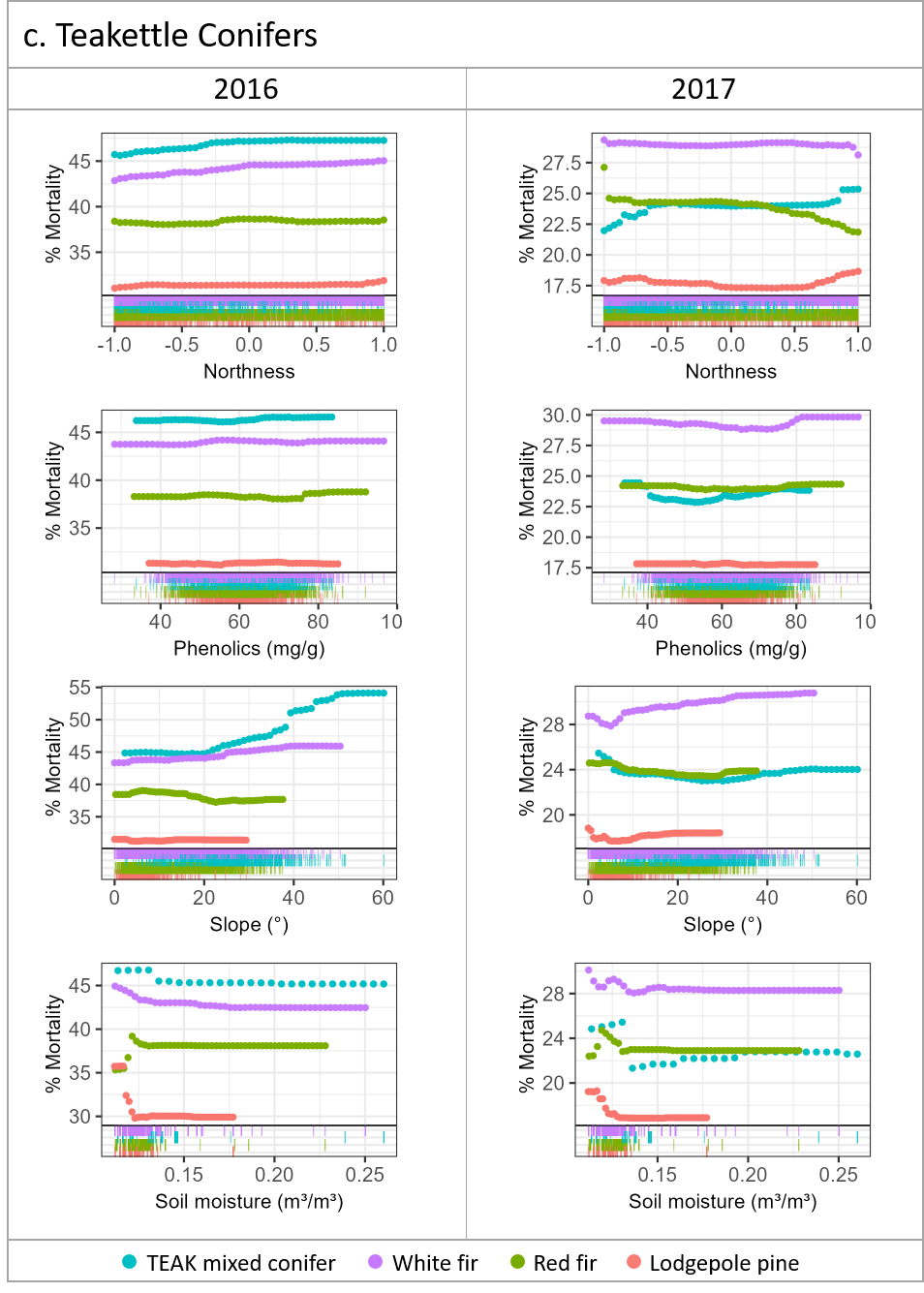

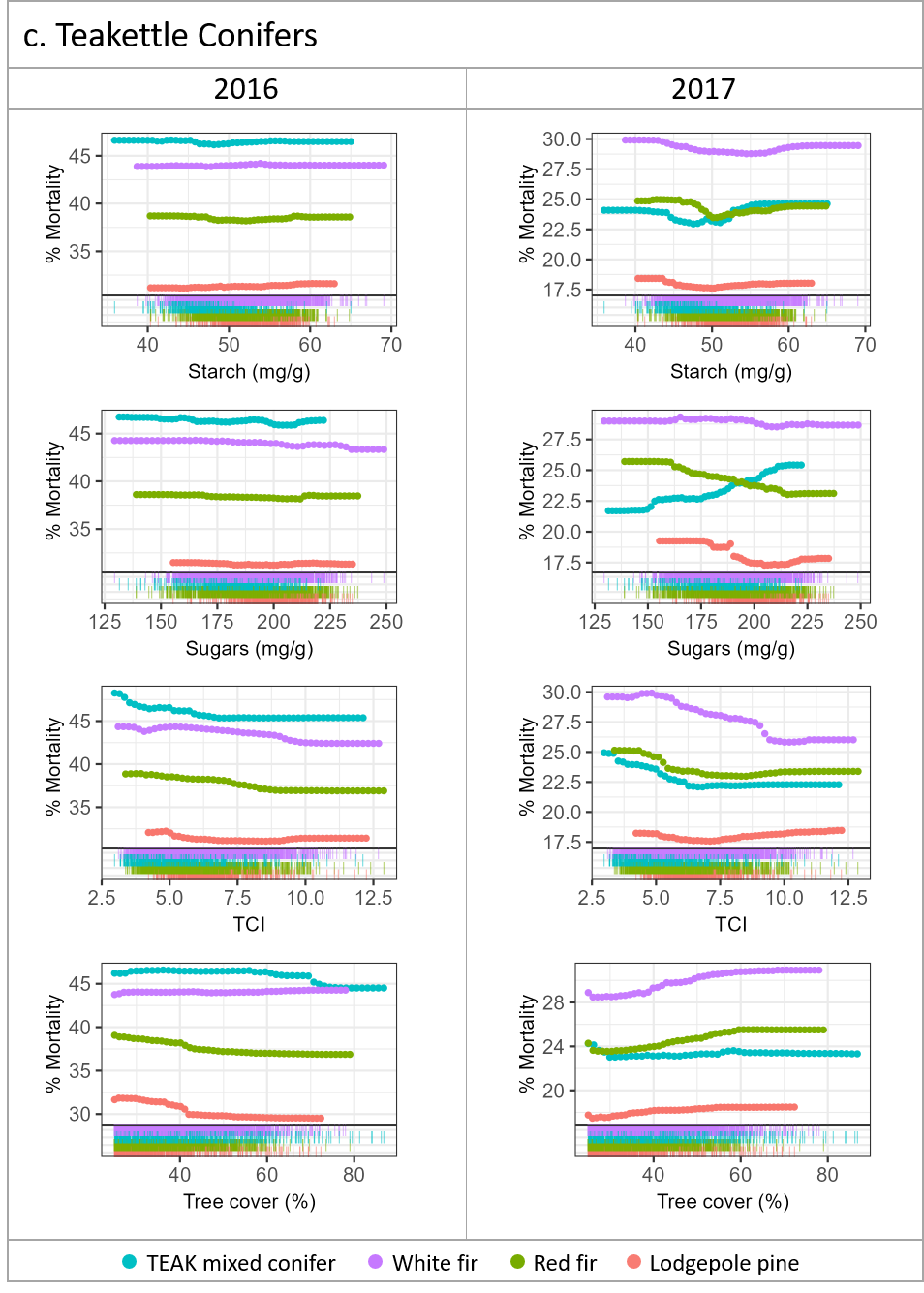

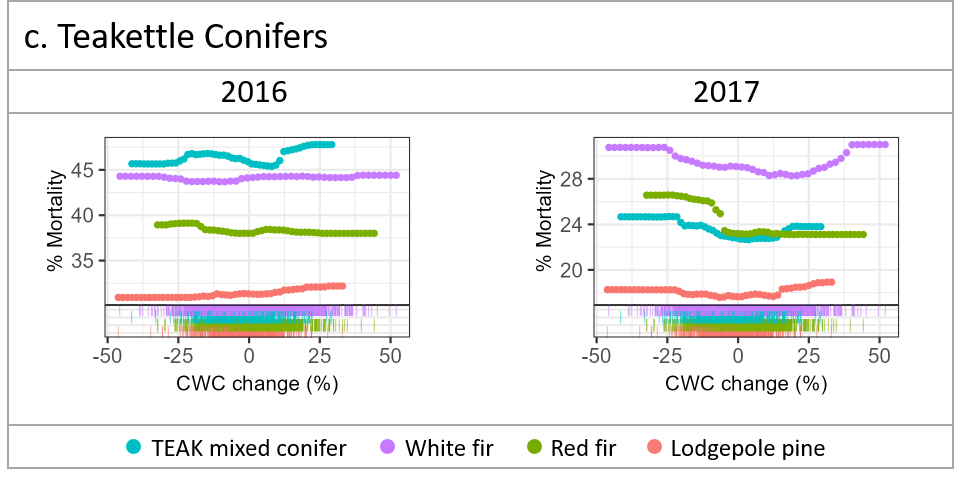


## **Figure S8.** Species level partial dependence plots show mortality trend across all predictor gradients. Accompanying rug plots show species-specific data distributions. Results are shown separately for models using 2016 and 2017 mortality.

## **Table S1.** Trait model performance for trait maps used in this study, with mean *R*^2^ and normalized RMSE across 500 PLSR model permutations.

| **Trait** | **Test set –**  **Mean *R*^2^** | **Test set –**  **Normalized RMSE (%)** |
| --- | --- | --- |
| Cellulose | 0.17 | 15.53 |
| Chlorophyll | 0.54 | 11.33 |
| Lignin | 0.23 | 17.77 |
| LMA | 0.74 | 12.19 |
| Nitrogen | 0.45 | 11.75 |
| NSC | 0.45 | 14.87 |
| Phenolics | 0.35 | 12.10 |
| Starch | 0.35 | 15.09 |
| Sugars | 0.24 | 14.71 |

## **Table S2.** Covariates assembled for drought mortality analysis. Italics denote variable was not used for final analysis.

| **Dataset** | **Variable** | **Units** | **Spatial Resolution** | **Citation** |
| --- | --- | --- | --- | --- |
| Tree mortality | Tree mortality (2016) | % | 1 m | Stovall, A. E. L. (2019). *CA_lidar_tree_mortality* [dataset]. figshare. <https://doi.org/10.6084/M9.FIGSHARE.7609193.V1> |
|  | Tree mortality (2017) | % | 30 m | Hemming-Schroeder, N. M., Gutierrez, A. A., Allison, S. D., & Randerson, J. T. (2023). *Data and code from: Estimating individual tree mortality in the Sierra Nevada using Lidar and multispectral reflectance data* (v1.0.0) [Computer software]. Zenodo. <https://doi.org/10.5281/ZENODO.7812035> |
|  | Tree height | m |  |  |
|  | Tree cover | % |  |  |
| Foliar traits | Nitrogen | mg/g | 30 m | Methodology following:  Singh, A., Serbin, S. P., McNeil, B. E., Kingdon, C. C., & Townsend, P. A. (2015). Imaging spectroscopy algorithms for mapping canopy foliar chemical and morphological traits and their uncertainties. *Ecological Applications*, *25*(8), 2180-2197.  Wang, Z., Chlus, A., Geygan, R., Ye, Z., Zheng, T., Singh, A., ... & Townsend, P. A. (2020). Foliar functional traits from imaging spectroscopy across biomes in eastern North America. *New Phytologist*, *228*(2), 494-511. |
|  | Leaf mass per area (LMA) | g/m^2^ |  |  |
|  | *Lignin* | mg/g |  |  |
|  | Chlorophyll | µg/cm^2^ |  |  |
|  | Cellulose | mg/g |  |  |
|  | *Nonstructural carbohydrates (NSC)* | mg/g |  |  |
|  | Sugars | mg/g |  |  |
|  | Starch | mg/g |  |  |
| Canopy water content (CWC) | CWC | ml | 30 m | Brodrick, P. G., Anderegg, L. D. L., & Asner, G. P. (2019). *Dry-season canopy water content maps for California vegetation from 1990-2017, link to GeoTiffs*. PANGAEA. <https://doi.org/10.1594/PANGAEA.897276> |
| *Climate (2012 – 2016)* | Minimum temperature | ° | 1 km | Thornton, M.M., Shrestha, R., Wei, Y., Thornton, P.E., Kao, S-C., & Wilson, B.E. (2022). *Daymet: Monthly climate summaries on a 1-km grid for North America, version 4 R1* [netCDF, GTiff]. 0 MB. <https://doi.org/10.3334/ORNLDAAC/2131> |
|  | Maximum temperature | ° |  |  |
|  | Mean temperature | ° |  |  |
|  | Cumulative precipitation | mm |  |  |

| *(cont)* | | | | |
| --- | --- | --- | --- | --- |
| **Dataset** | **Variable** | **Units** | **Spatial Resolution** | **Citation** |
| *Climate normals (1970 – 2000)* | Minimum temperature | ° | 1 km | Fick, S. E., & Hijmans, R. J. (2017). WorldClim 2: New 1‐km spatial resolution climate surfaces for global land areas. *International Journal of Climatology*, *37*(12), 4302–4315. <https://doi.org/10.1002/joc.5086> |
|  | Maximum temperature | ° |  |  |
|  | Mean temperature | ° |  |  |
|  | Cumulative precipitation | mm |  |  |
| Digital elevation model (DEM) | Elevation | m | 30 m | van Zyl, J. J. (2001). The Shuttle Radar Topography Mission (SRTM): A breakthrough in remote sensing of topography. *Acta Astronautica*, *48*(5–12), 559–565. <https://doi.org/10.1016/S0094-5765(01)00020-0> |
|  | Slope | ° |  |  |
|  | Eastness | unitless |  |  |
|  | Northness | unitless |  |  |
|  | Topographic Convergence Index (TCI) | unitless |  |  |
| Soil moisture | Soil moisture | m^3^/m^3^ | 30 m | Vergopolan, N., Chaney, N. W., Pan, M., Sheffield, J., Beck, H. E., Ferguson, C. R., Torres-Rojas, L., Sadri, S., & Wood, E. F. (2021). SMAP-HydroBlocks, a 30-m satellite-based soil moisture dataset for the conterminous US. *Scientific Data*, *8*(1), 264. <https://doi.org/10.1038/s41597-021-01050-2> |
| Vegetation species | Vegetation species | classes | vector | USDA - Forest Service. (2016). *Existing Vegetation—CALVEG*. Southern Sierra Region. <https://data.fs.usda.gov/geodata/edw/datasets.php> |

## **Table S3.** Comparison of model performance using trait maps derived from 2013 and 2014 AVIRIS-C imagery. Trait year had minimal effect on model performance.

| **Mortality year** | **Site** | **Trait year** | **R^2^** | **RMSE** |
| --- | --- | --- | --- | --- |
| 2016 | Teakettle | 2013 | .47 | 9.24 |
|  |  | 2014 | .46 | 9.29 |
|  | Soaproot | 2013 | .41 | 14.33 |
|  |  | 2014 | .43 | 14.09 |
| 2017 | Teakettle | 2013 | .31 | 12.53 |
|  |  | 2014 | .31 | 12.53 |
|  | Soaproot | 2013 | .53 | 16.46 |
|  |  | 2014 | .55 | 16.24 |
